# A transcriptional roadmap of the yearly growth cycle in *Populus* trees

**DOI:** 10.1093/plcell/koaf208

**Published:** 2025-08-20

**Authors:** Alice Marcon, Laura García Romañach, Domenique André, Jihua Ding, Bo Zhang, Torgeir R Hvidsten, Ove Nilsson

**Affiliations:** Umeå Plant Science Centre, Department of Forest Genetics and Plant Physiology, Swedish University of Agricultural Sciences, Umeå 901 83, Sweden; Umeå Plant Science Centre, Department of Forest Genetics and Plant Physiology, Swedish University of Agricultural Sciences, Umeå 901 83, Sweden; Umeå Plant Science Centre, Department of Forest Genetics and Plant Physiology, Swedish University of Agricultural Sciences, Umeå 901 83, Sweden; Umeå Plant Science Centre, Department of Forest Genetics and Plant Physiology, Swedish University of Agricultural Sciences, Umeå 901 83, Sweden; Umeå Plant Science Centre, Department of Forest Genetics and Plant Physiology, Swedish University of Agricultural Sciences, Umeå 901 83, Sweden; Faculty of Chemistry, Biotechnology and Food Science, Norwegian University of Life Sciences, Ås 1432, Norway; Umeå Plant Science Centre, Department of Forest Genetics and Plant Physiology, Swedish University of Agricultural Sciences, Umeå 901 83, Sweden

## Abstract

*Populus* species have adapted to many different boreal environments, characterized by fluctuating seasons. The environmental shifts throughout the year trigger molecular responses in trees, regulating crucial developmental processes. To study these molecular responses, we performed RNA sequencing on 207 samples from European aspen (*Populus tremula*) trees grown outdoors during different stages of their annual growth cycle, together with samples from hybrid aspen (*Populus tremula × tremuloides* hybrid T89) trees grown in controlled conditions mimicking seasonal changes in day length and temperature. This created a complete transcriptional roadmap of the yearly growth cycle of *Populus* trees. Co-expression network analyses produced 46 modules, 36 of which show a seasonal expression profile where many aspects were mimicked by indoor samples. However, several modules differed between outdoor and indoor conditions, indicating that important aspects of growth regulation are missed in experiments conducted under controlled conditions. The module networks identify gene hubs involved in season-specific molecular processes of *Populus* trees during the year. To make the dataset easily accessible, we developed POPUL-R (https://lauragarciaromanach.shinyapps.io/popul_r_mini/), a Shiny app enabling users to visualize gene expression data and create interactive networks. POPUL-R will be a valuable tool for the scientific community to explore the role of specific genes in the annual growth cycle of trees.

## Teaser

Seasonal fluctuations induce major transcriptomic changes in aspen trees, revealing how environmental cues regulate their annual growth and adaptation cycle.

## Introduction

*Populus* tree species are widely spread throughout the northern hemisphere and are native to cool temperate and boreal regions of Europe, Asia, and North America (MacKenzie 2010). Due to their extensive distribution, many species have differentiated to adapt to a broad range of environmental conditions and soil compositions. *Populus* trees have a high degree of adaptability and fulfil a fundamental ecological role for other species living in the same regions such as: herbivorous, saprophytic invertebrates, fungi, and lichens. The European aspen (*Populus tremula*) for instance, has more host-specific species than any other boreal tree and is one of the most significant contributors to total epiphyte diversity in the boreal forest (MacKenzie 2010).

Moreover, poplars are economical important species which are used in the production of veneer and pulp for paper, high-quality charcoal and chip-wood (Savill 2013). Additionally, their rapid growth rate makes them an ideal biomass crop for energy production. For instance, *Populus tremula* × *tremuloides*, the hybrid between the European aspen and the North American quaking aspen, is extensively used for wide-scale plantations thanks to its stronger vigor and higher growth rates (Wühlisch 2009).

Poplars' ecological and biological traits, such as their fast growth rate, ecological diversity, and the potential for species hybridization, have made poplars the subjects of molecular genetics and physiological studies (Brunner et al. 2004). In addition, *Populus* species are relatively easy to transform, regenerate and vegetatively propagate, enabling their use in functional genomic studies. Due to these characteristics, genomic and molecular biology resources for this genus have rapidly increased, and in 2006 *Populus trichocarpa* was the first tree to have its genome sequenced (Tuskan et al. 2006).

Thanks to the implementation of NGS techniques, extensive genetic and genomic data have been accumulated, to provide an excellent model for studying how evolutionary processes affect patterns of genetic variation across genomes. Because of their biological and genetic characteristics, and the amount of information available, *Populus* is now a well-established model system for woody perennial plant biology.

The benefits of adopting a model for woody perennial species revolve around the difficulty to study biological processes of specific importance to trees in herbaceous models, such as *Arabidopsis thaliana*. *Populus* trees possess a perennial habit and long-life span, extensive secondary growth from a vascular cambium, wood formation, and mechanisms of adaptation to local environment over large geoclimatic ranges that are typical of many trees. Like for all trees in the temperate and boreal regions, growth and development of *Populus* species must adopt to seasonal changes. In the summer, trees experience a period of vegetative growth which ends with the shortening of the daylength and lowering of the temperature in autumn. Buds are formed to protect the meristem from the upcoming winter conditions; trees enter dormancy and go through a process of cold acclimatation. Once dormancy is released and conditions become favorable again in spring, the buds flush, initiating a new growth cycle.

Numerous studies on different *Populus* species have analyzed the transcriptomic profiles of defined tissues during specific developmental stages, particularly focusing on stress responses. However, these studies often involve trees grown in controlled conditions with artificial settings that only partially replicate aspects of the natural outdoor environment.

With this study we aim to fill this gap by creating a gene expression map of the most drastic molecular changes which occur during the seasonal shifts. Not only is this dataset a complete representation of the growth cycle of aspen trees, but it also includes samples collected from both outdoor and indoor controlled conditions. This allows us to identify which are the most important environmental signals underlying major shifts in transcriptional regulation over the different seasons, and what are the most important changes occurring in outdoor conditions that are nor replicated in controlled indoor conditions. The heterogeneity of the sampling conditions made it possible to identify, through co-expression analysis, 46 modules of which 36 display a clear seasonal expression profile. Each module contains genes that are strongly co-expressed and describe the phenological changes occurring across the seasons throughout the yearly growth cycle of aspen.

To provide an open and interactive way to access the complete dataset we created POPUL-R (https://lauragarciaromanach.shinyapps.io/popul_r_mini/), an app that allows users to plot the expression profile of a single gene or visualize the heatmap of a gene family and to easily generate co-expression networks of the intended genes of interest and their selected neighbors.

## Results and discussion

### Principal component analysis of seasonal samples

**Figure 1.**
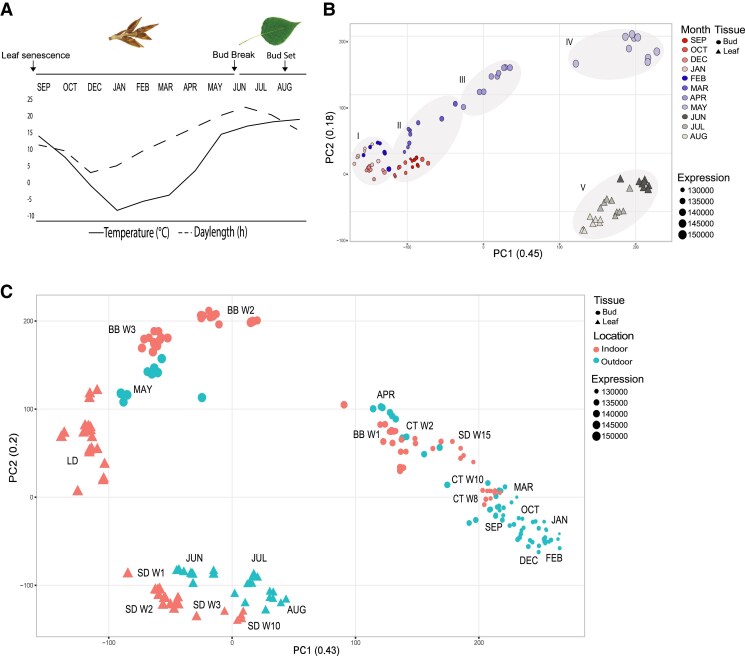
The transcriptome profiles of outdoor and indoor samples change throughout the seasons and different growth conditions. **A)** Average temperature (°C) and hours of light over the sampling period from January to September 2013 (excluding November). Buds from September to May and leaves from June to August. The timing of leaf senescence, bud break and bud set are indicated by arrows; **B)** PCA of the transcriptome of 95 outdoor samples (SEP to AUG). The symbol size indicates the cumulative expression of all transcripts within the sample; **C)** PCA of the transcriptome of both outdoor and 112 indoor samples. SD (short day), CT (cold treatment), BB (bud break), and LD (long day). w (number of weeks of treatment). The symbol size indicates the cumulative expression of all transcripts within the sample (VST-transformed counts).

First, the transcriptomes of 95 samples, collected from aspen trees growing outside in Umeå, Sweden (63.8°N), were analyzed. Samples were collected monthly (Fig. 1A) to create a transcriptional atlas of the annual growth cycle. A first analysis of the data shows distinct seasonal fluctuations across the timepoints, while only minor differences are observed among the sampled trees despite the difference in age (Y, A1, and A2) and genotypes (Supplementary Fig. S1).

The overall variation of the dataset was examined using principal component analysis (PCA). The first two components explain 45% and 18%, respectively, of the variation (Fig. 1B). The first component clusters the samples based on the environmental conditions under which they were collected, while the second accounts for the collected tissue type. We identified five larger clusters which represent major seasonal growth and developmental transitions (Fig. 1B). The samples collected during winter group together in cluster I (Dec, Jan, and Feb). Samples collected during autumn (Sep and Oct) and late winter (Mar) form cluster II. The samples collected in April and May form clusters III and IV. From April to May temperatures increase and buds flush. This developmental phase involves a remarkable transcriptomic change within the buds, which is highlighted in the PCA by the clear separation of clusters III and IV (Fig. 1B). While samples collected in April represent unopen buds, May samples consist of flushed buds from which young leaves start to emerge. The last cluster (V) is represented by leaf samples collected during the summer months and even if they group together, it is still possible to observe smaller subgroups depending on which month the samples were taken. This because, while leaves are still expanding in June, the tree is already entering growth cessation in August, which is reflected in the transcriptome changes. In general, with the gradual rise in temperature and daylength, the total expression of each sample increases (symbol size). Samples collected in winter display a slightly lower overall transcript accumulation than the flushing buds collected in May and the summer leaves.

To decipher which of the seasonal transcriptional changes could be attributed to changes in daylength and temperature and what might be caused by other factors, we added to the dataset 112 samples collected under indoor controlled growth conditions. The samples, both leaves and buds, were collected under different temperatures and light conditions (Supplementary Data Set 1). A new PCA analysis including all 207 samples show that the grouping of the indoor samples mimics a seasonal distribution, matching in most cases outdoor samples collected in similar conditions of light and temperature (Fig. 1C). For instance, samples collected during cold treatment (CTW8 and CTW10) group with samples from March, while samples collected at the end of short day (SDW15) and the beginning of cold treatment (CTW2) cluster with the samples collected in April, together with samples at the initial steps of bud break (BBW1). In April, the natural daylength is closer the number of hours used in SD (14 h light), and the temperature similar to what trees experience in cold treatment (CT) (4 °C). The successive stages of bud break (BBW2 and BBW3) resemble the outdoor samples collected in May when bud break and initial leaf expansion occur. Accordingly, the May samples group in between the bud and the leaf samples collected under controlled long day (LD) conditions. LD leaf samples group separately from the leaves collected outdoor underlying that the tissue developmental stage has a larger effect on the transcriptome than the temperature or daylength conditions under which the samples were taken. The leaves collected in the summer group together with the leaves collected in SD. The samples collected after SDW1 and SDW2 are still in a stage of active growth, grouping together with outdoor samples collected in June, while the samples from SDW3 and SDW10 group together with the samples from July and August, reflecting a gradual transition to growth cessation.

### Co-expression analysis reveals 46 regulatory modules

**Figure 2.**
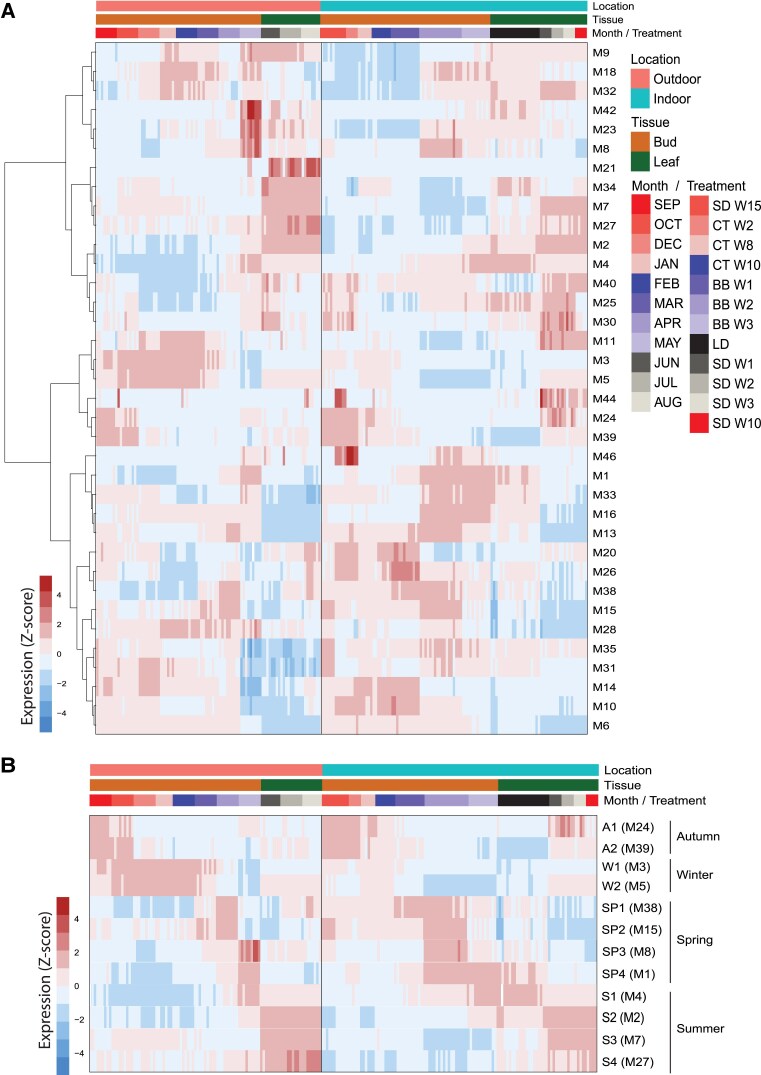
Co- expression analysis generates 46 modules, 36 of which show a seasonal expression profile. **A)** Heatmap of the eigengenes for each of the 36 modules with a seasonal expression profile in all samples. Modules M1 to M46 are numbered in descending order according to the number of genes in each module. For each module, the list of genes and the heatmaps showing the expression profiles can be found in Supplementary Data Sets 2 and 3; **B)** Heatmap of the eigengenes for 12 modules. From the top, modules M24 and M39 are renamed A1 and A2 (autumn); modules M3 and M5, W1 and W2 (winter); modules M38, M15, M8, and M1, SP1 to SP4 (spring); modules M4, M2, M7, and M27, S1 to S4 (summer). The color scale bar represents scaled expression (*Z*-score).

To identify seasonal patterns of gene expression, co-expression analysis was performed on the entire dataset of outdoor and indoor samples. The analysis generated 46 modules (Fig. 2A, Supplementary Data Sets 2 and 3) of which 10 are defined by either location (samples collected indoor or outdoor) or age (young trees from indoor experiments and 1 or 35-yr-old trees from outdoor). The remaining 36 show a seasonal profile.

We performed hierarchical clustering of the eigengenes (representative profiles of each module) of the 36 modules with a seasonal profile to describe and organize the various gene expression patterns during distinct periods of the year (Fig. 2A). Some modules have only minor differences in their expression profile, for instance module M24 and M39. Both modules include genes which are highly expressed during September and October and in bud samples collected in short days. This also reflects the similarity of the conditions between indoor and outdoor samples, already observed in the PCA analysis. Of the 36 modules, we identified 12 representative modules that each has a distinct, time-restricted expression profile and that together cover the entire year, and which includes genes whose expression is high in similar conditions in the indoor samples (Fig. 2B). The first two, module M24 and M39, include genes expressed in autumn and were therefore renamed A1 and A2. The next two modules (M3 and M5) have genes highly expressed in winter, especially in outdoor samples and were renamed W1 and W2. Four modules have specific expression patterns which peak in spring and were renamed SP1 to SP4. Module M38 and M15 includes gene highly expressed in April and the first two weeks of bud break, while modules M8 and M1 peak during May and weeks 2 and 3 of bud break. The last four modules give a good representation of genes expressed in summer and were renamed S1 to S4. Starting with module M4, whose genes are expressed in May and in LD leaves in indoor experiments, the other modules have a gradual shift in expression toward the summer months and leaves in SD. The analysis of the genes belonging to these 12 modules will provide a critical roadmap of the actors involved in some of the most important developmental changes during the growth cycle of aspen trees.

### GO terms and gene network analysis highlight seasonal biological processes and gene hubs

To investigate which biological processes the genes in the modules participate in, GO term enrichment analysis was performed (Supplementary Data Set 4).

**Figure 3.**
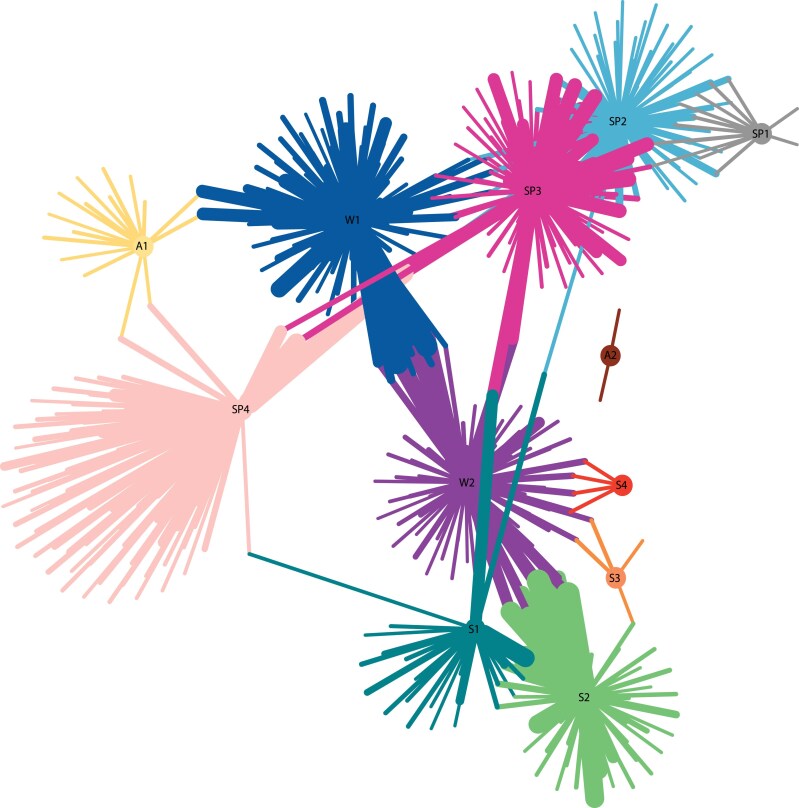
Gene ontology analysis reveals specific GO terms for each module describing the molecular and cellular processes involved in each season. Network of the enriched GO terms for each of the 12 modules; *P*-value threshold, 0.001 (Fisher's exact test). Each colored edge (line) represents a GO term and connected edges show GO terms that are shared between modules. The thickness of the edges is directly proportional to the weight value of enrichment significance of the GO term. See the complete list of GO terms in Supplementary Data Set 4.

We focused on the 12 selected modules and created a network to visualize the relationship between the enriched GO terms (Fig. 3). The modules whose genes are highly expressed in winter (W1, W2), spring (SP1 to SP4), and beginning of summer (S1, S2) contain more enriched GO terms than other modules, many of which are associated to a high number of genes, which underline the significance and complexity of the processes involved during these seasons. Moreover, most GO terms are specific to each module forming defined clusters with fewer GO terms shared among the consecutive seasons.

### Lowering in temperature and daylength triggers cell wall modifications

**Figure 4.**
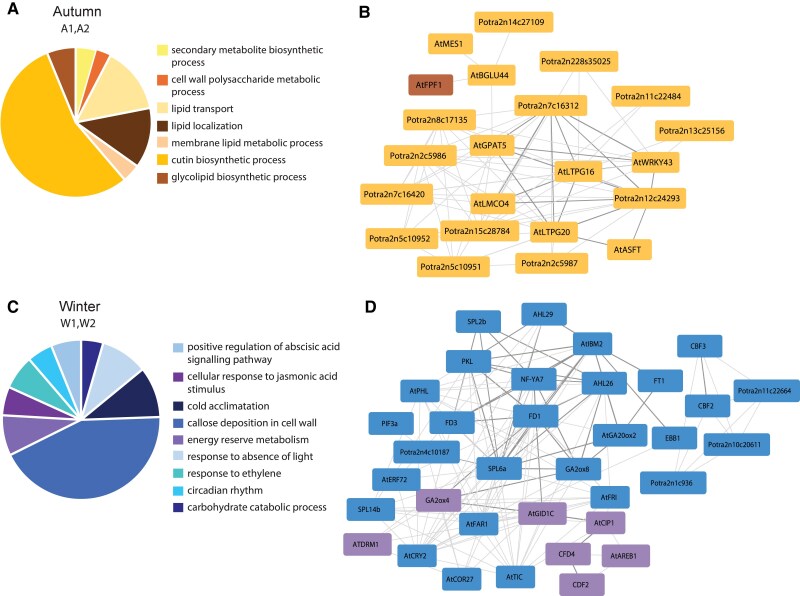
Gene ontology analysis and gene subnetworks for module A1, A2 (autumn), and W1, W2 (winter). The pie charts illustrate some of the most relevant GO terms for A1, A2 **(A)**, and W1, W2 **(C)**. The size of each section shows the significance of the GO term in the module represented by the ratio of the number of genes associated to the GO term in the module over the number of genes associated to the same term in all modules; **B)** Subnetwork of selected genes associated to a relevant GO term from module A1 (yellow nodes) and A2 (brown nodes); **D)** Subnetwork of selected genes from module W1 (blue nodes) and W2 (purple nodes); Genes are named by their ID from the v2 annotation of the *P. tremula* genome or by their gene name. At genes are the corresponding *Populus* homologues of *A. thaliana* genes. Thicker edges highlight the hub of genes discussed in the article. Networks constructed with a 0.3 correlation cutoff.

**Figure 5.**
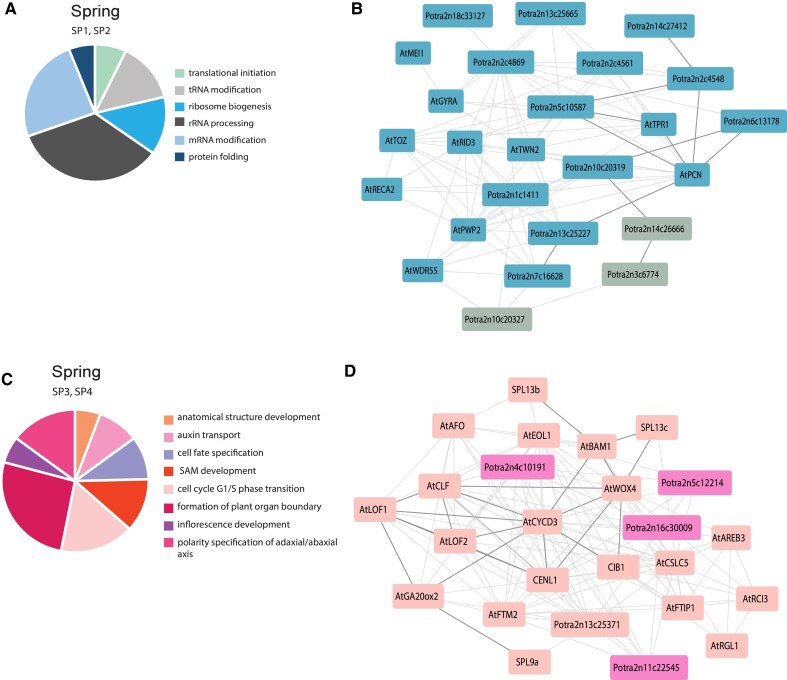
Gene ontology analysis and gene subnetworks for module SP1, SP2 and SP3, SP4. The pie charts illustrate some of the most relevant GO terms for **A)** SP1, SP2 and **C)** SP3 and SP4; **B)** Subnetwork of selected genes from module SP1 (gray nodes) and SP2 (cyan nodes); **D)** Subnetwork of selected genes from module SP3 (magenta nodes) and SP4 (pink nodes). Thicker edges highlight the hub of genes discussed in the article. Networks constructed with a 0.3 correlation cutoff.

**Figure 6.**
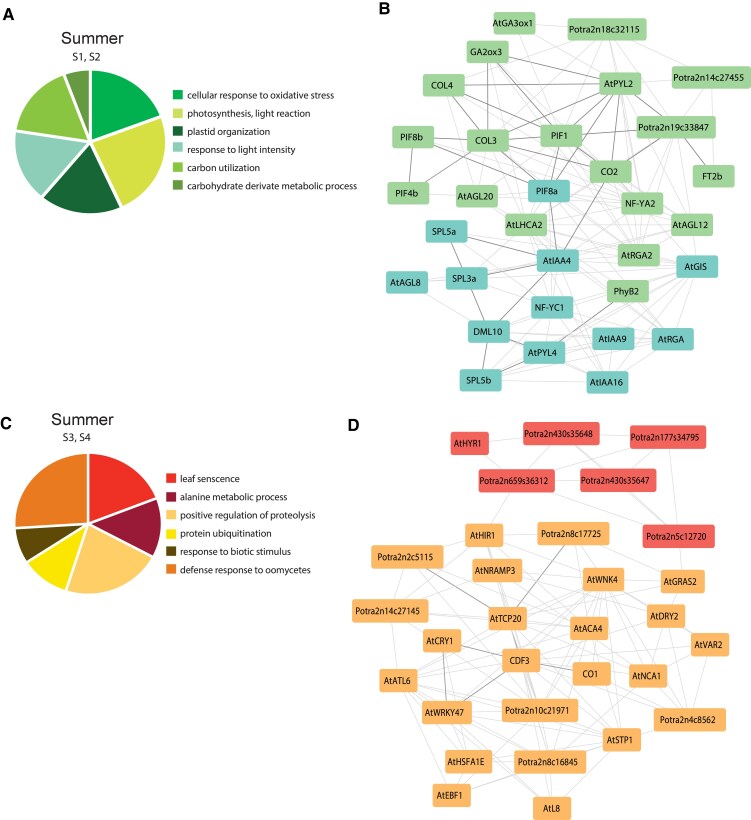
Gene ontology analysis and gene subnetworks for module S1, S2 and S3, S4. The pie charts illustrate some of the most relevant GO terms for **A)** S1, S2 and **C)** S3, S4; **B)** Subnetwork of selected genes from module S1 (teal nodes) and S2 (green nodes); **D)** Subnetwork of selected genes from module S3 (orange nodes) and S4 (red nodes). Thicker edges highlight the hub of genes discussed in the article. Networks constructed with a 0.3 correlation cutoff.

For each season identified by the expression profiles of the 12 modules, we first analyzed the most significantly enriched GO terms and then made a selection based on previous studies, and the genes associated to them (Figs. 4–6, Supplementary Data Set 4). To visualize how their expression is related to one another, we created gene subnetworks for each season selecting the genes which are associated to the selected GO terms or whose function has been previously described in the literature (Figs. 4–6).

Starting with the shortening of daylength and the lowering of temperatures during autumn, trees begin preparing to endure the upcoming harsh winter conditions protecting their tissues from frost damage. In addition to evident phenological changes, such as leaf senescence and bud formation, which already occur at the end of summer, trees implement several molecular changes as well.

The two modules whose genes are expressed in buds during autumn (A1 and A2) and in SD and beginning of CT, are enriched for GO terms such as lipid transport (GO:0006869) and localization (GO:0010876). During autumn, both the membrane and the wall of the cells undergo remarkable changes which increase cell wall thickness and hydrophobicity to adapt to the lowering of temperature and the reduced water availability (Takahashi et al. 2021). We also identified more specific GO terms such as: “cell wall polysaccharide metabolic process”, “membrane lipid metabolic process”, and “cutin biosynthetic process” (Fig. 4A, Supplementary Data Set 4). A joined subnetwork of module A1 and A2 highlights the most relevant genes associated to the enriched GO terms (Fig. 4B, Supplementary Data Set 2). We found homologues of the Arabidopsis genes *Glycerol-3-phosphate acyltransferase 5* (*AtGPAT5*) and *aliphatic suberin feruloyl–transferase* (*AtASFT*), which are part of the biosynthetic pathway of suberin (Hsu et al. 2021b), a lipophilic biopolymer composed of long-chain fatty acids and glycerol (Fig. 4B). Connected to these two genes, as first and second neighbors, is a member of the WRKY family which in Arabidopsis has been shown to regulate polyunsaturated fatty acid content (*AtWRKY43*) (Geilen et al. 2017). In addition, the subnetworks include several esterases/lipases (*Potra2n7c16312*, *Potra2n12c24293*) which play an important role in the polymerization of the cuticle (Shen et al. 2022). The subnetwork also contains several lipid transfer proteins GPI- anchored, in particular, homologues of *AtLTPG16* and *AtLTPG20* which in Arabidopsis, facilitate the movement of lipids between membranes and to the cuticle (Debono et al. 2009). Suberin and cutin are connected with lignin to form a protective barrier in the cell wall (Molina et al. 2009). Consequently, a laccase, *laccase multicopper oxidase 4* (*AtLMCO4*), which contributes to lignin biosynthesis (Wang et al. 2022), is linked to many of the genes in the subnetworks. Altogether, these results reveal a coordinated subnetwork of genes and pathways that enhance lipid-related processes to modify the cell membrane and wall to ensure bud resilience against cold and dehydration. This intricate molecular adaptation underscores the critical role of cell wall remodeling and lipid metabolism in preparing trees for winter dormancy.

### Winter conditions induce hormone signaling involved in dormancy and repressors of vegetative growth

During the following months, the outdoor temperatures drop below zero. The genes expressed during winter and CT belong to modules W1 and W2. In both modules the most enriched GO terms are related to proteolysis (GO:0006508), protein ubiquitination (GO:0016567) and more in general the regulation of macromolecule metabolic process (GO:0060255). During this time of the year, vegetative growth is repressed and trees have entered a dormant state when they focus their energy on coping with harsh environmental conditions. Catabolism of both carbohydrates and proteins occurs to use the resources accumulated over summer in the sink organs. Some of the other most enriched GO terms in these modules refers to response to cold (GO:0009409) and hormones such as: ethylene (GO:0009873), abscisic (GO:0009737), and jasmonic (GO:0009753) acid which in addition to having a role in abiotic stress response, are involved in dormancy (Yordanov et al. 2014) (Fig. 4C, Supplementary Data Set 4). The subnetwork of these two modules includes thousands of genes, of which many are AP2-EREBP (ethylene-responsive element binding proteins) transcription factors (TFs), including *C-repeat/DREB binding factor 2* and *3* (*CBF2, CBF3*) (Fig. 4D). CBFs are key TFs that regulate the expression of cold-regulated (*COR*) gene (Stockinger et al. 1997; Shi et al. 2018). Another gene member of the AP2-EREBP family present in the subnetwork is *EARLY BUD-BREAK 1* (*EBB1*) known to regulate dormancy release (Yordanov et al. 2014), as is *FLOWERING LOCUS T 1* (*FT1*) (André et al. 2022), also present in the same module. The two first neighboring genes of *FT1* are the homologues of the genes *INCREASED IN BONSAI METHYLATION 2* (*AtIBM2*), which in Arabidopsis regulate histone demethylase *AtIBM1* to prevent deposition of heterochromatic silencing marks at transcribed genes with drastic consequences for development (Saze et al. 2008), and *Gibberellin 20-oxidase 2* (*AtGA20ox2*), which is part of the gibberellin biosynthetic pathway (Yamaguchi 2008). In Arabidopsis, AtFT acts in a complex with the B-ZIP TF *FLOWERING LOCUS D* (AtFD) (Abe et al. 2005). In the same subnetwork we find the *FD*-like genes, *FD1* and *FD3* (Sheng et al. 2022). These genes have neighbors such as *PICKLE* (*PKL*), which has been shown to mediate the abscisic acid (ABA)-induced plasmodesmata closure through callose deposition (Tylewicz et al. 2018). Callose deposition in the plasmodesmata interrupts the signaling of molecules which promote growth and leads to the establishment of dormancy in *Populus* trees (Rinne et al. 2011). The GO term “callose deposition in cell wall” was identified in module W1.

Directly linked to *PKL*, we identified genes belonging to the SQUAMOSA PROMOTER BINDING PROTEIN-LIKE (*SPL*) family. Both *SPL2b* and *SPL6a*, present in the subnetwork, are repressed by miR156 (Li and Lu 2014) and their homologues in Arabidopsis have been shown to be involved in the regulation of shoot development; in particular, *AtSPL2* promotes vegetative phase change (VPC) and floral induction (Xu et al. 2016b). These two *SPL* genes are also directly linked to two genes belonging to the AT-hook motif nuclear-localized (*AHL*) family, *AHL26* and *AHL29* (Wang et al. 2021). These genes are closely related to the Arabidopsis gene *AtAHL15*, *AtAHL29*, and *AtAHL20* which repress the expression of *AtSPL2*, *AtSPL9*, and *AtSPL15* during VPC (Rahimi et al. 2022). Interestingly, most of the genes in this subnetwork is linked to the expression of the nuclear factor *NF-YA7* (Liu et al. 2021), possibly suggesting a central role for this factor in coordinating the expression of these genes. More genes in the subnetwork are associated with the repression of growth such as *GA2-oxidases* (*GA2oxs*) which regulate the deactivation of bioactive GAs (Yamaguchi 2008). Specifically, *GA2ox4* has been proven to repress bud break in poplars (Gómez-Soto et al. 2021). Also, related to gibberellin signaling, we identified the homologue of *GIBBERELLIN INSENSITIVE DWARF 1c* (*AtGID1c*), a GA receptor (Nakajima et al. 2006). *AtGID1c* is connected to *COP1-Interacting Protein 1* (*AtCIP1*), an interactor of CONSTITUTIVELY PHOTOMORPHOGENIC 1 (AtCOP1) (Matsui et al. 1995). In Arabidopsis AtCOP1 is negatively regulated by FLAVIN-BINDING, KELCH REPEAT, F-BOX 1 (AtFKF1) which leads to increased CONSTANS (AtCO) stability and the promotion of flowering (Lee et al. 2017). In poplar, it has been shown that FKF1b and FKF1a specifically interact with CYCLING DOF FACTOR 2 and 4 (CDF2, CDF4), respectively, to form a complex with GIGANTEA (GI) to control growth cessation (Ding et al. 2018). In the subnetwork, *CDF2* and *CDF4*, which are highly expressed in buds during SD and CT, are connected to the homologue of *AtCIP1*. The co-expression of these many light-responsive genes shows how it is not only the temperature that influences the transcriptional response in trees during this period to regulate dormancy and growth through several hormone signaling pathways, but daylength plays an important role as well. At these northern latitudes trees need to cope with very few hours of light per day and adjust their metabolism consequently, this adaptation is reflected in the GO terms: “response to absence of light” and “circadian rhythm”, identified in these modules.

### Rising of temperature and daylength causes an increase in the expression of genes regulating transcription and translation

After winter when the environmental conditions are favorable again, trees start awakening. Modules SP1 and SP2 include genes which are highly expressed at the end of winter and in spring and in flushing buds in the controlled conditions. GO terms related to RNA processing (GO:0006396) and ribosome biogenesis (GO:0042254) are the most enriched in both modules (Fig. 5A, Supplementary Data Set 4). During this period, even if no phenological changes can be observed and the buds are still completely unopened, our data indicates an increase in genes regulating transcription and translation.

The GO terms in the modules define a stage in the tree growth cycle when translation is highly active and proteins are produced, ranging from modification of the RNA (GO:0016556, GO:0009451) to protein folding (GO:0006458). Many genes in module SP2 are part of the WD40-like family, which includes several proteins implicated in a variety of functions ranging from signal transduction and transcription regulation to cell cycle control (Van Nocker and Ludwig 2003). The subnetwork generated from genes in these modules includes homologues of *POPCORN* (*AtPCN*) and *TOPLESS-Related 1* (*AtTPR1*) involved in auxin signaling during the organization and maintenance of the shoot and root apical meristems in Arabidopsis (Xiang et al. 2011) (Fig. 5B). Other genes in the subnetwork are putative transcription termination factors such as: *Potra2n3c6774*, *Potra2n7c16628*, and *Potra2n14c26666*, genes involved in ribosome biogenesis (*Potra2n5c10587*, *Potra2n14c27412*, and *Potra2n2c4548*) and genes putatively involved in the initiation of translation (*Potra2n6c13178* and *Potra2n13c25227*). Another gene in the subnetwork which operate as a link among the aforementioned genes is *Potra2n10c20319*, with homology to RNA helicases whose activity is required to “melt” extensive mRNA 5′ UTR secondary structures and allow translation (Parsyan et al. 2011). In summary, the gene expression patterns during late winter and early spring highlight a preparatory phase marked by heightened transcriptional and translational activity, setting the stage for active growth.

### Bud flush is followed by the expression of meristem identity genes

When the temperatures rise in spring, the tree buds eventually flush, and the newly formed shoots start elongating. In the shoot axillary meristems, floral transition will occur as well. The environmental conditions mentioned above correspond to the samples collected in May and at different stages of bud flush in LD indoor samples. Modules SP3 and SP4 include genes expressed during this period, with the latter being the largest module which reflects the complexity of the molecular mechanisms involved to control this developmental phase. The most enriched GO terms in module SP3 are still related to the biogenesis of RNA (GO:0042254) and translation (GO:0006412) while the most enriched GO terms in module SP4 are associated with the cell cycle (GO:0007049), its regulation and phase transitions (GO:0022402, GO:0044770) (Fig. 5C, Supplementary Data Set 4). It is during this period that flower transition occurs, and some of the axillary meristems mature into inflorescences. This transition is related to temporal and spatial changes of growth at the meristems and defined by a strong mitotic activity (Kwiatkowska 2008). Thus, we identified GO terms such as: “SAM development”, “cell fate specification”, and “inflorescence development”. A crucial gene for the determination of meristem identity in Arabidopsis is *TERMINAL FLOWER 1* (*AtTFL1*), which plays an antagonistic role to *FT*/*TWIN SISTER OF FT* (*TSF*) (Bradley et al. 1997). In the gene subnetwork of modules SP3 and SP4, we identified the *AtTFL1* homologue *CENTRORADIALIS-LIKE1* (*CENL1*), which when mutated induces early flowering in *Populus* species (Sheng et al. 2023). Playing such a crucial role in the process, *CENL1* has many co-expressed genes with functions in meristem initiation and maintenance, as well as organ patterning (Fig. 5D). Among these are the homologue of *CURLY LEAF* (*AtCLF*), which in Arabidopsis, is required for stable repression of *AGAMOUS* (*AtAG*) and *AtAP3* and has a putative role in cell fate determination (Zhang et al. 2022), and the homologues of *LATERAL ORGAN FUSION 1* and *2* (*AtLOF1*, *AtLOF2*), which in Arabidopsis, encode for MYB-domain TFs expressed in organ boundaries (Lee et al. 2009). They sort under the GO term “formation of plant organ boundary” and the more generic term “anatomical structure development”. In addition to the determination of organ boundaries, the SAM generates a signal which is required for the polarity specification of the abaxial/adaxial axis in the leaf (Yamaguchi et al. 2012). Many meristem-derived positional signals are dependent on the plant hormone auxin (Heisler et al. 2005), and we identified the GO term: “auxin transport” in the enrichment analysis of the module.

While *CENL1* and its neighboring genes play a role in the maintenance of meristem identity, others are homologues of genes involved in the vegetative to reproductive phase transition, such as *AtSPL9* and *AtSPL13* (Xu et al. 2016b). *SPL13b* and *SPL13c* are indirectly linked together through the homologue of *BARELY ANY MERISTEM 1* (*AtBAM1*) which encodes a *CLAVATA1*-receptor kinase-like protein, which in Arabidopsis is required for both shoot and flower meristem function (DeYoung et al. 2006), *SPL9a* is instead connected to the homologue of *AtGA20ox2*, that in Arabidopsis, as part of the gibberellin biosynthetic pathway together with AtSPL9 and DELLA proteins control axillary bud formation (Zhang et al. 2020). Central in the subnetwork with many connections to neighboring genes we identified the homologues of the cell cycle regulator *Cyclin D3* (*AtCYCD3*) and *WUSCHEL-RELATED HOMEOBOX 4*. (*AtWOX4*), which in Arabidopsis are important for determining cell number in developing lateral organs (Collins et al. 2015) and controlling vascular cambium meristem identity, respectively (Youngstrom et al. 2022). First neighbor of both genes is *CRYPTOCHROME-INTERACTING BASIC-HELIX-LOOP-HELIX 1* (*CIB1*), whose orthologue in Arabidopsis is related to flowering and regulates *AtFT* expression through interaction with CRYPTOCHROME 2 (AtCRY2) and additional CIB1-related proteins (Liu et al. 2018). These results highlight the intricate coordination of gene regulatory subnetworks that govern meristem identity and organogenesis thorough the integration of hormonal signaling and the regulation of the cell cycle during spring bud flush.

### Summer, a period of vegetative growth regulated by light-responsive genes

After bud flush, trees experience the most intense period of active growth which occurs at the beginning of summer when the days are long and warm. Modules S1 and S2 display a clear pattern in which genes are expressed during summer in the outdoor samples and in flushed buds and leaves in the indoor ones. The most enriched GO terms associated with these modules describe an active organism where photosynthetic processes (GO:0015979, GO:0019684, GO:0009657), and the generation of precursor metabolites (GO:0006091) are predominant (Fig. 6A, Supplementary Data Set 4). The gene subnetwork for these modules includes many TFs, such as members of the *SPL* family (Fig. 6B). *SPL3a*, *SPL5a*, and *SPL5b* found in module S1 have previously been associated with regulation of vegetative growth, acting in the promotion of growth cessation (Liao et al. 2023). Strongly linked to the *SPL* genes but with an antagonistic function, DEMETER*-LIKE 10* (*DML10*) DNA demethylase also shows a similar expression profile (Conde et al. 2019). In fact, it has been previously suggested that a progressive reduction of genomic DNA methylation after dormancy release promotes the reactivation of growth (Conde et al. 2019). As linking modules to S2 and S1, we identified several members of the *PHYTOCHROME INTERACTING FACTOR* (*PIF*) family, *PIF1*, *PIF8a* and *PIF8b* which contain an active phytochrome binding (APB) domain to mediate interaction with light-activated Phytochrome B (PhyB), a mechanism conserved across many species (Pham et al. 2018). In the same module, *PhyB2* is also present, and has been shown to act as a suppressor of SD-induced growth cessation in hybrid aspen (Ding et al. 2021). Together with *PIF8* genes, *PIF4b* is also part of module S2. But while both *PIF4* and *PIF8 genes* contribute to the regulation of shoot elongation, only *PIF8a* has a major role in regulating seasonal growth (Ding et al. 2021). In the same subnetwork, *PIFs* are connected to the *CO*-like genes. In Arabidopsis, *AtCO* and *AtPIF7* have an additive effect on shade-induced flowering. Under shade, de-phosphorylated AtPIF7 and accumulated AtCO, upregulate the expression of *AtSPL*s to accelerate flowering (Zhang et al. 2019). Although a phylogenetic analysis of *Populus* PIF proteins suggested that *Populus* lacks an AtPIF7 homologue, the PIF8a protein resembles Arabidopsis AtPIF7 in being stable in response to red or far red light (Zhang et al. 2019).

Linking some of these genes together, we identified genes involved in hormone response (GO:0032352). Additionally, to its connection to *PIF* genes CONSTANS LIKE 3 (*COL3*) (Li et al. 2020) is the first neighbor of *Gibberellin 2-oxidase* (*GA2ox3*) (Gómez-Soto et al. 2021), which in Arabidopsis regulates plant growth by inactivating endogenous bioactive gibberellins (GAs) (Li et al. 2019). We identified also genes involved in auxin signaling such as the homologues of *INDOLE-3-ACETIC ACID INDUCIBLE 4* (*AtIAA4*) (Dreher et al. 2006) and *PYL ABA receptor 2* (*AtPYL2-4*), which function as abscisic acid sensors instead (Santiago et al. 2012). Moreover, in response to the high intensity light (GO:0009642) which the leaves are exposed to, genes controlling the response to oxygen radicals (GO:0000302) are expressed during this time of the year. These results illustrate the dynamic interplay between light perception and hormonal signaling that controls active vegetative growth during summer. The coordinated expression of photosynthesis-related genes alongside growth-promoting and growth-modulating factors underscores the complexity of sustaining a rapid reactivation of growth in response to an increase of daylength and temperature.

### SD conditions at the end of summer trigger genes involved in leaf senescence and stress tolerance

At the end of summer growth cessation occurs, leaves go through senescence and the metabolism slows down. The precise timing of these responses is pivotal for the tree survival and in module S4, signaling and cell communication are the most enriched GO terms (Supplementary Data Set 4). Genes which are expressed at the end of summer as well as in SD leaves in indoor samples, are associated with GO terms such as: “leaf senescence” and “positive regulation of proteolysis” (Fig. 6C). Senescence-associated proteolysis in plants is essential for mobilization of nutrients from old or stressed tissues, mainly leaves, to sink organs (Diaz-Mendoza et al. 2016). Central in the subnetwork, generated from modules S3 and S4, is the member of the *CDF* family, *CDF3* (Fig. 6D), which is homologous to the Arabidopsis genes *AtCDF1-3* (Ding et al. 2018). *AtCDF3* in Arabidopsis is involved in processes such as drought and osmotic stress tolerance and flowering (Corrales et al. 2017), while *CDF3*-overexpressing lines in poplar display an early growth cessation phenotype (Ding et al. 2018). As first neighbors of *CDF3* in the same subnetwork we identified *CO1* (Hsu et al. 2012) and the homologue of *AtCRY1*. AtCRY1 represses gibberellin signaling through the interaction with DELLA proteins and also influences the regulation of *AtCO* (Xu et al. 2016a). Connected directly to *CDF3* and At*CRY1* is the homologue of *AtWRKY47* that modulates leaf senescence through regulating PCD-associated genes in Arabidopsis (Cui et al. 2024). *Potra2n8c17725* and *Potra2n2c5115* in the subnetwork are also associated to programmed cell death and are linked to *CDF3* through the homologue of *TEOSINTE BRANCHED1*, *CYCLOIDEA*, *and PCF 20* (*AtTCP20*). TCP proteins are TFs involved in the regulation of plant growth and development and Arabidopsis loss-of-function mutants of *TCP20* cause earlier senescence (Noh et al. 2021). In addition, we identified GO terms associate to biotic stimulus (GO:0009617), and in particular oomycetes (GO:0002239) which thrive in the humid conditions of end of summer and beginning of autumn. Biotic stresses trigger the plant immunity response to which “protein ubiquitination” contributes crucially (Marino et al. 2012). These results emphasize how at the end of summer trees suffer an intense period of stress from both abiotic and biotic stimuli. The prominence of genes regulating programmed cell death, proteolysis, and pathogen defense highlights the dual necessity of nutrient recycling and resilience against biotic stresses during this critical seasonal transition.

### Differential expression analysis highlights the difference between indoor and outdoor grown trees

Growth chambers and greenhouses are controlled environments used to partially replicate outdoor conditions under consistent, manageable settings. But how accurately do these controlled conditions replicate the natural environment, and what are the largest differences between the transcriptomes of indoor- and outdoor-grown trees?

The PCA analysis of the dataset shows a considerable similarity in the transcriptome profiles of outdoor and indoor samples, with samples taken in controlled conditions following the seasonality of the field samples.

**Figure 7.**
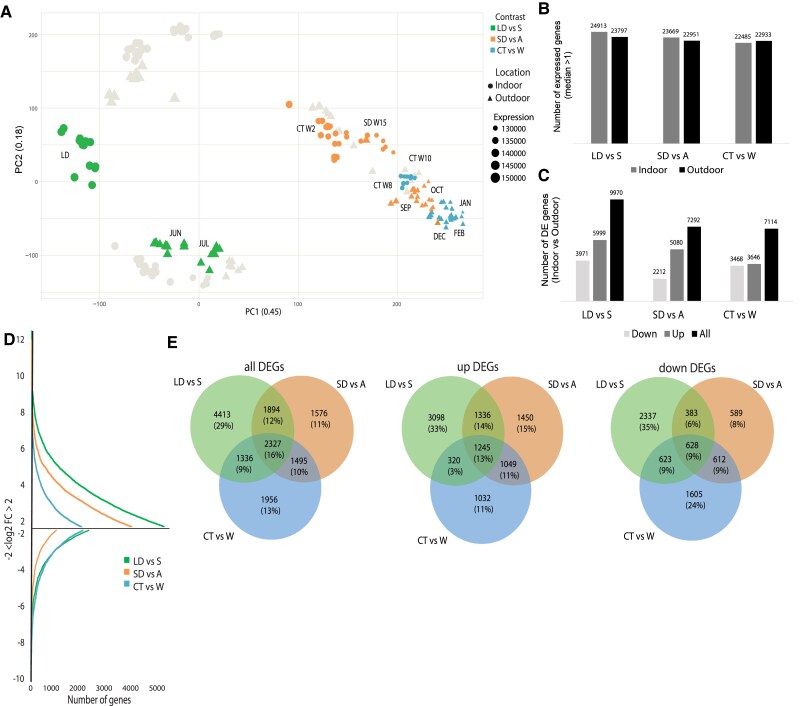
Differential expression analysis between indoor and outdoor conditions. **A)** PCA analysis of the transcriptome of all samples with a focus on LD and June and July; SD and September and October; CT and December, January, and February. The symbol size indicates the cumulative expression of all transcripts within the sample (VST-transformed counts); **B)** number of expressed genes with median >1.0 for each condition; **C)** number of differentially expressed genes in indoor compared to outdoor samples (DESeq2, log2(fold change) >1.0, FDR-adjusted *P*-value < 0.05). The lists of all differentially expressed genes for each contrast are found in Supplementary Data Set 5; **D)** frequency of high DEGs in all contrast (−2 < log2FC > 2); **E)** Venn diagrams showing the number of overlapping differentially expressed genes between the contrasts and their percentages compared to the total number of upregulated (up), downregulated (down) or all genes.

The controlled settings adopted in our growth chambers rotate from a period of LD, allowing the plants to grow; to short day (SD), inducing the process of growth cessation; to CT. To test how faithfully the transcriptomes in controlled settings match the transcriptomes in outdoor environments, we performed differential gene expression analysis for each of the indoor settings. We tested LD samples with leaves collected in June and July; SD and beginning of CT samples (SDW15 and CTW2) against the ones collected in September and October and finally CT samples (CTW8 and CTW10) against the samples collected in winter (Dec, Jan, and Feb) (Fig. 7A). In addition, to minimize the difference between the indoor and outdoor samples, we selected the outdoor samples from the 1-yr-old tree only. The overall number of expressed genes in each condition was comparable and range from 22,485 in CT to 24,913 in LD samples with a fluctuation between 3% and 7% from the median number of expressed genes for all conditions (Fig. 7B). The contrast with the most differentially expressed genes is LD versus summer, followed by SD versus autumn, and last CT versus winter (Fig. 7C). For each contrast there are more upregulated than downregulated genes in indoors samples compared to the outdoor ones (Supplementary Data Set 5). The most upregulated genes are expressed in LD and SD, while the most downregulated genes are expressed in SD and CT (Fig. 7D). This suggests that indoor LD conditions have a stronger effect in inducing gene expression than the outdoor summer conditions, while the opposite is true for CT conditions versus winter outdoor temperatures. The extreme winter conditions which trees experience outdoors have a stronger effect on gene expression than the indoor settings.

Considering the high number of differentially expressed genes, we focused our attention on the genes specifically differentiated in only one condition. Among the upregulated genes in indoor conditions 15% of the genes were specifically differentially expressed in SD, 11% in CT, and 33% in LD (Fig. 7E). Also, for the downregulated genes the condition with the highest number of specific differentially expressed genes is LD (35%), with CT at 24% and SD only at 8%. In general, there is a high number of genes (16%) which are differentially expressed in all the contrasts. Those genes are differentially expressed in indoor conditions besides temperature and light settings, which are the two major settings which change between the treatments.

To get an overview of the biological and molecular processes regulated by the differentially expressed genes in each contrast (both indoor and outdoor), we performed GO term enrichment analysis (Supplementary Data Set 6). There were many GO terms associated to differentially expressed genes, in particular in LD and CT, but again we focused only on the specific GO terms relative to one contrast (Fig. 7E).

**Figure 8.**
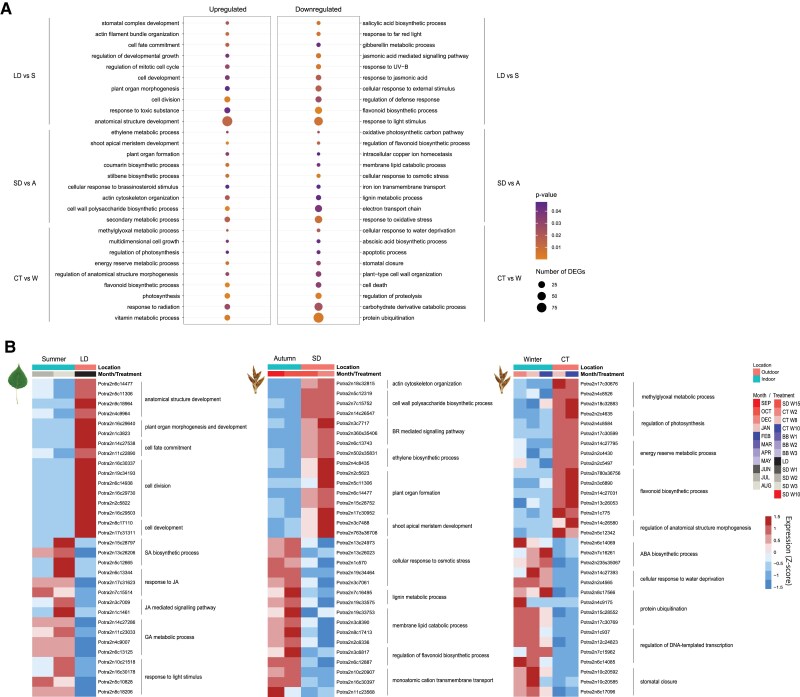
Differential expression analysis between indoor and outdoor conditions reveals specific GO terms and DEGs for each contrast. **A)** Specific GO terms associated with DEGs in each contrast, *P*-value < 0.05. The dot size corresponds to the number of DEGs associated with the GO term; **B)** Selected DEGs with associated significant GO terms for each contrast. The icons on the top left of each heatmap represent the sample tissue used in each comparison. S: summer, A: autumn, W: winter. The color scale bar represents scaled expression (*Z*-score).

Based on the transcriptomic profile, the PCA analysis highlights a significant difference between the samples collected in LD and the leaves collected in summer, even though the 18 h of light to which the indoor grown trees are subjected match approximately the natural daylength of the summer months. Instead, LD samples group closer to the samples collected in May. Even though the leaves sampled in LD are fully expanded, the tissue is relatively young and presumably more similar to the leaves emerging from the newly developed shoot in May than the leaves collected in June. GO terms associated with the upregulated genes indicate an intense mitotic activity and “cell development” (Fig. 8A). Among highly differentiated genes we identified a putative cyclin (*Potra2n16c29503*), homologues to cyclin interacting proteins (*Potra2n16c30337*, *Potra2n19c34193*) and several members of the actin family (Fig. 8B).

The GO terms associated to the differentially expressed genes outdoor (downregulated GO terms in indoor versus outdoor comparison) describe an environment where trees are under stress and a cellular defense response is triggered. Plant hormones have a significant role in the response to both abiotic and biotic stresses, and in the list of terms we identified GOs related to the jasmonic (JA) and salicylic acid (SA) pathways. Differentially expressed genes associated to these terms are part of Scarecrow-like proteins and TIFY which are involved in the response to JA stimulus (Chung and Howe 2009). Moreover, the role of JA in plant response to abiotic stresses such as UV radiation has been extensively investigated (Liu et al. 2012). “Response to UV-B”, “response to far-red light”, and other terms related to light stimulation are also associated to the differentially expressed genes in outdoor samples in summer. These terms highlight the differences in the light spectrum between the lamps used in the growth chambers and natural light. The lamps used scarcely emit in the UV-B spectrum and the Red-to-Far Red ratio is higher than what the trees experience outdoors.

The second contrast compares buds collected in SD conditions (SDW15) and at the beginning of CT (CTW2) with the samples collected in September and October. As for the previous comparison, the transcriptome profiles of the autumnal samples have no correspondence in indoor conditions. Buds collected in SD and beginning of CT group instead with samples collected in April, during which the natural daylength is closer to the number of hours used in SD (14 h light), and the temperature is similar to what trees experience in CT (4 °C). During autumn daylength varies from 11 to 9 h of light.

Like for the samples collected in LD, the GO analysis depicts an environment where trees in SDs are growing more actively than the ones growing outdoors. We identified GO terms such as: “plant organ formation” and “shoot meristem development”. Among the genes associated to these terms we identified putative members of the TCP family (*Potra2n15c28752*, and *Potra2n17c30952*) (Fig. 8B).

The differentially expressed genes which were upregulated outdoor instead, indicate a strong response to osmotic and oxidative stress. Compared to indoor trees, which never experience water shortage, trees outdoor may experience water deprivation which would trigger a drought stress response which involves osmotic and oxidative pathways (Pugnaire et al. 1999). We identified many genes with oxidoreductase activity, such as genes homologous to peroxidases and a superoxide dismutase (*Potra2n13c24973*, *Potra2n13c26023*, and *Potra2n19c34464*).

The last comparison we performed investigated the differentially expressed genes between the samples collected at the end of CT (W8 and W10) and winter samples collected from December to February. The extreme natural conditions that trees experience at northern latitudes makes the transcriptome profiles of the outdoor winter samples very different from the samples collected after indoor CT. The cold-treated trees were exposed to a temperature of +4 °C, which is higher than what trees experience outdoor during December to February when the temperature is constantly below 0 °C. The cold-treated trees grouped together with outdoor samples collected in March when the average temperature is still colder than the indoor settings but probably shows some warmer peaks during the days resembling more what trees experience in the growth chamber.

In indoor samples we identified many upregulated genes with GO terms related to photosynthesis and its regulation, as well as response to radiation and production of flavonoids. This might suggest that the trees are experience a light stress in the indoor growth chambers. Flavonoids are known to protect plants against high light exposure thanks to their antioxidant properties (Ferreyra et al. 2021). The genes involved in the biosynthesis of these compounds are among the most upregulated ones compared to outdoor samples. We identified genes homologous to a chalcone synthase (*Potra2n3c6890*), two UDP-glucosyl transferases (*Potra2n780s36756*, *Potra2n14c27031*) and a flavanone hydrolase (*Potra2n13c26053*).

The GO terms associated to differentially expressed genes in outdoor samples (downregulated GO terms in indoor versus outdoor comparison) were related to the catabolic pathways of both carbohydrates and proteins which are triggered to allow trees to exploit the resources stored in sink organs to survive the harsh winter conditions. Other GO terms enriched for this contrast are related to the biosynthesis of abscisic acid, which is correlated with the maintenance of dormancy (Ding et al. 2024); and “stomatal closure” which is triggered under water-deficit conditions (Hsu et al. 2021a). Among the most differentially expressed genes we identified TFs belonging to the AP2-EREBP family, of which two *CBF* genes (*Potra2n1c937* and *Potra2n12c24823*) are associated with cold response.

In conclusion, the biggest differences between trees grown indoors and outdoors appears to be related to milder environment conditions, higher availability of nutrients and more aseptic premises in growth chambers than in nature where trees are exposed to more adverse climatic conditions and pathogens. These findings underline that only matching photoperiod and temperature in controlled environments is, unsurprisingly, insufficient to replicate natural conditions at the molecular level. The observed differences, in particular in relation to stress response, highlight that data derived exclusively from controlled environments will not fully capture the complexity of field responses, potentially limiting the interpretation of results. There is also the additional factor that indoor controlled experiments, due to size limitations, are limited to the analysis of juvenile trees. This limits analysis of many traits associated with maturity, such as reproductive development. In order to determine the biological relevance of many responses identified from indoor controlled experiments, it is therefore crucial to also perform outdoor validation experiments to get a completer and more complex overview of the molecular and physiological mechanisms which regulate tree biology.

### Possible limitations of this data set

When interpreting data from this data set it is important to keep a few possible limitations in mind. First, there is the issue of mapping transcriptomes from different genotypes to a reference genome. The outdoor samples are collected from *P. tremula* trees that we treat as individuals, although we cannot exclude that the adult trees are clonal because they are growing in the same area. The indoor samples come from the *Populus tremula × tremuloides* hybrid T89. *P. tremula* and *P. tremuloides* are two very closely related species (Wang et al. 2016) and the coding sequences between the two species only differ with three to six SNPs/1,000 bp. This small difference is not affecting the relative mapping efficiencies to the different genotypes in this study (ranging between 83.6% and 83.7%). However, as with all reference genome-based transcriptomics that is extended to “nonreference” plants (irrespective of if they are different *tremula*, *tremuloides* or hybrid trees) there is always a risk that there are presence/absence parts of the genomes that will not be covered or that some regions of haplotypes are dominant over others.

Taken together, our analysis identifies the most significant co-expression patterns of genes active during different stages of the annual growth cycle in aspen trees. Our findings align with and significantly extends research in other tree species, including conifers (Nose et al. 2020). This analysis highlights the importance of genes already known to be active in the regulation of developmental transitions, in the regulation of cell proliferation and differentiation as well as in the responses to environmental conditions, as discussed in the sections for each seasonal module. However, it also opens for studies of genes of unknown function significantly co-expressed with these genes and genes with functions not previously associated with seasonal changes in trees. The comparison to the experimental data from controlled environments makes it possible to single out effects of daylength and temperature but also highlights the more complex responses found in outside-grown trees that are missed in controlled environments, such as responses to a strongly fluctuating environment including more extreme abiotic conditions and responses to pathogens. We believe this dataset will be an important tool for future design and interpretation of data from both controlled and outdoor experiments.

## Materials and methods

### Plant material and growth conditions

Samples in controlled conditions were collected from hybrid aspen (*Populus tremula* × *Populus tremuloides*), clone T89 (Nilsson et al. 1992) (Supplementary Data Set 1). Plants were cultivated and grown in the same conditions as described in André et al. (2022). After being transferred to soil, the plants were first grown in growth chambers in long-day conditions (LD, 18 h light, 20 °C/6 h dark, 18 °C) before being moved to SDs to induce growth cessation for up to 15 wk (14 h light, 20 °C/10 h dark, 18 °C). A 10-wk CT was later applied to induce dormancy release (8 h light, 4 °C/16 h dark, 6 °C). Ultimately, plants were moved back to the warmer LD conditions to induce bud break. Illumination in the chambers was provided by “Powerstar” lamps giving an R/FR ratio of 2.9 and a light intensity of 150 to 200 mmol m^−2^ s^−1^. The outdoor samples were collected from a local (Umeå, Sweden) 1-yr-old and two 35-yr-old European aspen trees once a month around midday (May to August leaves, September to April apical [vegetative] buds, with no sampling taking place in November). In Umeå, floral buds flush in April but were not included in this analysis. For each plant, for both indoor and outdoor samples, at least three biological replicas were collected at each timepoint consisting of a fully expanded leaf or a pool of three to four buds.

### RNA extraction and sequencing

RNA extraction and sequencing were performed as in André et al. (2022). One hundred milligrams of ground sample was used for RNA extraction with CTAB extraction buffer. The samples were incubated at 65 °C and extracted with chloroform–isoamyl alcohol (24:1). LiCl (10 m) was used to precipitate nucleic acids. Precipitate was collected and purified with RNeasy kit (Qiagen). DNase treatment was performed on-column (Qiagen). Qubit RNA BR Assay Kit (Invitrogen) and Bioanalyzer (Agilent) were used, respectively, to assess concentration and quality of RNA. Three milligrams of total RNA with RIN S8 was sent for sequencing to SciLife Lab, Stockholm. Libraries were prepared following manufacturer's instructions using an Agilent NGS Bravo workstation with TruSeq Stranded mRNA kit (Illumina). Adapter-ligated libraries quality and concentration were assessed on the LabChip GX/HT DNA high sensitivity kit and by Quant-iT, respectively. The libraries were sequenced using the Illumina NovaSeq-6000 platform, generating from 20 to 110 million paired-end reads (2 × 150 bp) per sample. For three timepoints of the outdoor samples, only two out of three replicas passed quality control (Jan, Jun, and Aug).

### Preprocessing of RNA-Seq data

The data preprocessing was performed as described in André et al. (2022) following the guidelines in https://github.com/UPSCb/UPSCb/blob/master/doc/Guidelines-for-RNA-Seq-data-analysis.pdf [DOI:10.5281/zenodo.14002852]. Briefly, FastQC was used to check the quality of raw sequence data, Residual ribosomal RNA (rRNA) contamination was filtered using SortMeRNA. Trimmomatic was employed to remove adapters and trimmed for quality (settings: TruSeq3-PE-2.fa:2:30:10 SLIDINGWINDOW:5:20 MINLEN:50). Filtered reads were pseudo-aligned to v2.2 of the *P. tremula* transcripts (retrieved from the PopGenIE resource) using salmon against an index containing the *P. tremula* v2.2 genome sequence as decoy. All functions were run with the same option settings as in André et al. (2022). In the repository (https://github.com/nicolasDelhomme/aspen-FT-compendium) [DOI: 10.5281/zenodo.14503303], a technical overview of the data, in the form of a MultiQC report, including raw and post-QC read counts and alignment rates is also available.

Data analysis was done in R (v4.4.0) (R Core Team 2024). For quality assessment and visualization, read counts were normalized using the variance stabilizing transformation (VST) function from the package DESeq2 (v.1.44.0) (Love et al. 2014), and only genes with at least 10 mapped reads were analyzed further. The expression data was initially assessed by PCA using the function *prcomp* with default parameters. Hierarchical clustering (*hclust* function) was used to cluster genes (Pearson correlation) and samples (ward.D2 distance). All heatmaps were rendered with *pheatmap* and scaled by rows.

### Co-expression analysis

For the co-expression network analysis, the package Weighted Gene Co-expression Network Analysis (WGCNA) was used (v. 1.72.5) (Langfelder and Horvath 2008, 2012). Network modules were found using the *blockwiseModules* function with the following parameters: power = 10 (scale-free topology fit with an R^2^ of 0.8), maxBlockSize = 10,000, networkType = “signed”, TOMType = “signed”, corType = “bicor”, maxPOutliers = 0.05, replaceMissingAdjacencies = TRUE, pamStage = F, deepSplit = 1, minModuleSize = 5, minKMEtoStay = 0.3, minCoreKME = 0.3, minCoreKMESize = 2, reassignThreshold = 0, and mergeCutHeight = 0.2. To visualize the networks Cytoscape v. 3.10.1 was used.

### Differential expression and GO analysis

Differential expression analysis was done using the package DESeq2 (v.1.44.0) (Love et al. 2014) and genes with an adjusted *P*-value less than 0.05 (Benjamini–Hochberg method) and a log2 FC equal or greater than 1 were retained.

For GO analysis, the function *hyperGTest* from the GSEABase package was used (v. 1.66.0) (Morgan et al. 2024) with the following parameters: ontology = “BP”, pvalueCutoff = 0.05, conditional = FALSE and testDirection = “over”.

All the custom R scripts are available on (https://github.com/nicolasDelhomme/aspen-FT-compendium) [DOI: 10.5281/zenodo.14503303].

### POPUL-R—a shiny app to visualize seasonal patterns of gene expression in *Populus* trees

The data generated with co-expression analysis, describe some of the main processes characterizing the annual growth cycle of *Populus* trees. To make the data more accessible and provide an open resource for the scientific community to use we created an R package “Shiny” application called “POPUL-R”.

POPUL-R allows users to select one or several genes and plot their transcription profiles throughout the yearly growth cycle in aspen. For a few selected genes, it is possible to identify their first neighbors from the co-expression data and to create an interactive visualization of the network. In addition, the generated graphs and tables can be easily exported in different formats.

The first tab of POPUL-R, “Introduction”, provides a brief description of the application and how to navigate the different tabs. A tutorial video is included for step-by-step guidance. The “Materials and methods” tab outlines the trees growth conditions, and the packages used to analyze and visualize the data. The main two tabs, “Gene profile” and “Module profile”, both contain an “Instructions” section, to assist the user in plotting the intended data. “Gene profile” allows the visualization of the expression profile of one or multiple genes in form of a line plot and a heatmap. In addition, the edge weight of the selected genes within the co-expression network is displayed as well. The users can therefore use this information to set a weight threshold and obtain a table of the selected genes and their first neighbors, to later visualize them in an interactive co-expression network. “Module profile” shows a heatmap of the genes included in the selected module and provides a table of the enriched GO terms. From this table the user can select multiple GOs and get a list of genes specifically associated to them.

The “References and citations” tab displays tools and references for the published datasets used in POPUL-R and provides guidelines to cite the app.

POPUL-R was built in R (v4.4.0, R Core Team 2024) with the package Shiny v 1.9.1 (Chang et al. 2024) and tested on Safari 17.6 on Mac OS Ventura. Heatmaps were generated with the R packages ComplexHeatmap v2.20.0 and Interactive Complex Heatmap v.1.12.0 (Gu et al. 2016; Gu 2022; Gu and Hubschmann 2022). Tables with first neighbors were generated using the R package DT v0.33 (Xie et al. 2025), and interactive networks were built with the R package networkD3 v0.4 (Allaire et al. 2017). The remaining plots were generated using the R package ggplot2 v3.5.1 (Wickham and Wickham 2016). Custom R scripts can be found on: (https://github.com/lauragarciaromanach/POPUL_R).

The multiple features, make POPUL-R a very valuable tool for the scientific community to explore and validate the role of specific genes during the annual growth cycle of aspen trees. POPUL-R can be accessed at: https://lauragarciaromanach.shinyapps.io/popul_r_mini/

### Accession numbers

The raw data is available from the European Nucleotide Archive (ENA: https://ebi.ac.uk/ena) under the accession number PRJEB81972. *P. tremula* and *A. thaliana* gene identifiers for genes referenced in this article can be found in Supplementary Data Set 7.

## Supplementary Material

koaf208_Supplementary_Data

## Data Availability

The data underlying this article are available in the article and in its online supplementary material.

## Supplementary data

The following materials are available in the online version of this article.

**Supplementary Figure S1.** Heatmap of all outdoor samples for each month.

**Supplementary Data Set 1.** List of all 207 samples specifying age (Y, young; A, adult), tissue, treatment/month and location.

**Supplementary Data Set 2.** List of genes belonging to each module.

**Supplementary Data Set 3.** Heatmap showing the expression profile of all genes from each module.

**Supplementary Data Set 4.** List of GO terms enriched in each module.

**Supplementary Data Set 5.** List of all DEGs for each contrast.

**Supplementary Data Set 6.** List of GO terms enriched in each contrast.

**Supplementary Data Set 7.** List of genes mentioned in this study.

## Dive Curated Terms

The following phenotypic, genotypic, and functional terms are of significance to the work described in this paper:

CDF2 Gramene: AT5G39660
CDF2 Araport: AT5G39660

SPL Gramene: AT4G27330
SPL Araport: AT4G27330

CDF3 Gramene: AT3G47500
CDF3 Araport: AT3G47500

NF-YA7 Gramene: AT1G30500
NF-YA7 Araport: AT1G30500

AHL Gramene: AT5G54390
AHL Araport: AT5G54390

CIB1 Gramene: AT4G34530
CIB1 Araport: AT4G34530

COL3 Gramene: AT2G24790
COL3 Araport: AT2G24790

PIF4 Gramene: AT2G43010
PIF4 Araport: AT2G43010

TSF Gramene: AT4G20370
TSF Araport: AT4G20370

SPL13B Gramene: At5g50670
SPL13B Araport: At5g50670
