# Supplementary material for: A transcriptional roadmap of the yearly growth cycle in Populus trees
Source: Plant Cell. 2025 Aug 20;37(9):koaf208. doi: 10.1093/plcell/koaf208 (PMC12419529; doi:10.1093/plcell/koaf208)
Supplement: koaf208_Supplementary_Data [file koaf208_supplementary_data.zip › Supplementary Data Set 3.pdf]

Supplementary Data. Macon et al (2025)
Supplementary Data Set 3. Heatmap showing the expression profile of all genes from each module.
From Module 1 (M1) to module 46 (M46).

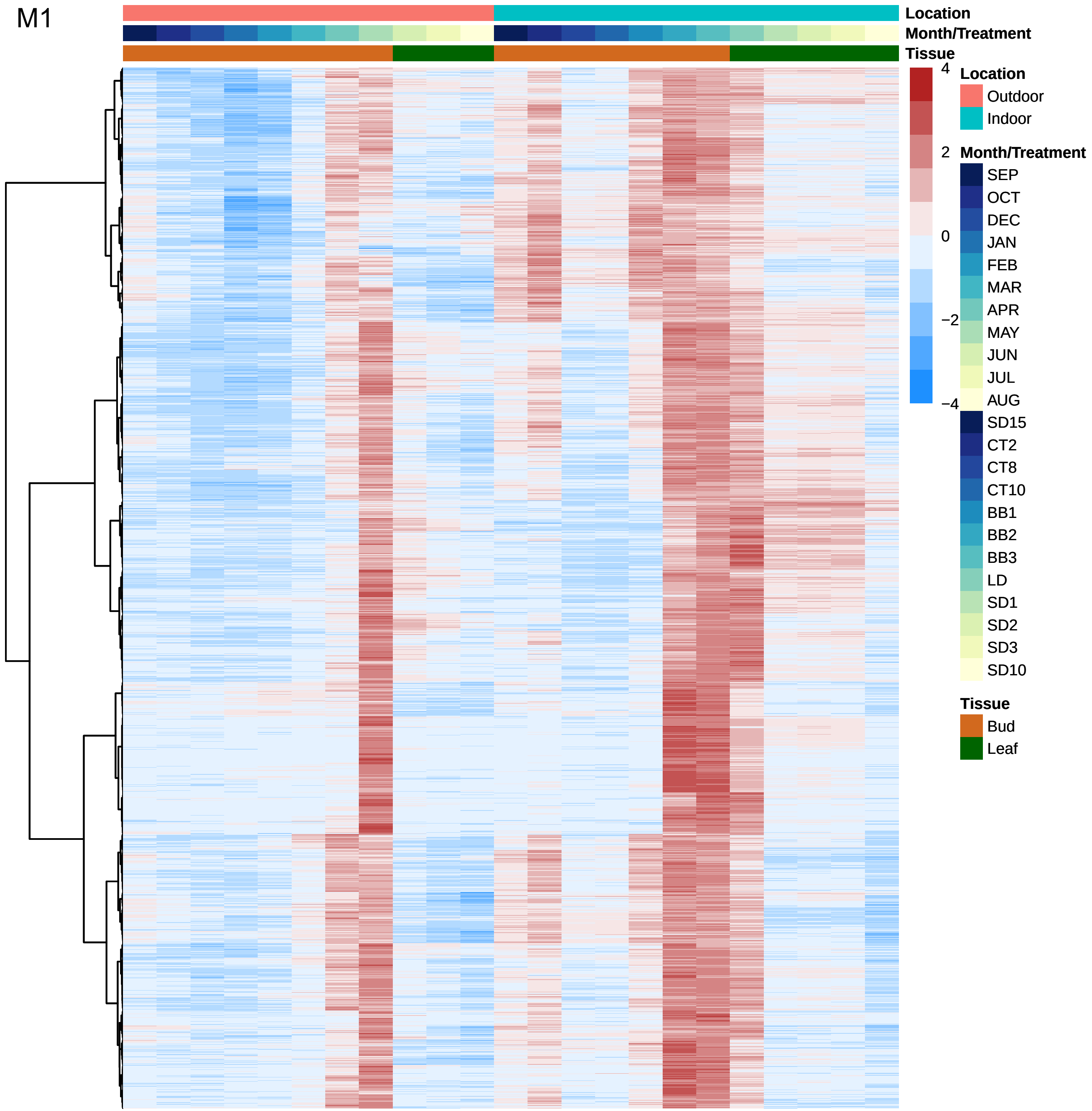
M1
Location
Month/Treatment
Tissue
4
2
0
−2
−4
Location
Outdoor
Indoor
Month/Treatment
SEP
OCT
DEC
JAN
FEB
MAR
APR
MAY
JUN
JUL
AUG
SD15
CT2
CT8
CT10
BB1
BB2
BB3
LD
SD1
SD2
SD3
SD10
Tissue
Bud
Leaf

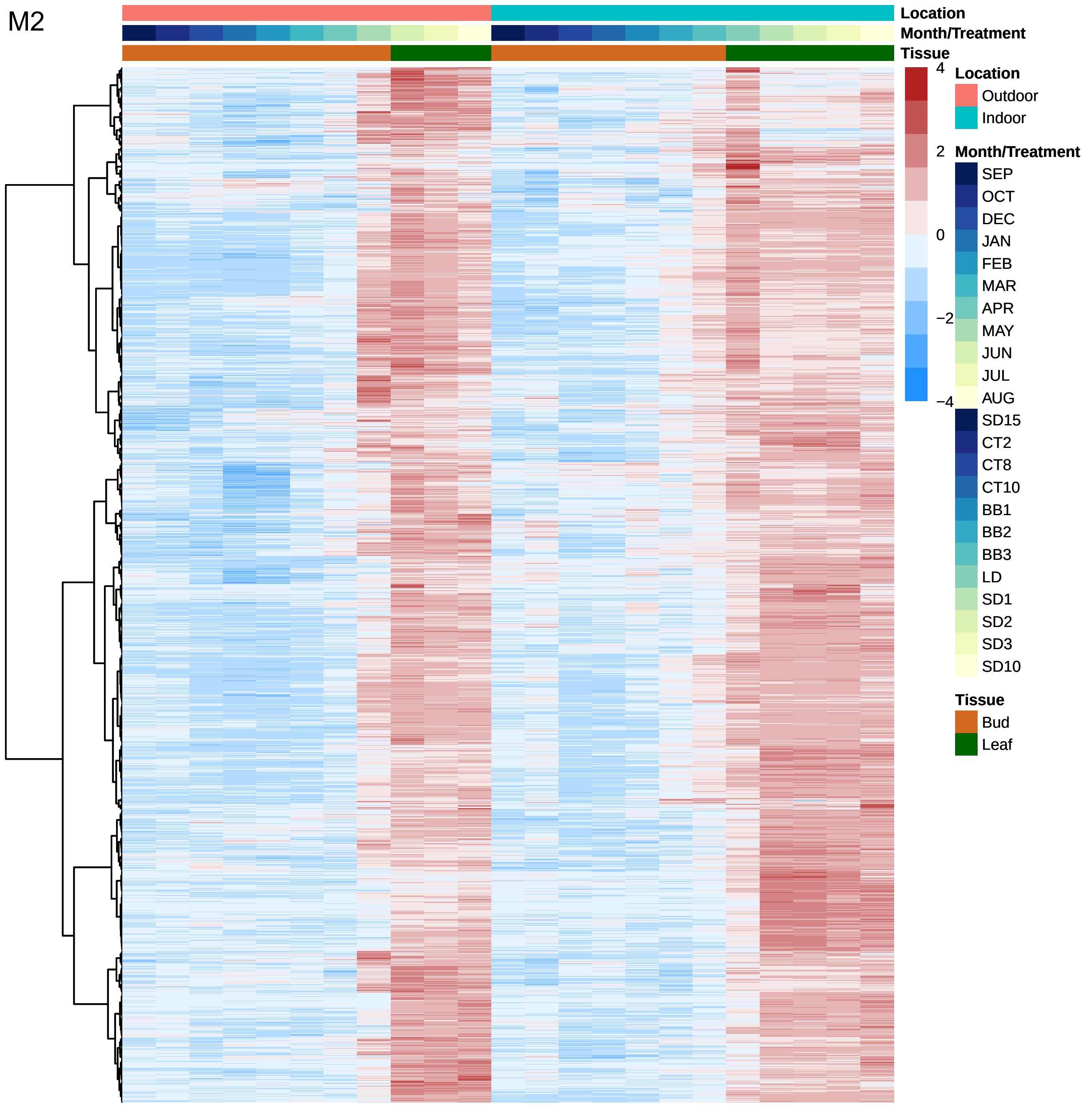
M2
Location
Month/Treatment
Tissue
4
2
0
−2
−4
Location
Outdoor
Indoor
Month/Treatment
SEP
OCT
DEC
JAN
FEB
MAR
APR
MAY
JUN
JUL
AUG
SD15
CT2
CT8
CT10
BB1
BB2
BB3
LD
SD1
SD2
SD3
SD10
Tissue
Bud
Leaf

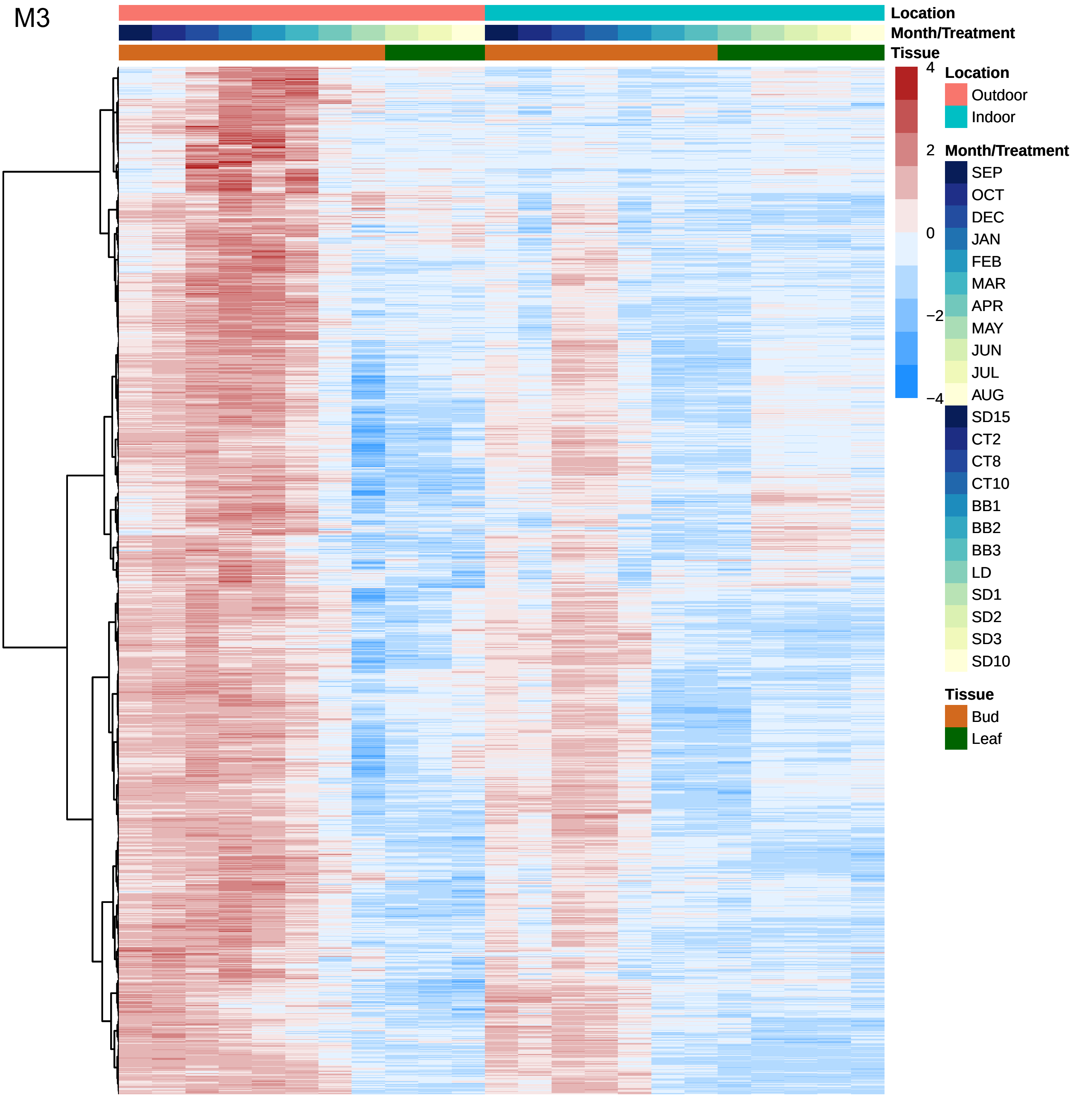
M3
Location
Month/Treatment
Tissue
4
2
0
−2
−4
Location
Outdoor
Indoor
Month/Treatment
SEP
OCT
DEC
JAN
FEB
MAR
APR
MAY
JUN
JUL
AUG
SD15
CT2
CT8
CT10
BB1
BB2
BB3
LD
SD1
SD2
SD3
SD10
Tissue
Bud
Leaf

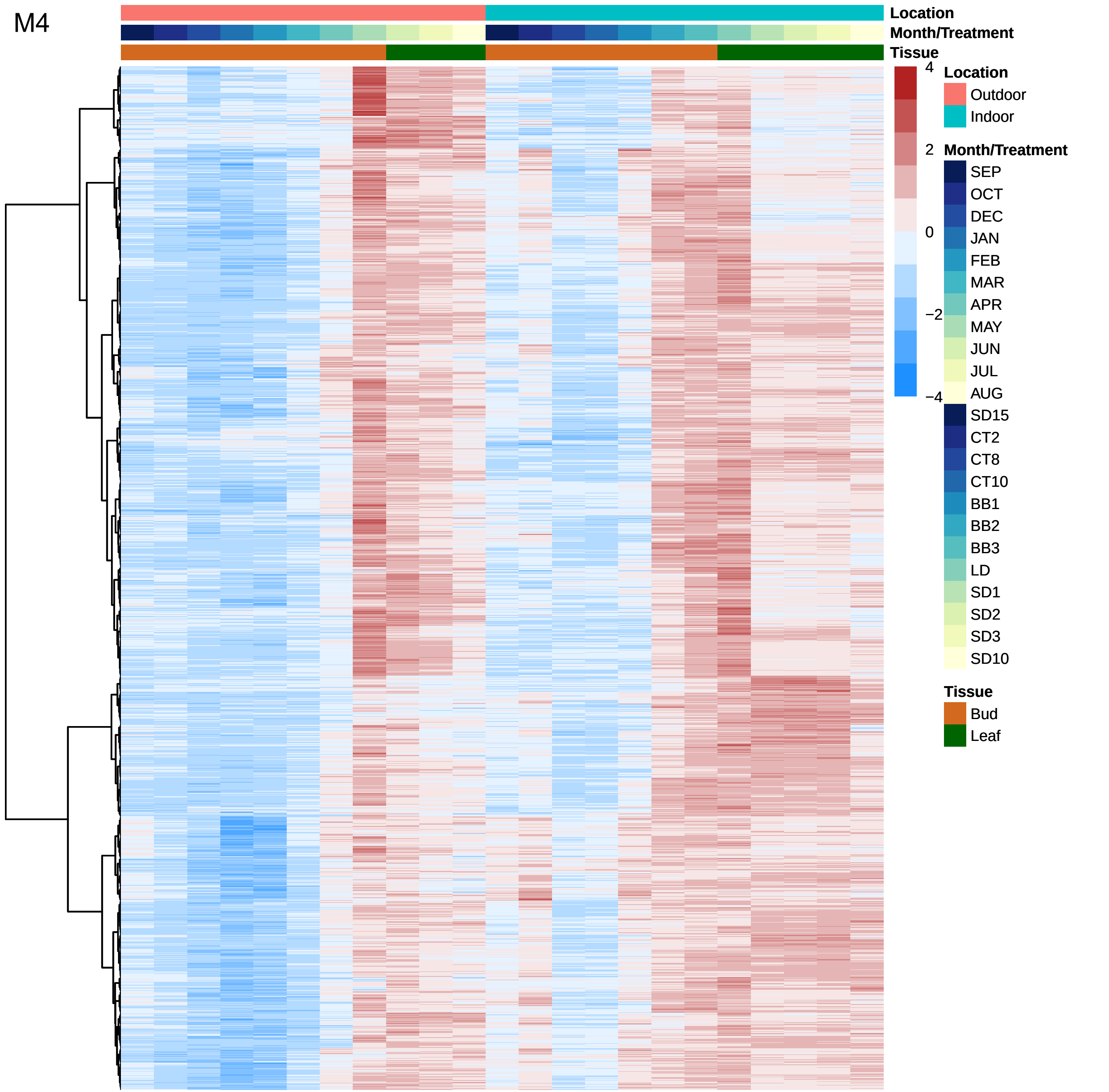
M4
Location
Month/Treatment
Tissue
4
2
0
−2
−4
Location
Outdoor
Indoor
Month/Treatment
SEP
OCT
DEC
JAN
FEB
MAR
APR
MAY
JUN
JUL
AUG
SD15
CT2
CT8
CT10
BB1
BB2
BB3
LD
SD1
SD2
SD3
SD10
Tissue
Bud
Leaf

M5

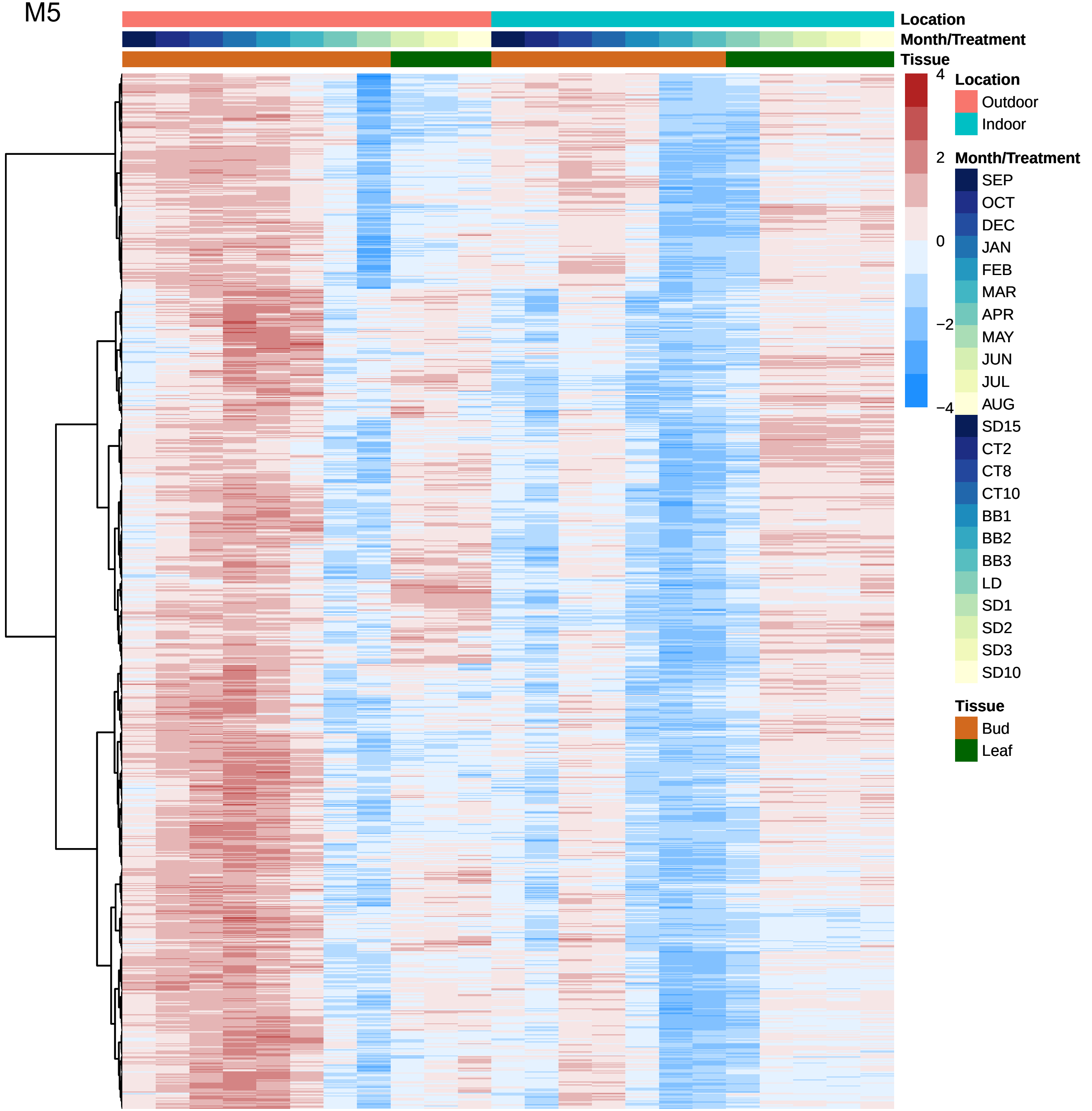
Location
Month/Treatment
Tissue
4
2
0
−2
−4
Location
Outdoor
Indoor
Month/Treatment
SEP
OCT
DEC
JAN
FEB
MAR
APR
MAY
JUN
JUL
AUG
SD15
CT2
CT8
CT10
BB1
BB2
BB3
LD
SD1
SD2
SD3
SD10
Tissue
Bud
Leaf

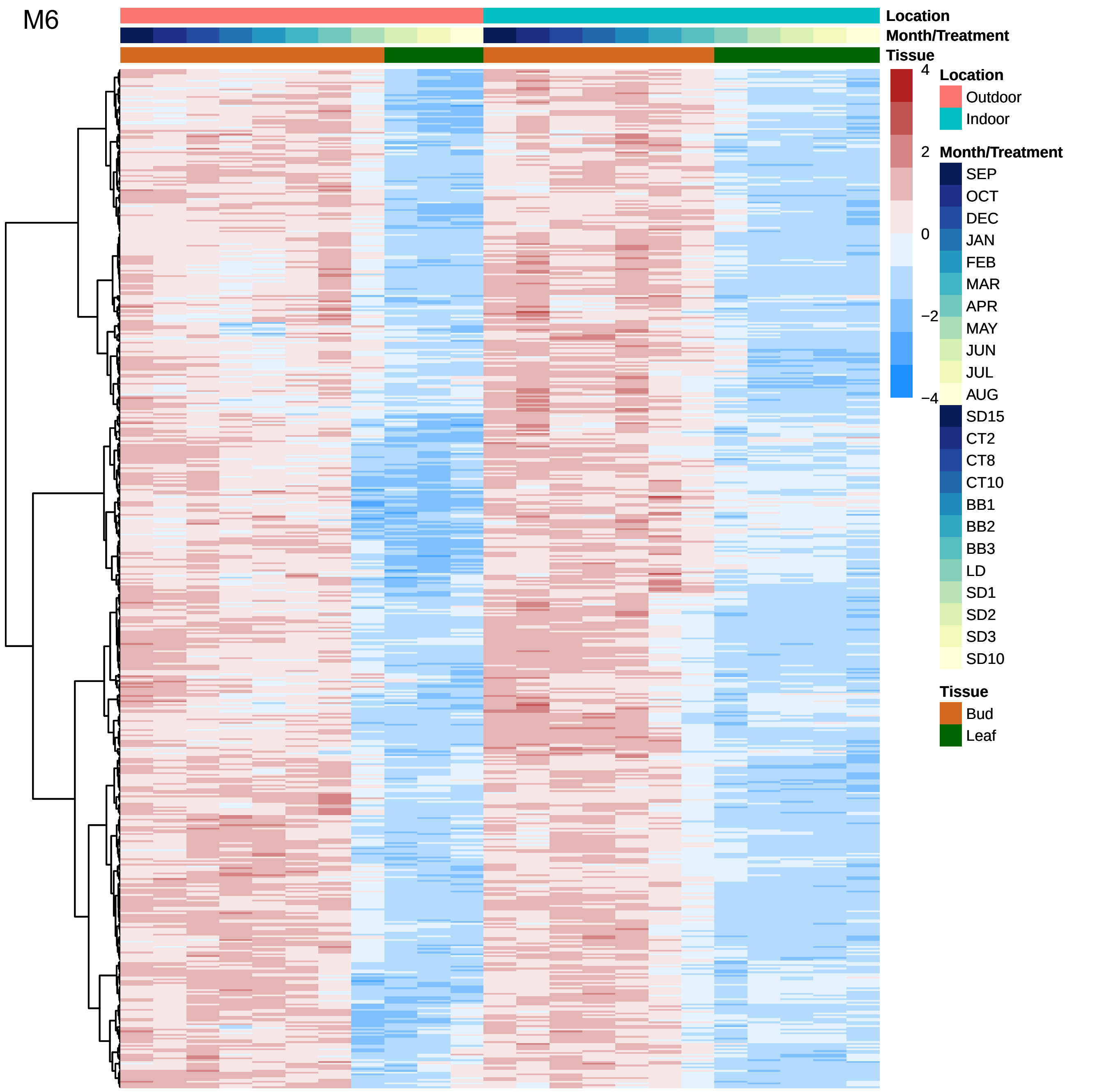
M6
Location
Month/Treatment
Tissue
4
2
0
−2
−4
Location
Outdoor
Indoor
Month/Treatment
SEP
OCT
DEC
JAN
FEB
MAR
APR
MAY
JUN
JUL
AUG
SD15
CT2
CT8
CT10
BB1
BB2
BB3
LD
SD1
SD2
SD3
SD10
Tissue
Bud
Leaf

M7

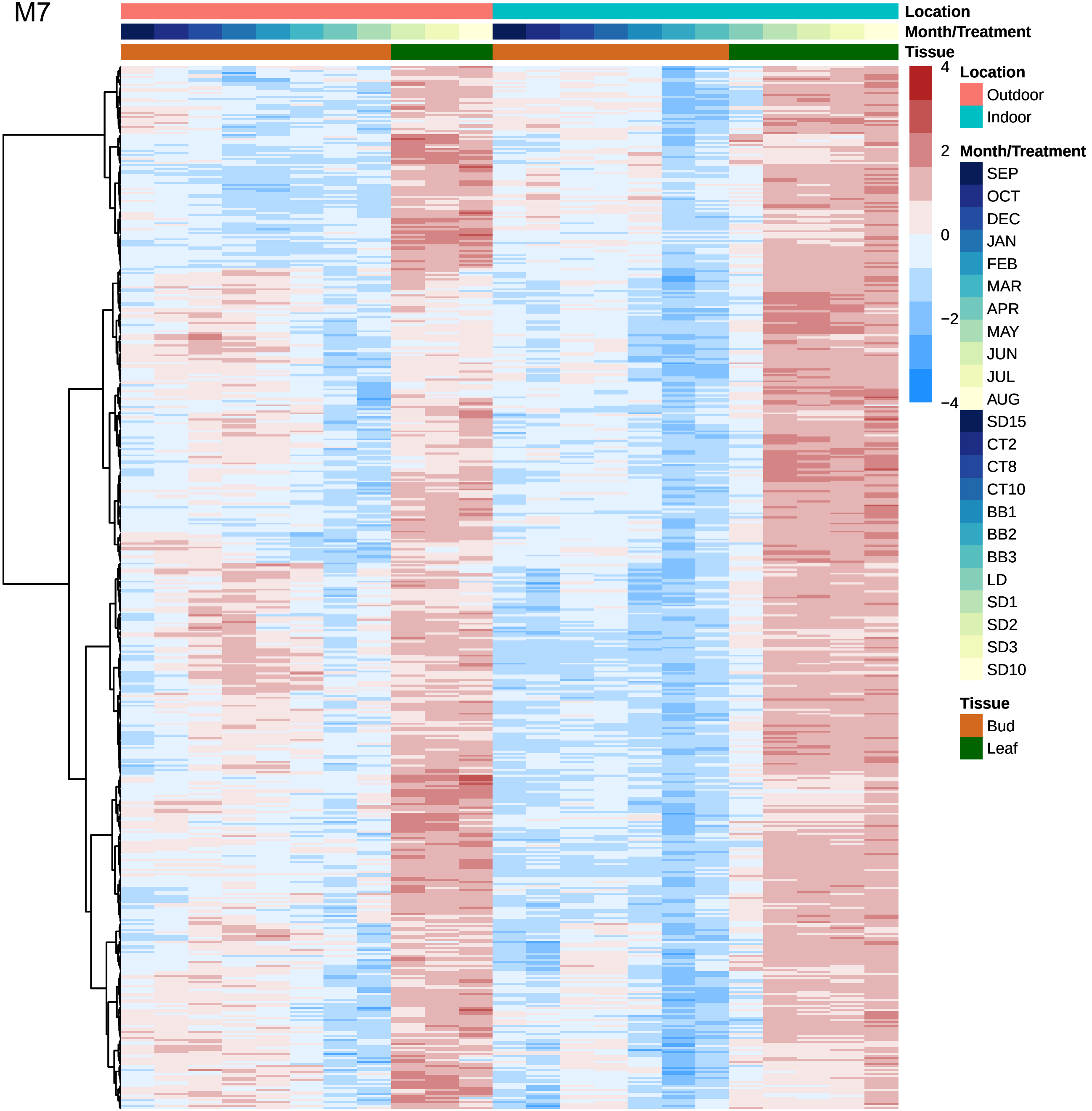
Location
Month/Treatment
Tissue
4
2
0
−2
−4
Location
Outdoor
Indoor
Month/Treatment
SEP
OCT
DEC
JAN
FEB
MAR
APR
MAY
JUN
JUL
AUG
SD15
CT2
CT8
CT10
BB1
BB2
BB3
LD
SD1
SD2
SD3
SD10
Tissue
Bud
Leaf

M8

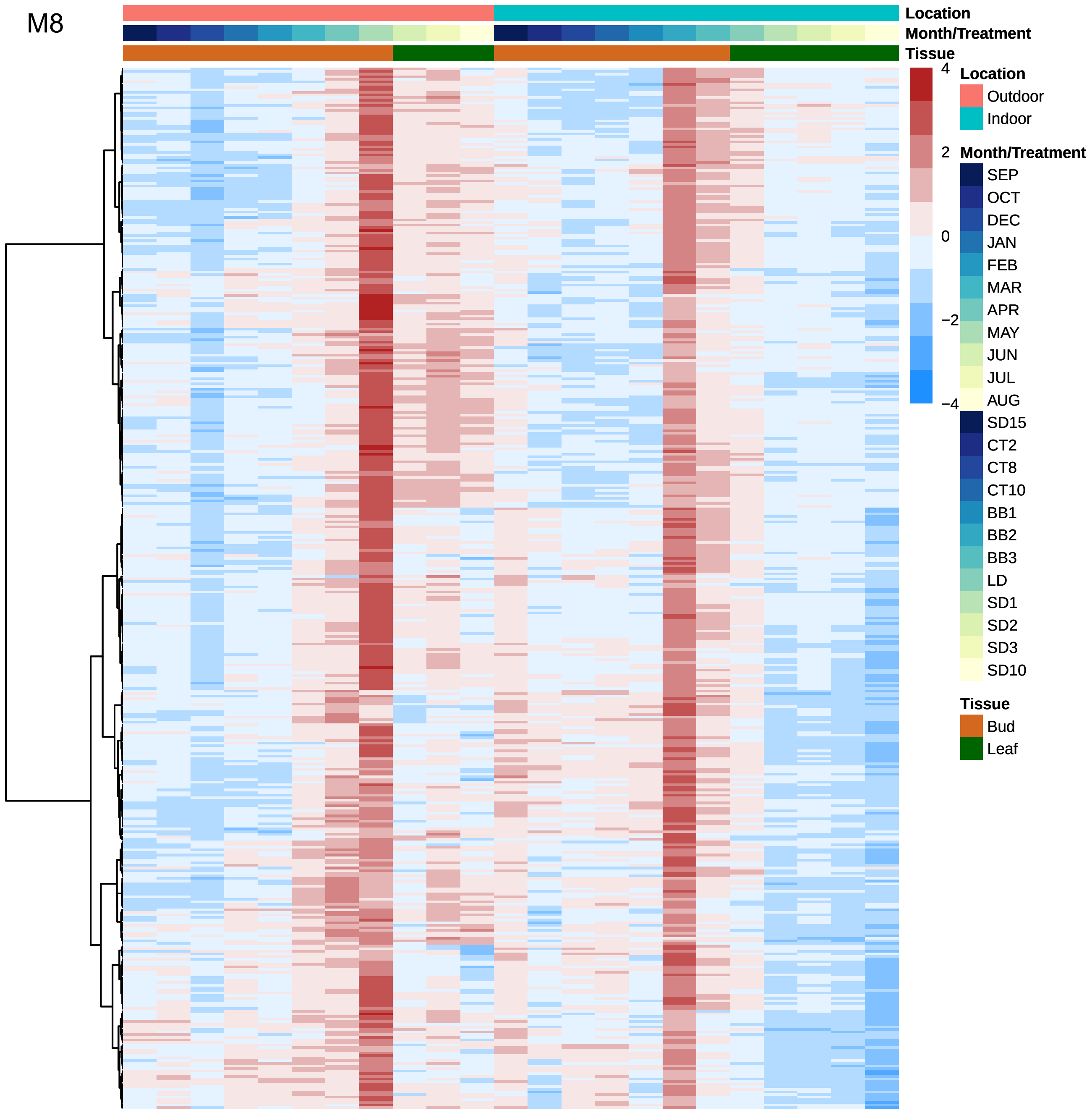

Location
Month/Treatment
Tissue
4
2
0
−2
−4
Location
Outdoor
Indoor
Month/Treatment
SEP
OCT
DEC
JAN
FEB
MAR
APR
MAY
JUN
JUL
AUG
SD15
CT2
CT8
CT10
BB1
BB2
BB3
LD
SD1
SD2
SD3
SD10
Tissue
Bud
Leaf

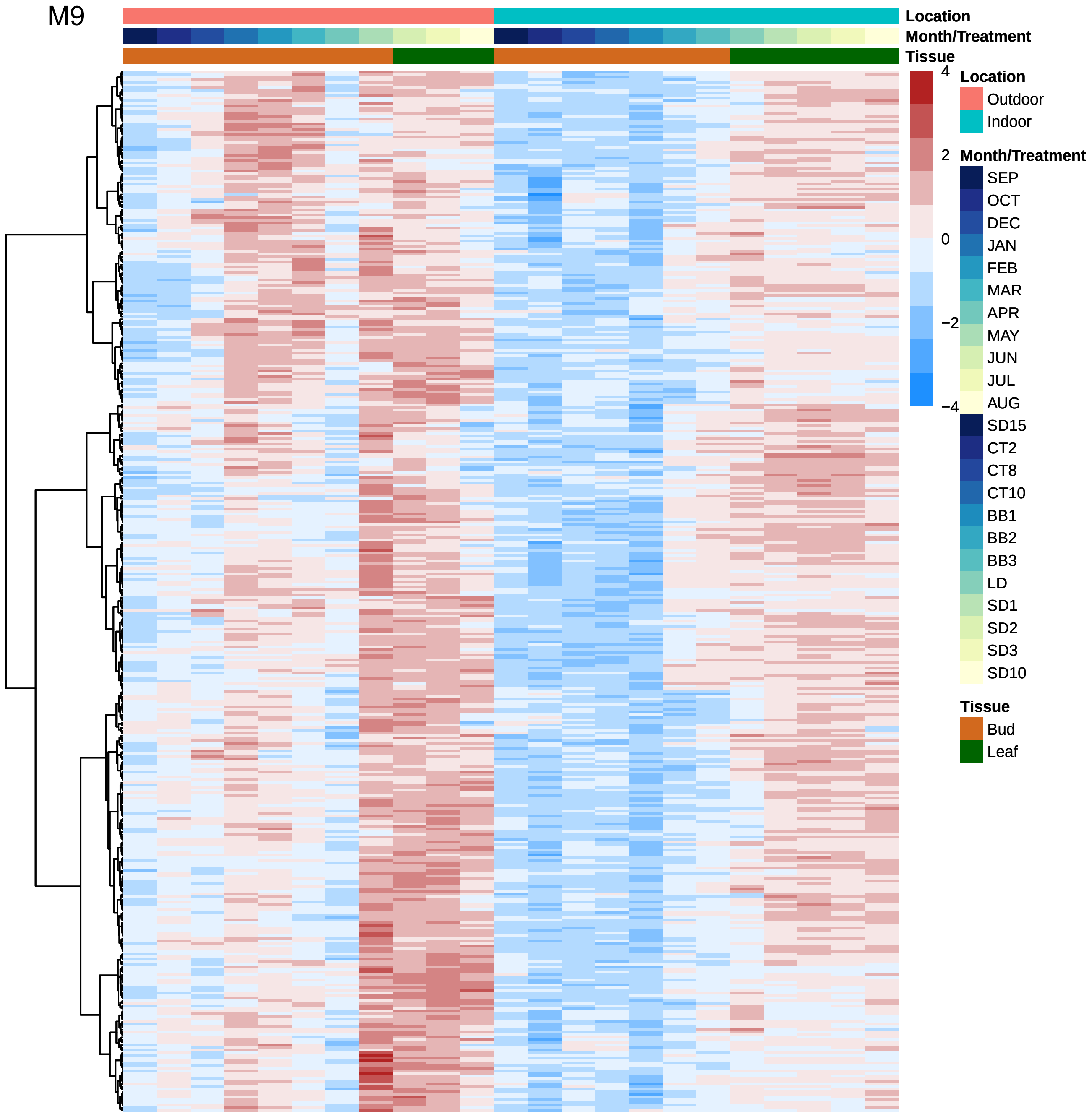
M9
Location
Month/Treatment
Tissue
4
2
0
−2
−4
Location
Outdoor
Indoor
Month/Treatment
SEP
OCT
DEC
JAN
FEB
MAR
APR
MAY
JUN
JUL
AUG
SD15
CT2
CT8
CT10
BB1
BB2
BB3
LD
SD1
SD2
SD3
SD10
Tissue
Bud
Leaf

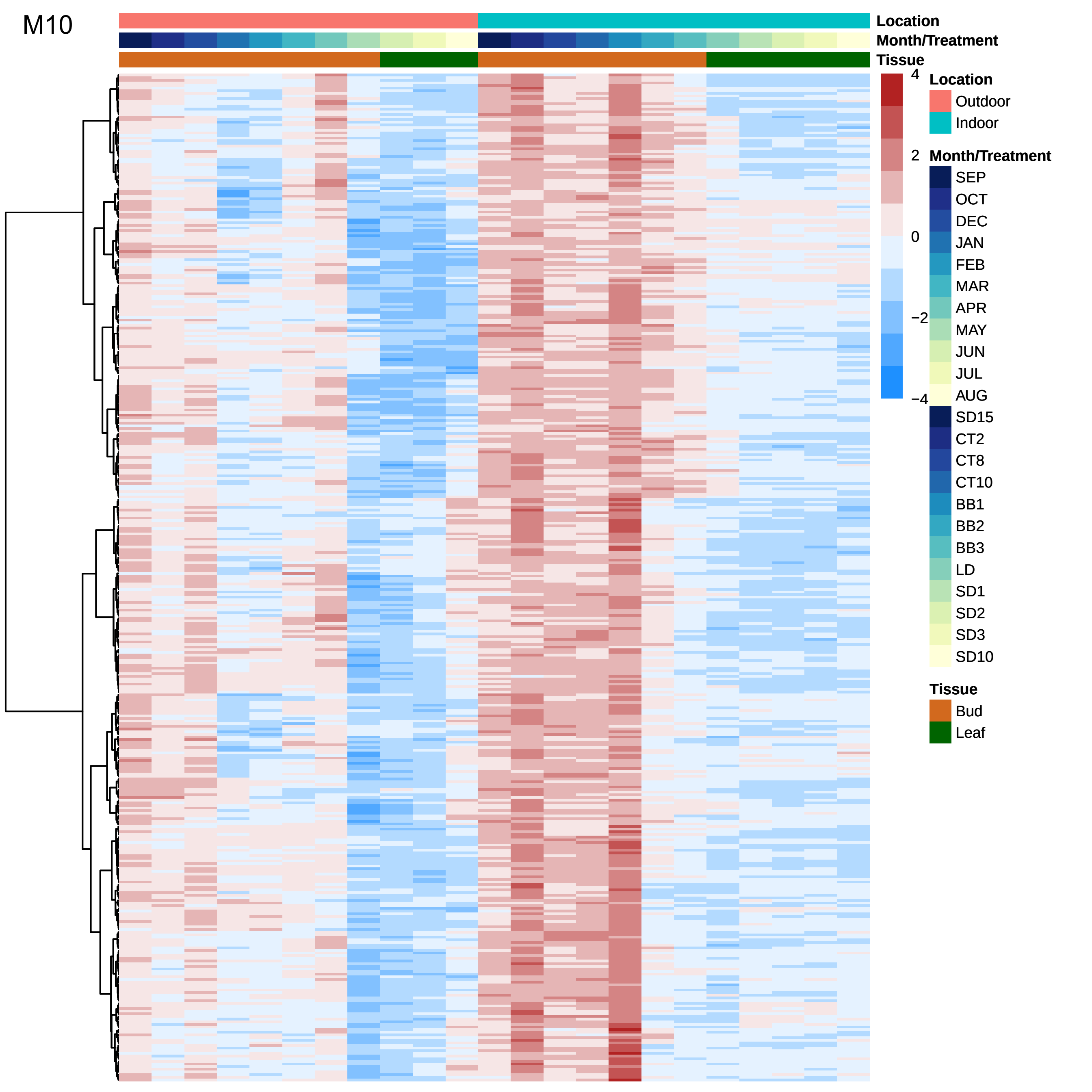
M10
Location
Month/Treatment
Tissue
4
2
0
−2
−4
Location
Outdoor
Indoor
Month/Treatment
SEP
OCT
DEC
JAN
FEB
MAR
APR
MAY
JUN
JUL
AUG
SD15
CT2
CT8
CT10
BB1
BB2
BB3
LD
SD1
SD2
SD3
SD10
Tissue
Bud
Leaf

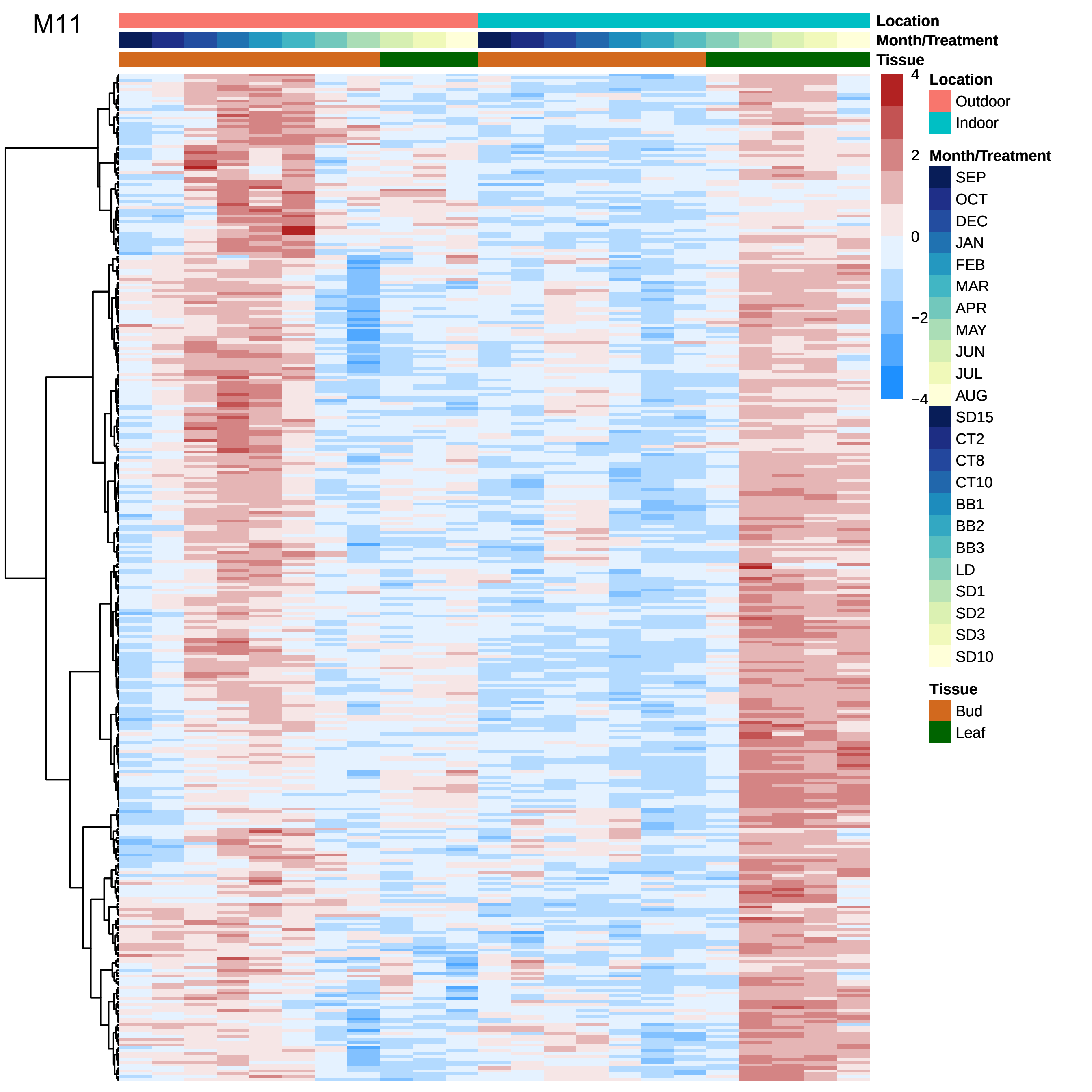
M11
Location
Month/Treatment
Tissue
4
2
0
−2
−4
Location
Outdoor
Indoor
Month/Treatment
SEP
OCT
DEC
JAN
FEB
MAR
APR
MAY
JUN
JUL
AUG
SD15
CT2
CT8
CT10
BB1
BB2
BB3
LD
SD1
SD2
SD3
SD10
Tissue
Bud
Leaf

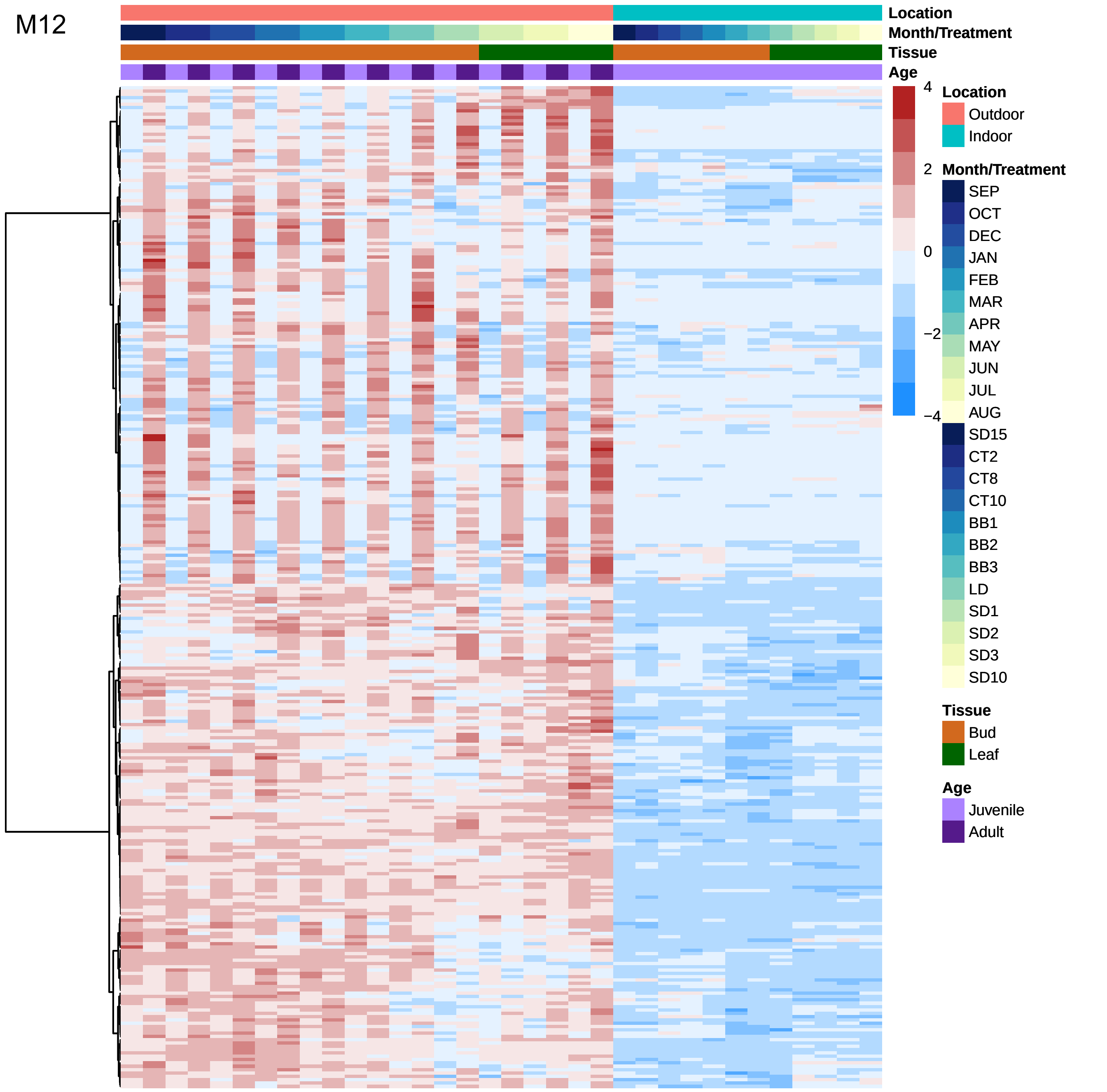
M12
Location
Month/Treatment
Tissue
Age
4
2
0
−2
−4
Location
Outdoor
Indoor
Month/Treatment
SEP
OCT
DEC
JAN
FEB
MAR
APR
MAY
JUN
JUL
AUG
SD15
CT2
CT8
CT10
BB1
BB2
BB3
LD
SD1
SD2
SD3
SD10
Tissue
Bud
Leaf
Age
Juvenile
Adult

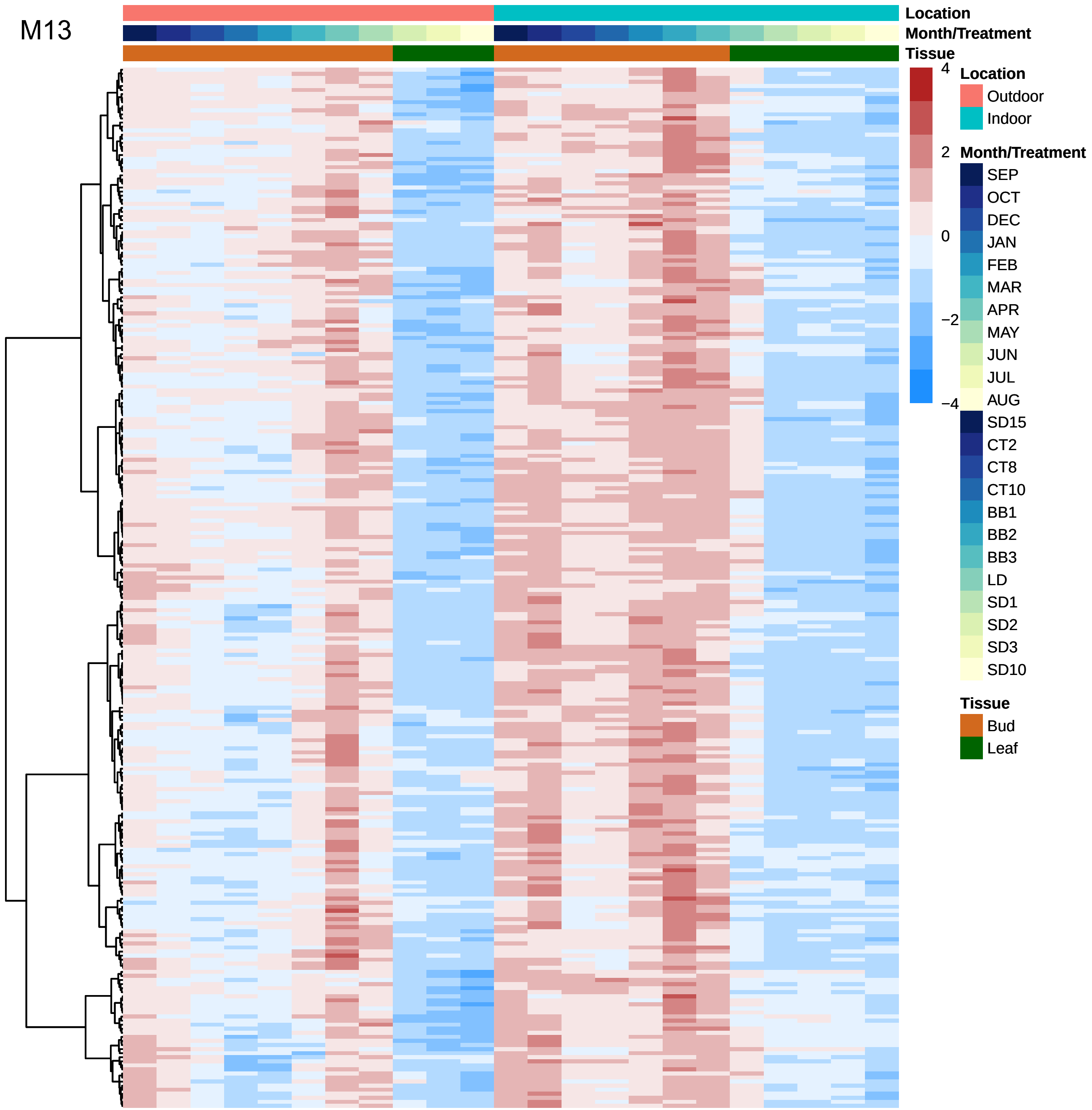
M13
Location
Month/Treatment
Tissue
4
2
0
−2
−4
Location
Outdoor
Indoor
Month/Treatment
SEP
OCT
DEC
JAN
FEB
MAR
APR
MAY
JUN
JUL
AUG
SD15
CT2
CT8
CT10
BB1
BB2
BB3
LD
SD1
SD2
SD3
SD10
Tissue
Bud
Leaf

M14

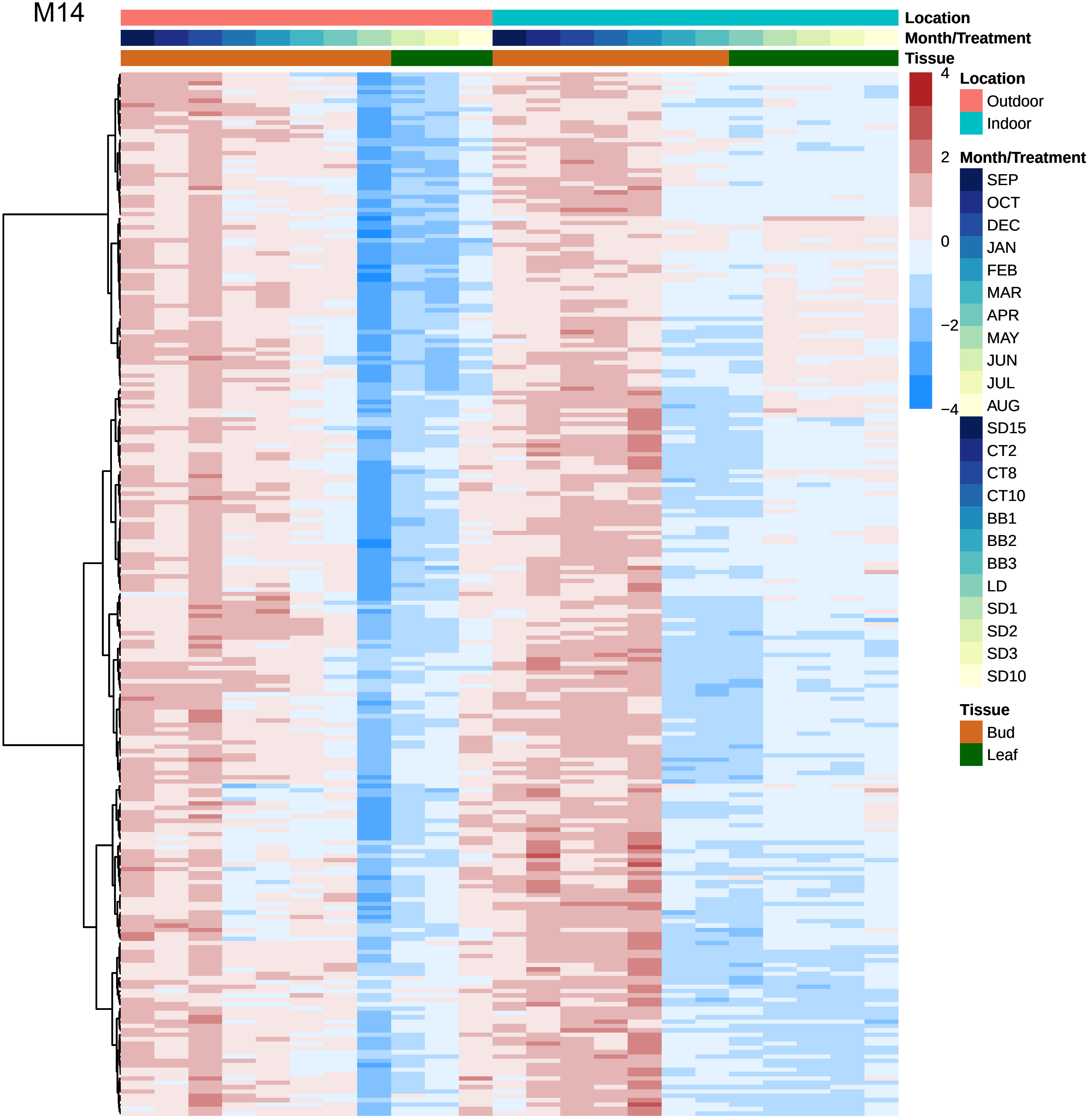
Location
Month/Treatment
Tissue
4
2
0
−2
−4
Location
Outdoor
Indoor
Month/Treatment
SEP
OCT
DEC
JAN
FEB
MAR
APR
MAY
JUN
JUL
AUG
SD15
CT2
CT8
CT10
BB1
BB2
BB3
LD
SD1
SD2
SD3
SD10
Tissue
Bud
Leaf

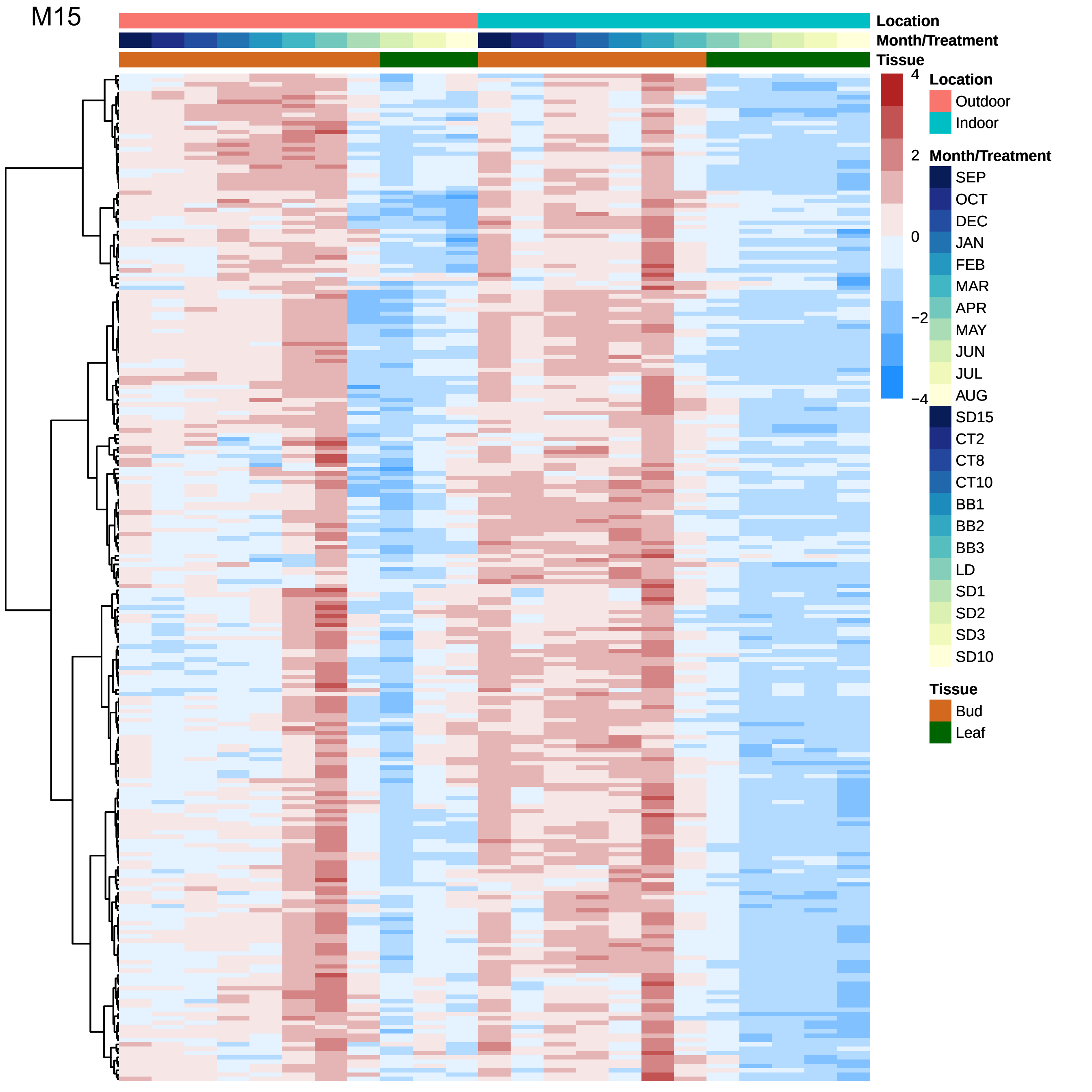

M15
Location
Month/Treatment
Tissue
4
2
0
−2
−4
Location
Outdoor
Indoor
Month/Treatment
SEP
OCT
DEC
JAN
FEB
MAR
APR
MAY
JUN
JUL
AUG
SD15
CT2
CT8
CT10
BB1
BB2
BB3
LD
SD1
SD2
SD3
SD10
Tissue
Bud
Leaf

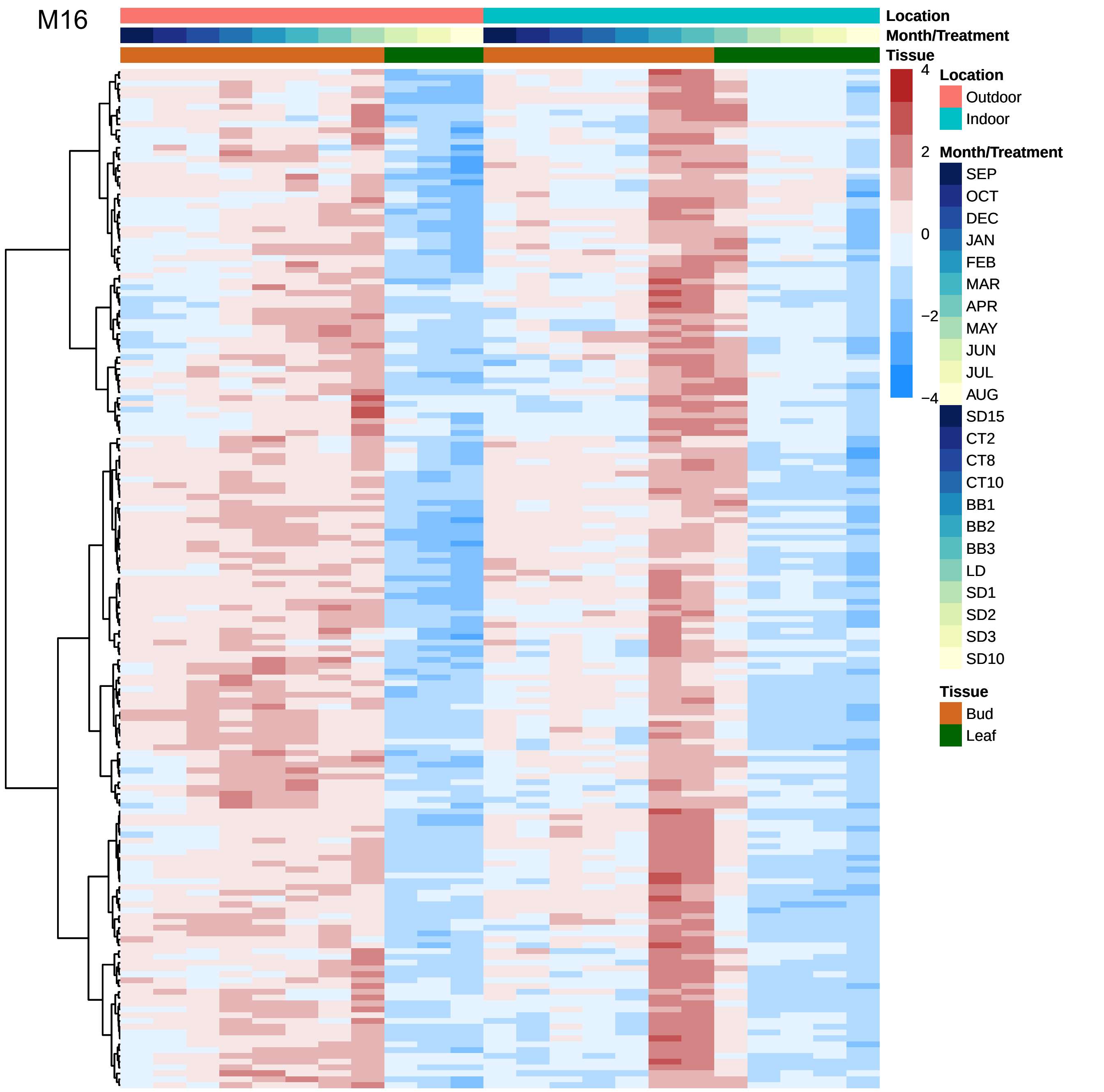
M16
Location
Month/Treatment
Tissue
4
2
0
−2
−4
Location
Outdoor
Indoor
Month/Treatment
SEP
OCT
DEC
JAN
FEB
MAR
APR
MAY
JUN
JUL
AUG
SD15
CT2
CT8
CT10
BB1
BB2
BB3
LD
SD1
SD2
SD3
SD10
Tissue
Bud
Leaf

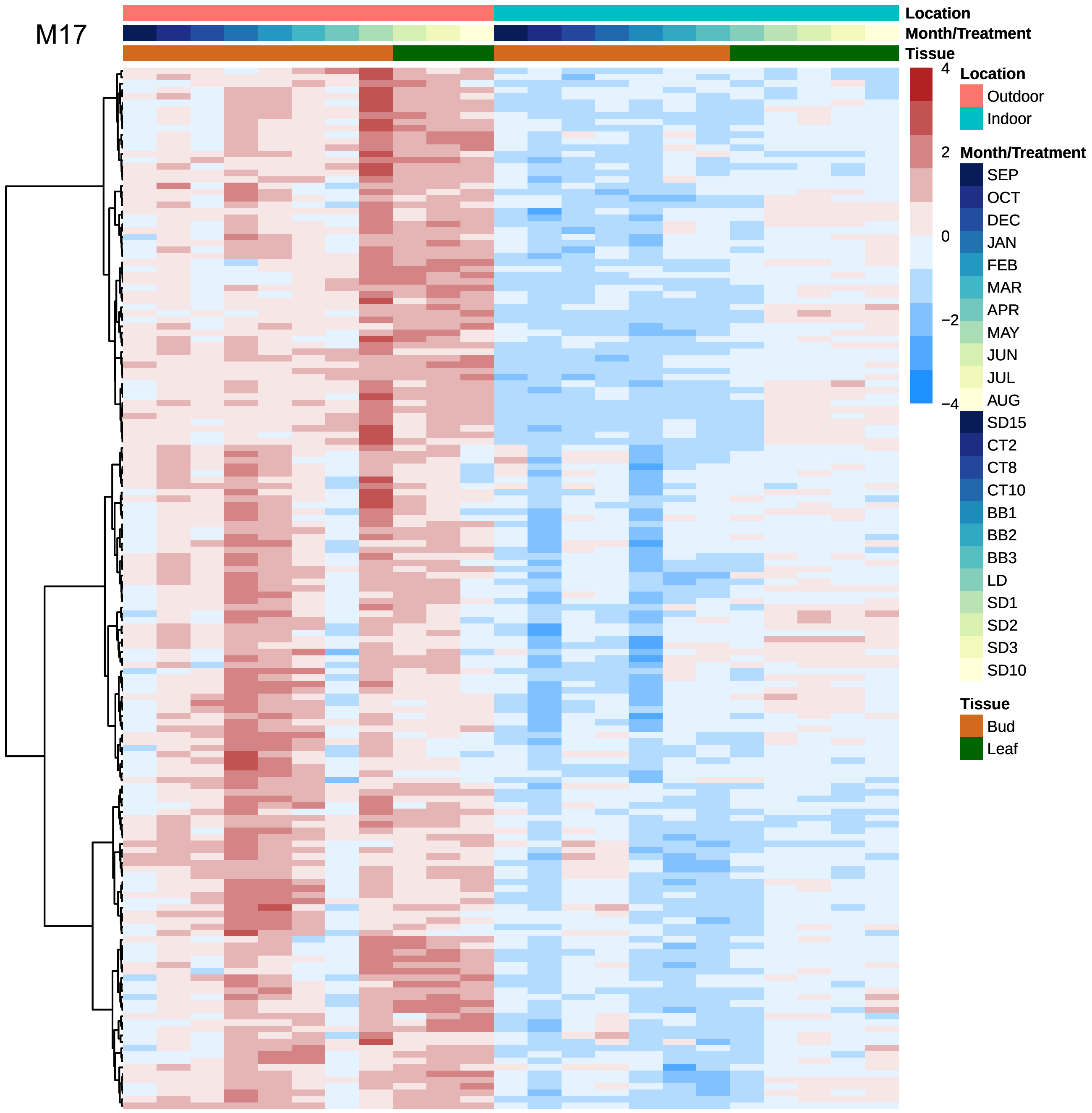
M17
Location
Month/Treatment
Tissue
4
2
0
−2
−4
Location
Outdoor
Indoor
Month/Treatment
SEP
OCT
DEC
JAN
FEB
MAR
APR
MAY
JUN
JUL
AUG
SD15
CT2
CT8
CT10
BB1
BB2
BB3
LD
SD1
SD2
SD3
SD10
Tissue
Bud
Leaf

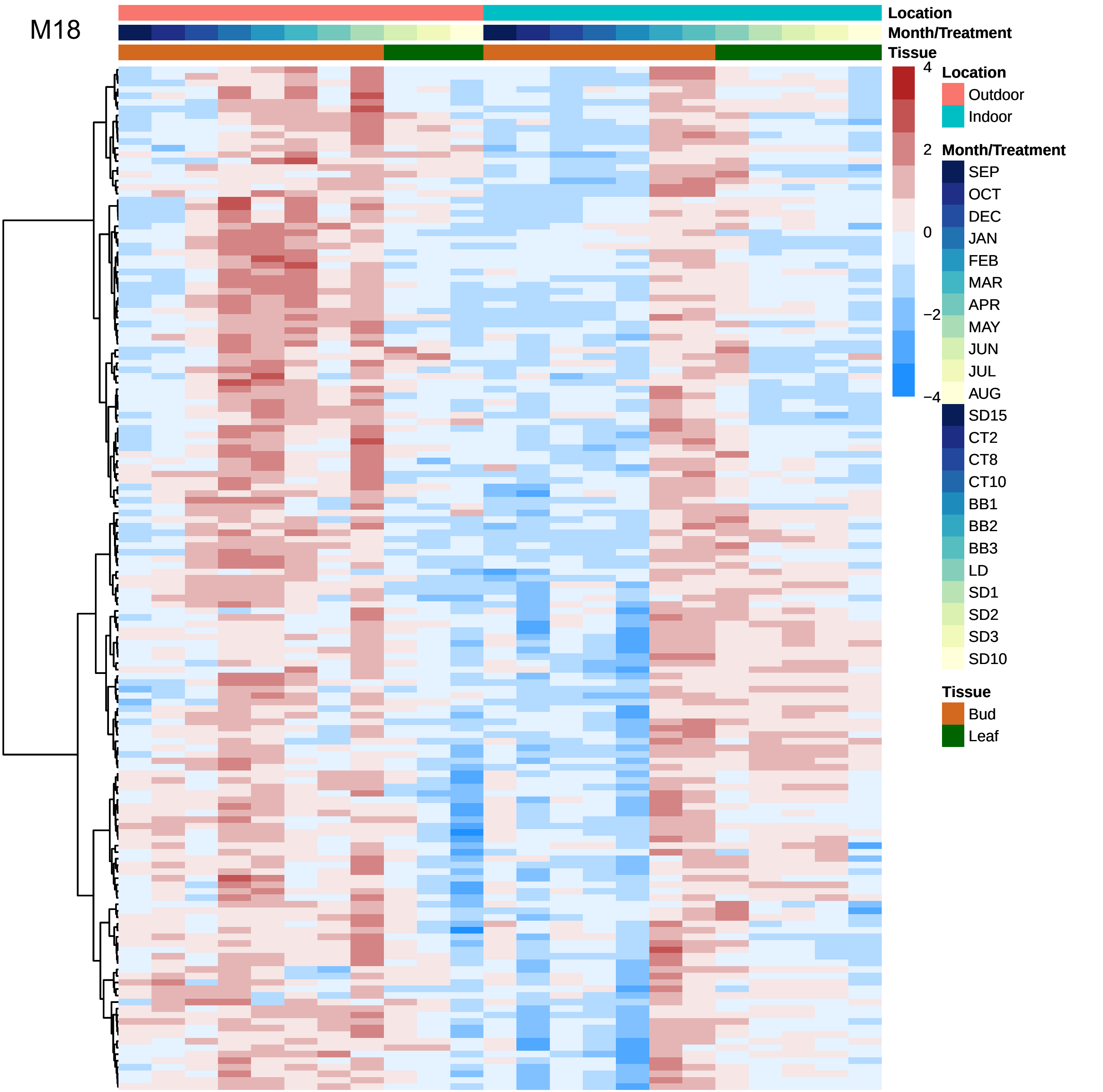

M18
Location
Month/Treatment
Tissue
4
2
0
−2
−4
Location
Outdoor
Indoor
Month/Treatment
SEP
OCT
DEC
JAN
FEB
MAR
APR
MAY
JUN
JUL
AUG
SD15
CT2
CT8
CT10
BB1
BB2
BB3
LD
SD1
SD2
SD3
SD10
Tissue
Bud
Leaf

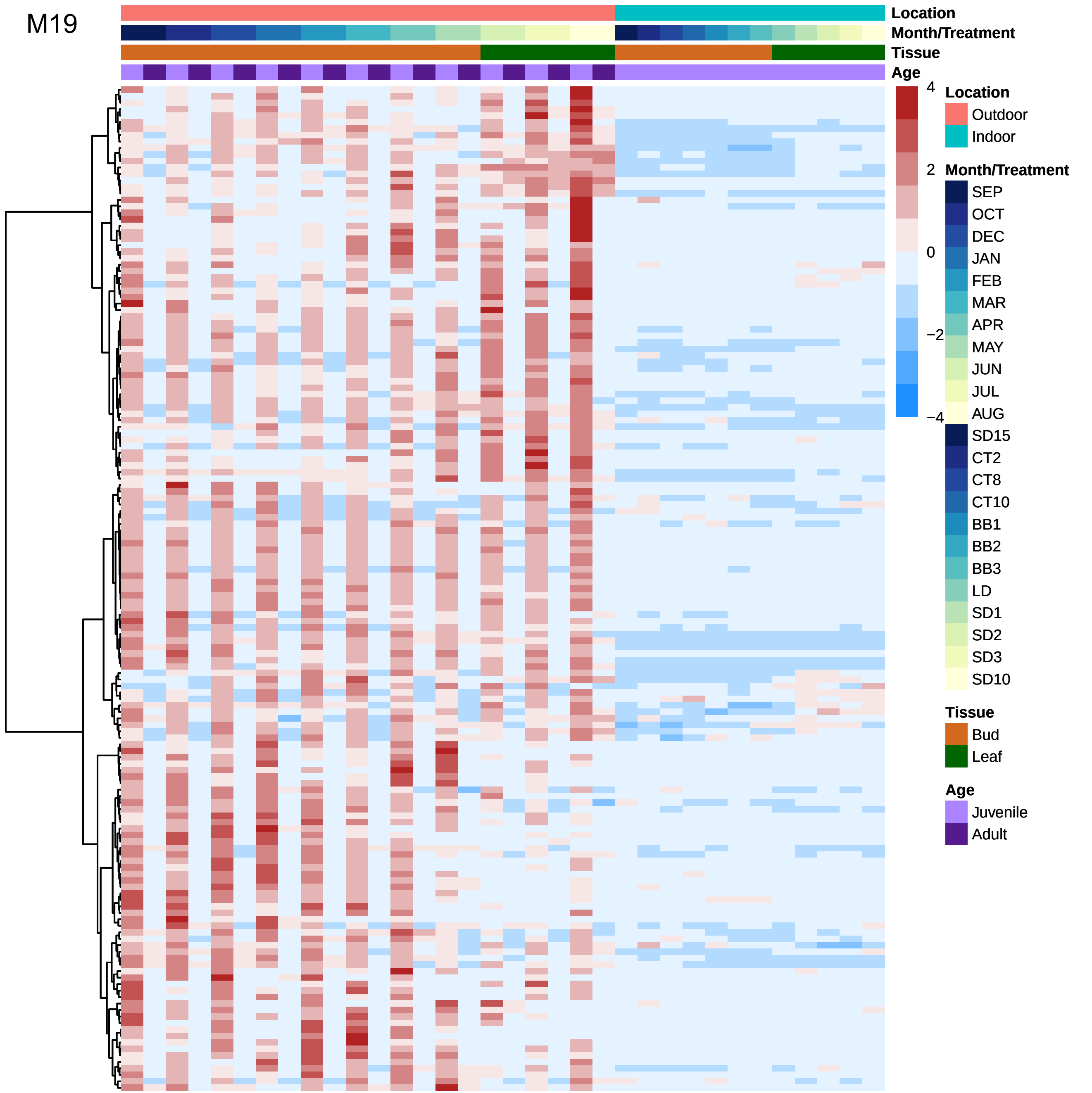

M19
Location
Month/Treatment
Tissue
Age
4
2
0
−2
−4
Location
Outdoor
Indoor
Month/Treatment
SEP
OCT
DEC
JAN
FEB
MAR
APR
MAY
JUN
JUL
AUG
SD15
CT2
CT8
CT10
BB1
BB2
BB3
LD
SD1
SD2
SD3
SD10
Tissue
Bud
Leaf
Age
Juvenile
Adult

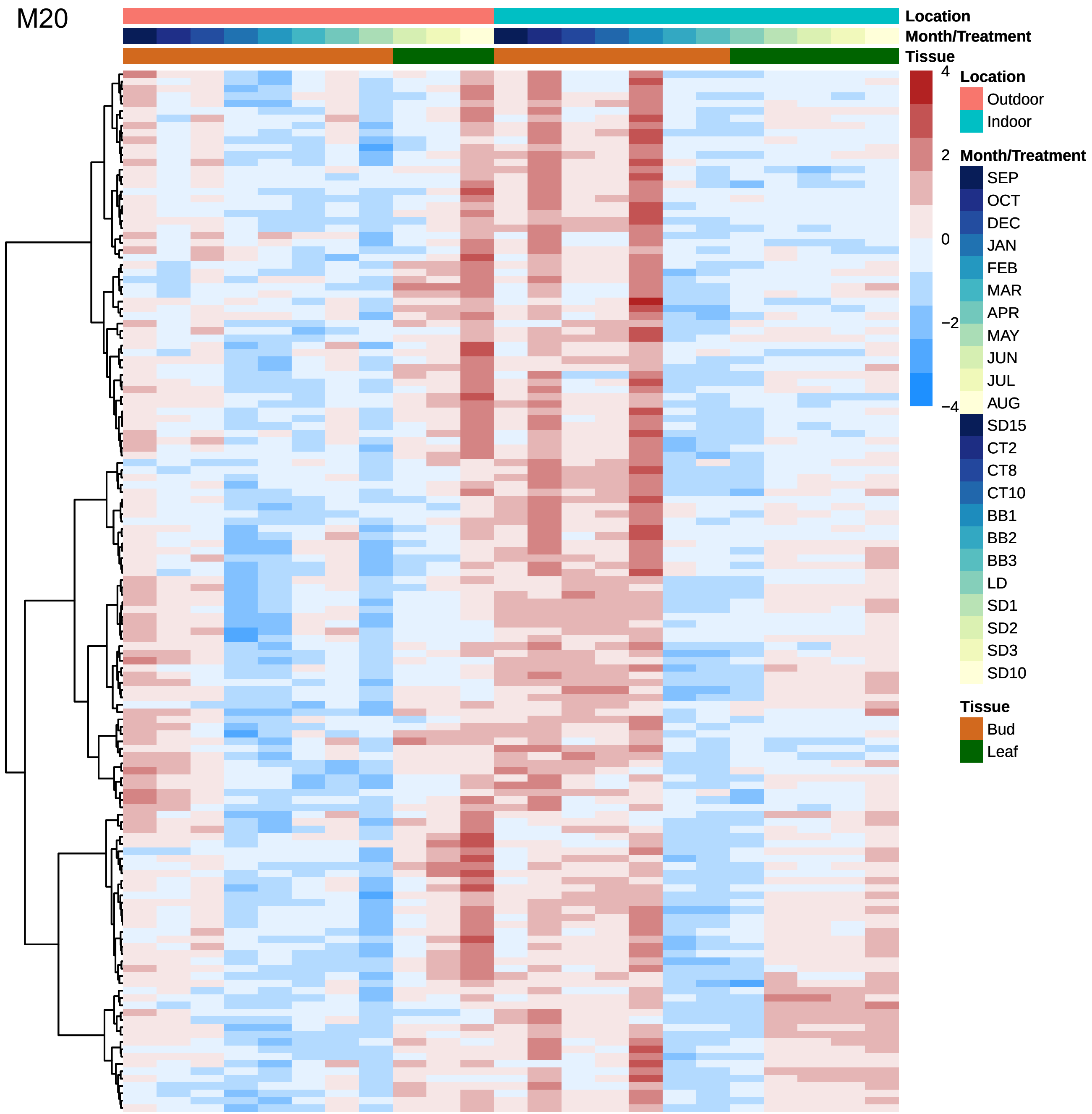

M20
Location
Month/Treatment
Tissue
4
2
0
−2
−4
Location
Outdoor
Indoor
Month/Treatment
SEP
OCT
DEC
JAN
FEB
MAR
APR
MAY
JUN
JUL
AUG
SD15
CT2
CT8
CT10
BB1
BB2
BB3
LD
SD1
SD2
SD3
SD10
Tissue
Bud
Leaf

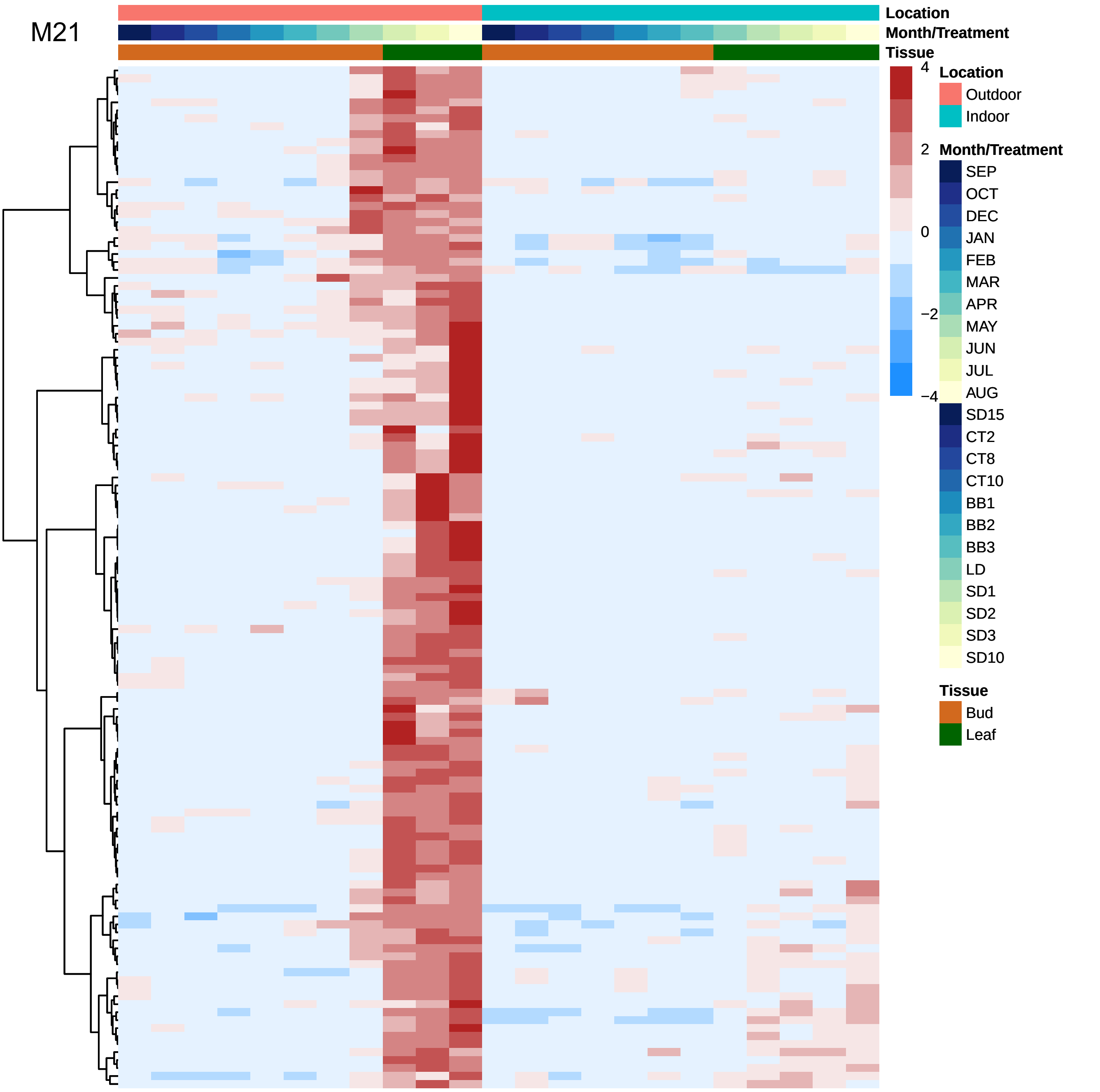

M21
Location
Month/Treatment
Tissue
4
2
0
−2
−4
Location
Outdoor
Indoor
Month/Treatment
SEP
OCT
DEC
JAN
FEB
MAR
APR
MAY
JUN
JUL
AUG
SD15
CT2
CT8
CT10
BB1
BB2
BB3
LD
SD1
SD2
SD3
SD10
Tissue
Bud
Leaf

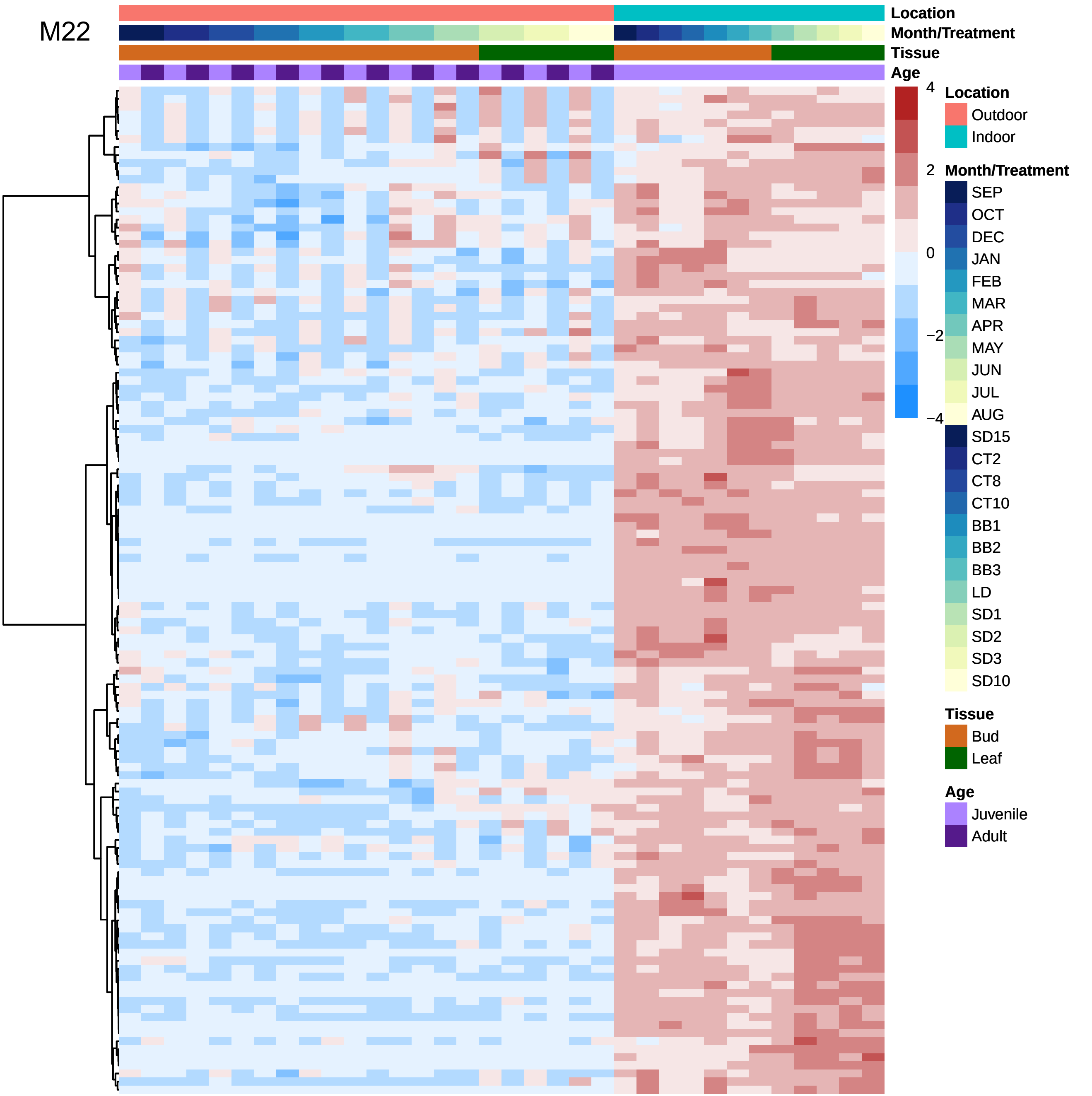
M22
Location
Month/Treatment
Tissue
Age
4
2
0
−2
−4
Location
Outdoor
Indoor
Month/Treatment
SEP
OCT
DEC
JAN
FEB
MAR
APR
MAY
JUN
JUL
AUG
SD15
CT2
CT8
CT10
BB1
BB2
BB3
LD
SD1
SD2
SD3
SD10
Tissue
Bud
Leaf
Age
Juvenile
Adult

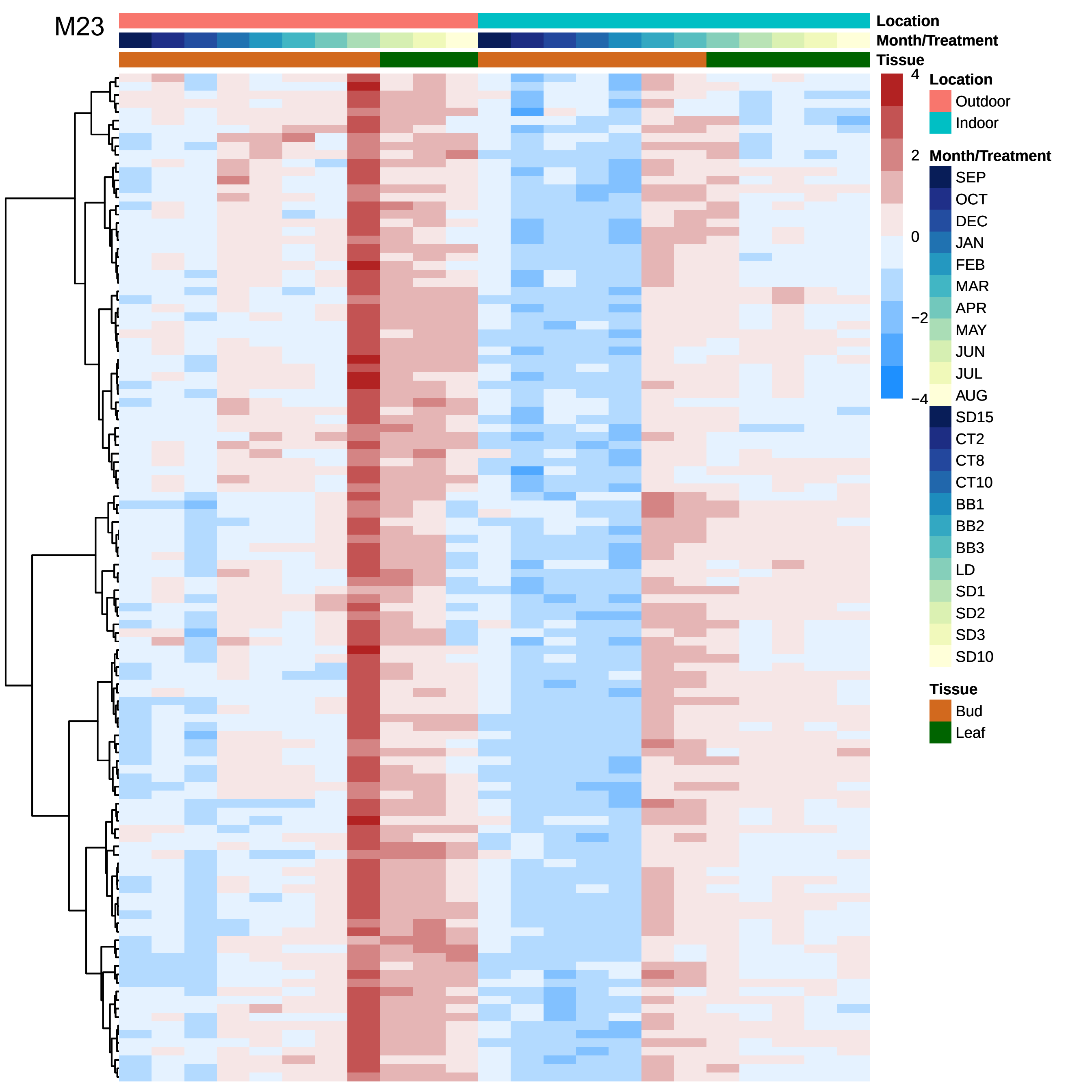
M23
Location
Month/Treatment
Tissue
4
2
0
−2
−4
Location
Outdoor
Indoor
Month/Treatment
SEP
OCT
DEC
JAN
FEB
MAR
APR
MAY
JUN
JUL
AUG
SD15
CT2
CT8
CT10
BB1
BB2
BB3
LD
SD1
SD2
SD3
SD10
Tissue
Bud
Leaf

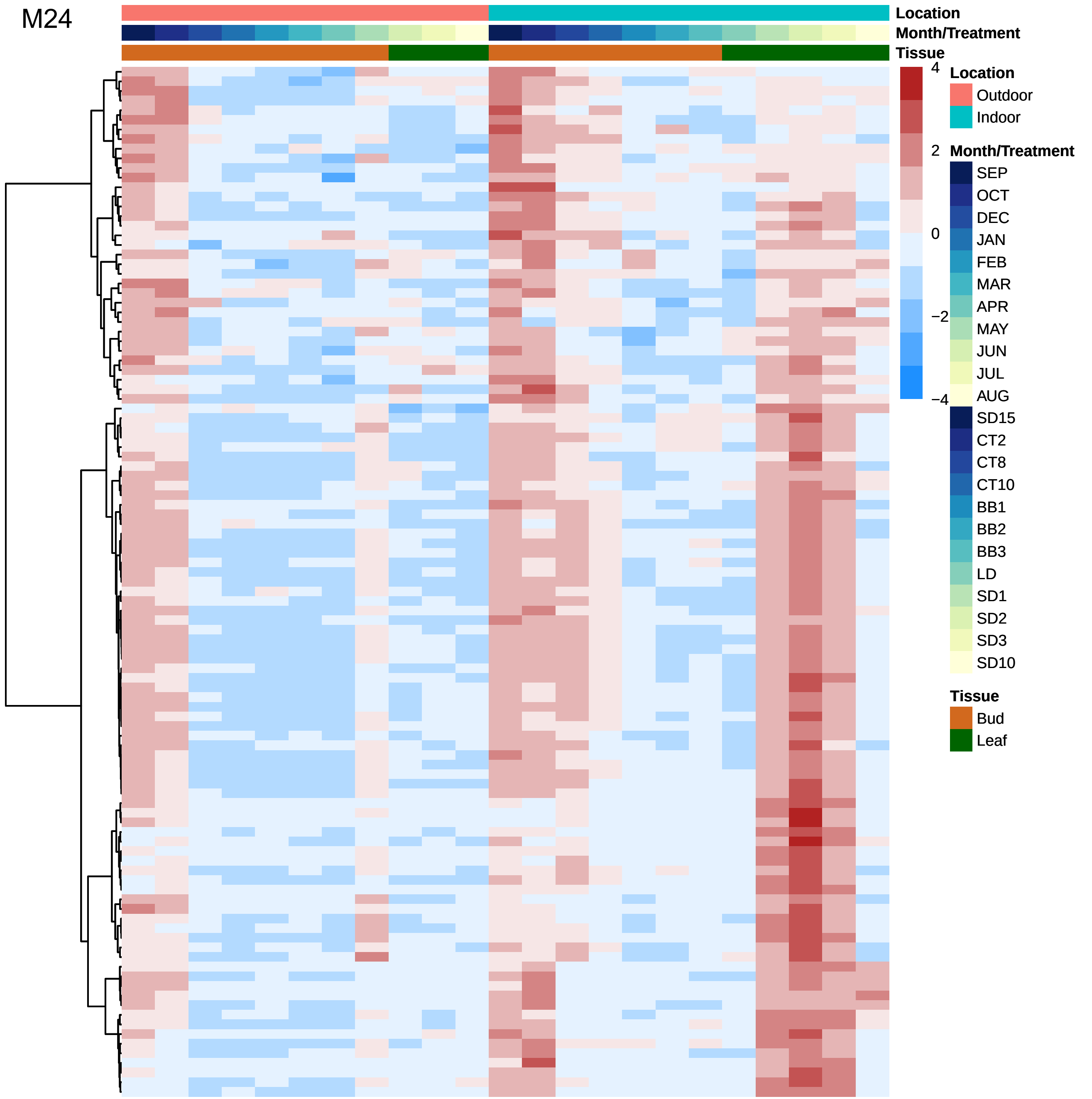
M24
Location
Month/Treatment
Tissue
4
2
0
−2
−4
Location
Outdoor
Indoor
Month/Treatment
SEP
OCT
DEC
JAN
FEB
MAR
APR
MAY
JUN
JUL
AUG
SD15
CT2
CT8
CT10
BB1
BB2
BB3
LD
SD1
SD2
SD3
SD10
Tissue
Bud
Leaf

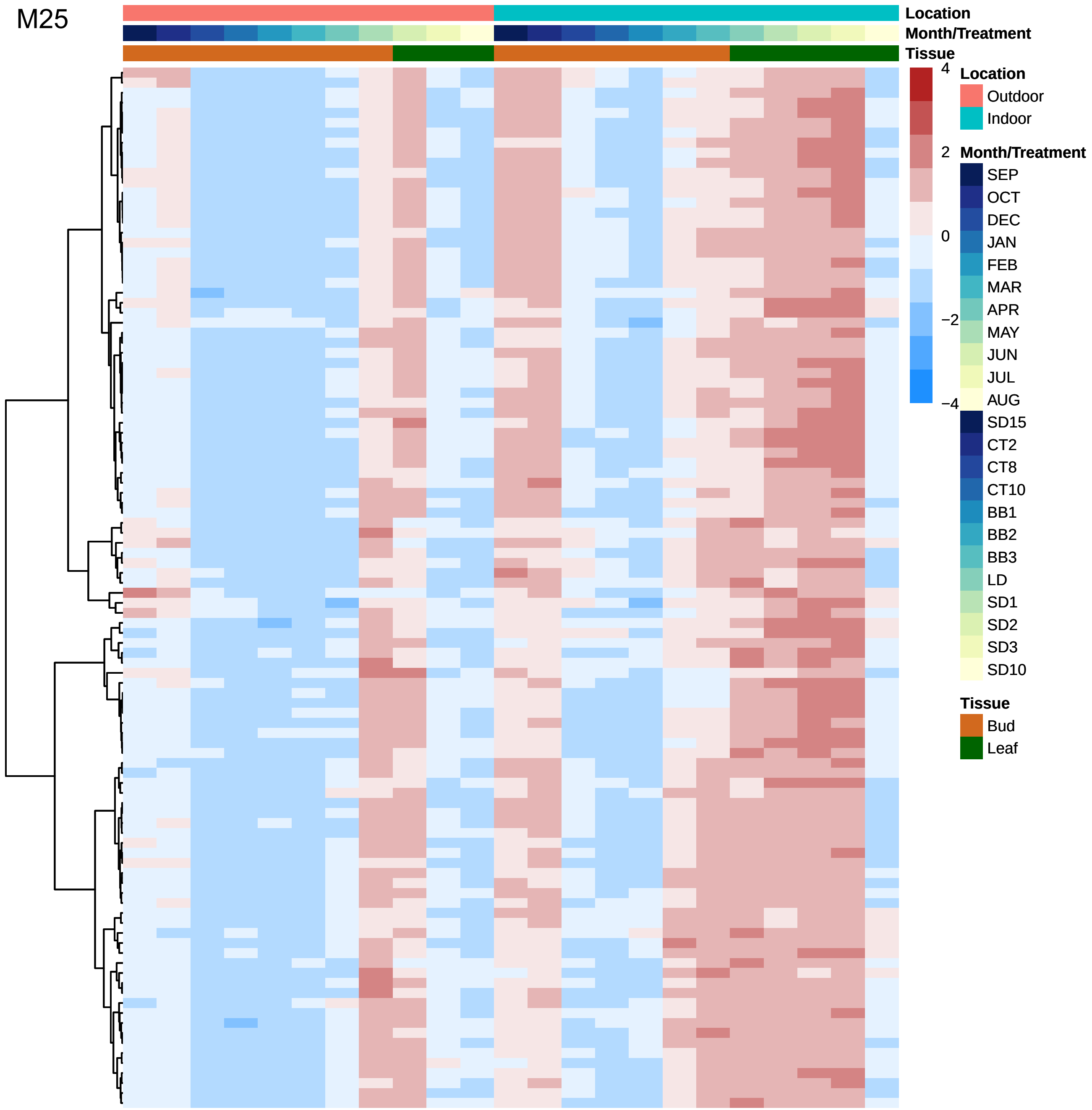
M25
Location
Month/Treatment
Tissue
4
2
0
−2
−4
Location
Outdoor
Indoor
Month/Treatment
SEP
OCT
DEC
JAN
FEB
MAR
APR
MAY
JUN
JUL
AUG
SD15
CT2
CT8
CT10
BB1
BB2
BB3
LD
SD1
SD2
SD3
SD10
Tissue
Bud
Leaf

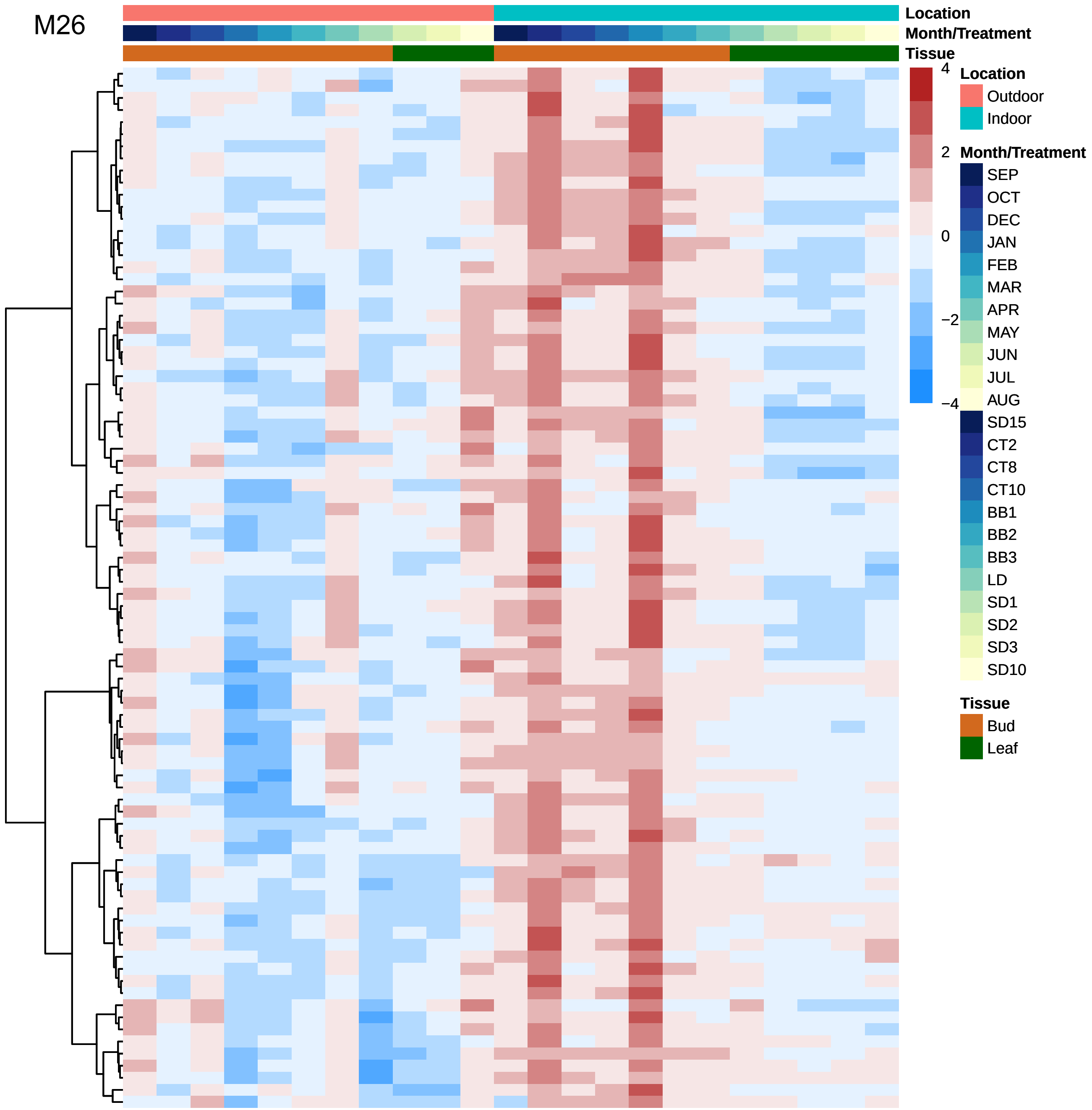
M26
Location
Month/Treatment
Tissue
4
2
0
−2
−4
Location
Outdoor
Indoor
Month/Treatment
SEP
OCT
DEC
JAN
FEB
MAR
APR
MAY
JUN
JUL
AUG
SD15
CT2
CT8
CT10
BB1
BB2
BB3
LD
SD1
SD2
SD3
SD10
Tissue
Bud
Leaf

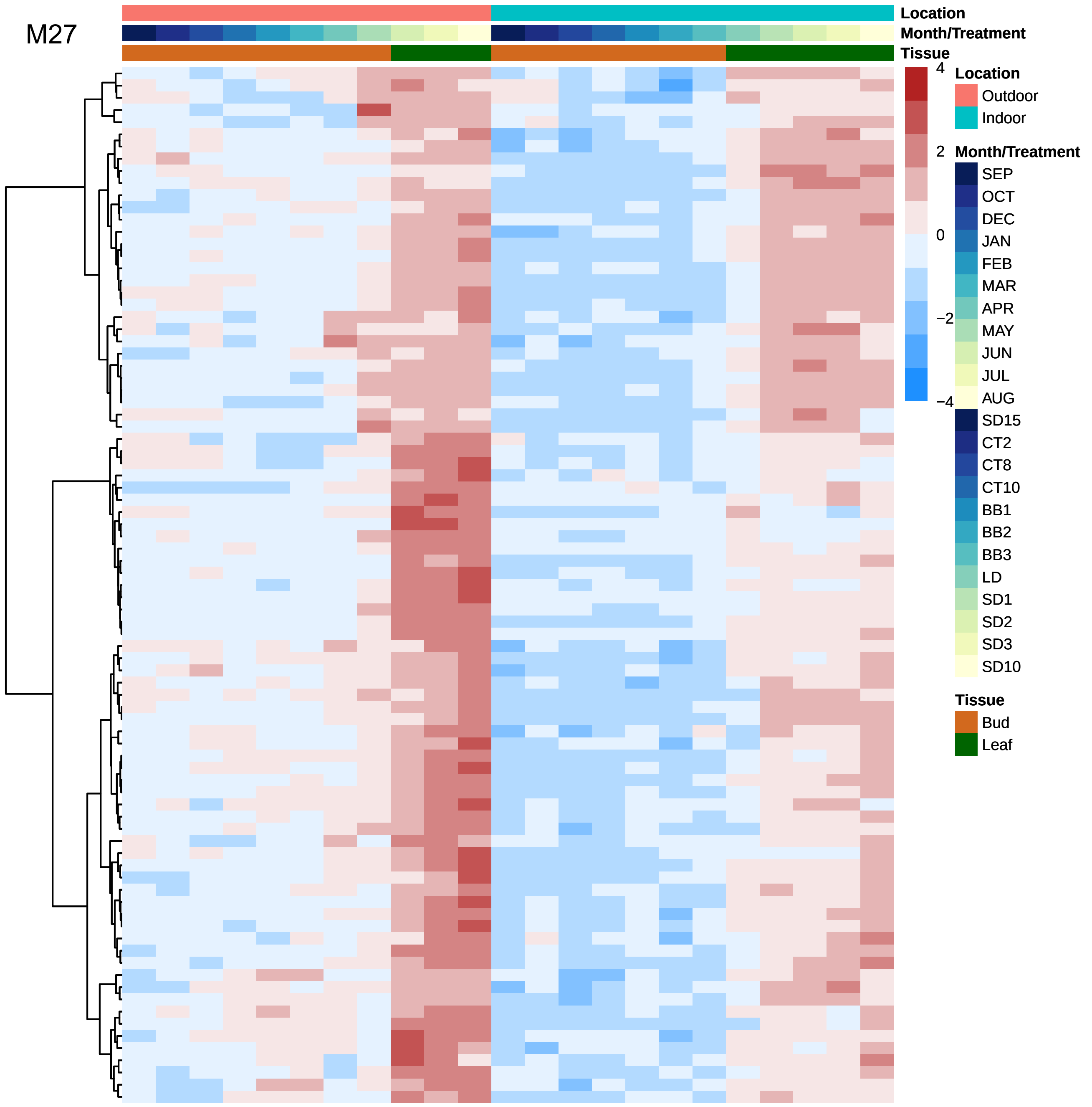
M27
Location
Month/Treatment
Tissue
4
2
0
−2
−4
Location
Outdoor
Indoor
Month/Treatment
SEP
OCT
DEC
JAN
FEB
MAR
APR
MAY
JUN
JUL
AUG
SD15
CT2
CT8
CT10
BB1
BB2
BB3
LD
SD1
SD2
SD3
SD10
Tissue
Bud
Leaf

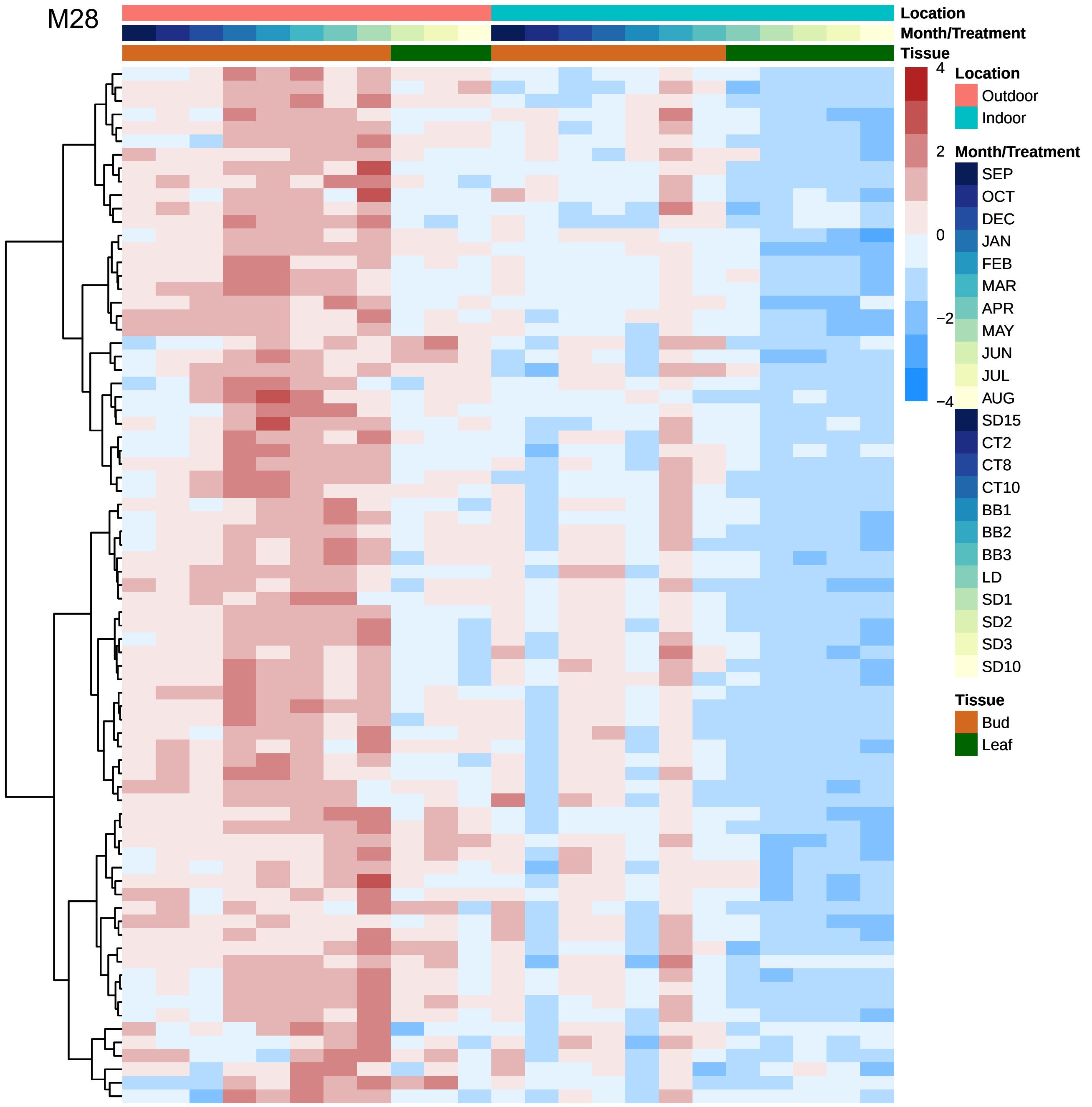
M28
Location
Month/Treatment
Tissue
4
2
0
−2
−4
Location
Outdoor
Indoor
Month/Treatment
SEP
OCT
DEC
JAN
FEB
MAR
APR
MAY
JUN
JUL
AUG
SD15
CT2
CT8
CT10
BB1
BB2
BB3
LD
SD1
SD2
SD3
SD10
Tissue
Bud
Leaf

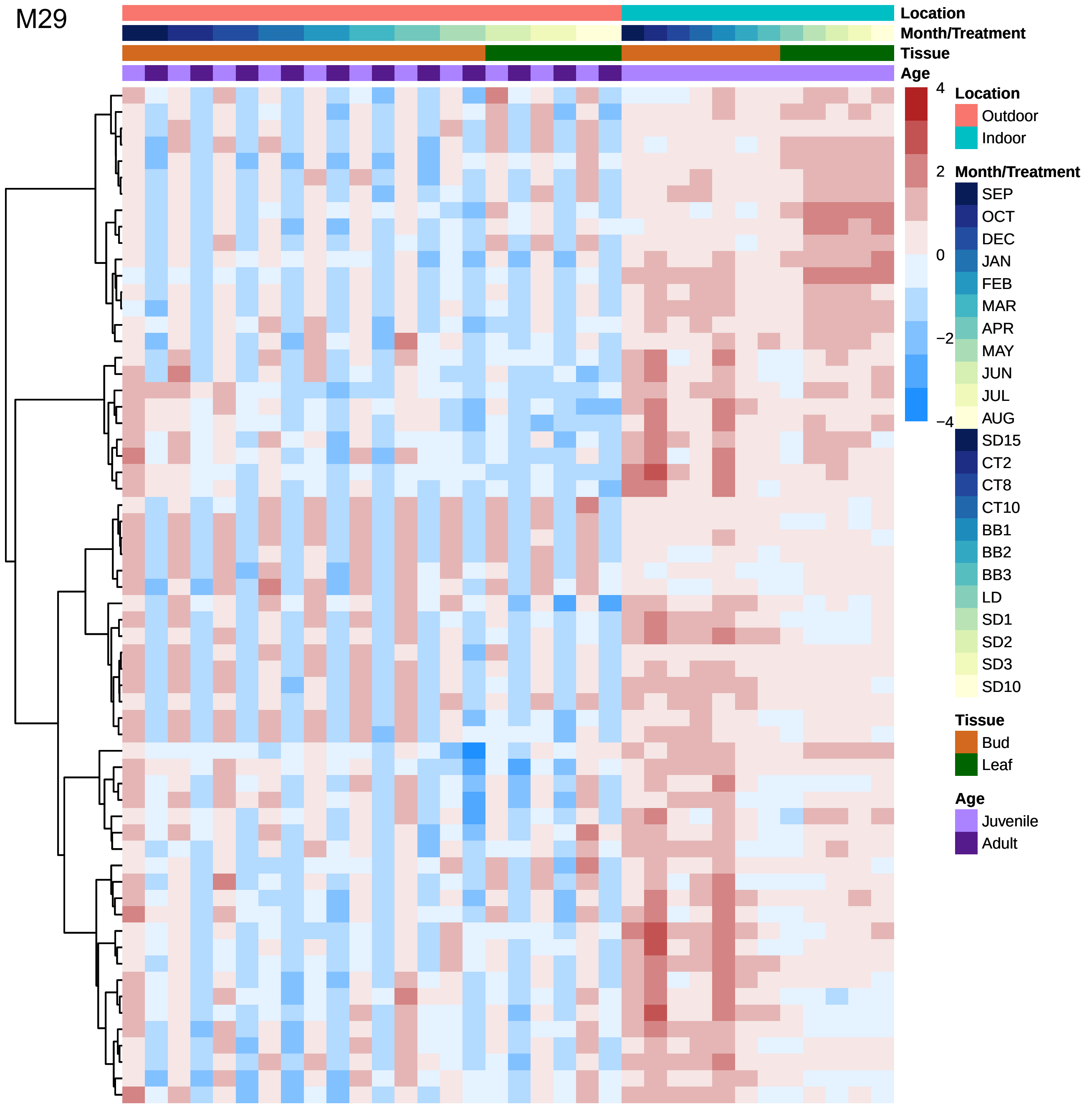
M29
Location
Month/Treatment
Tissue
Age
4
2
0
−2
−4
Location
Outdoor
Indoor
Month/Treatment
SEP
OCT
DEC
JAN
FEB
MAR
APR
MAY
JUN
JUL
AUG
SD15
CT2
CT8
CT10
BB1
BB2
BB3
LD
SD1
SD2
SD3
SD10
Tissue
Bud
Leaf
Age
Juvenile
Adult

M30

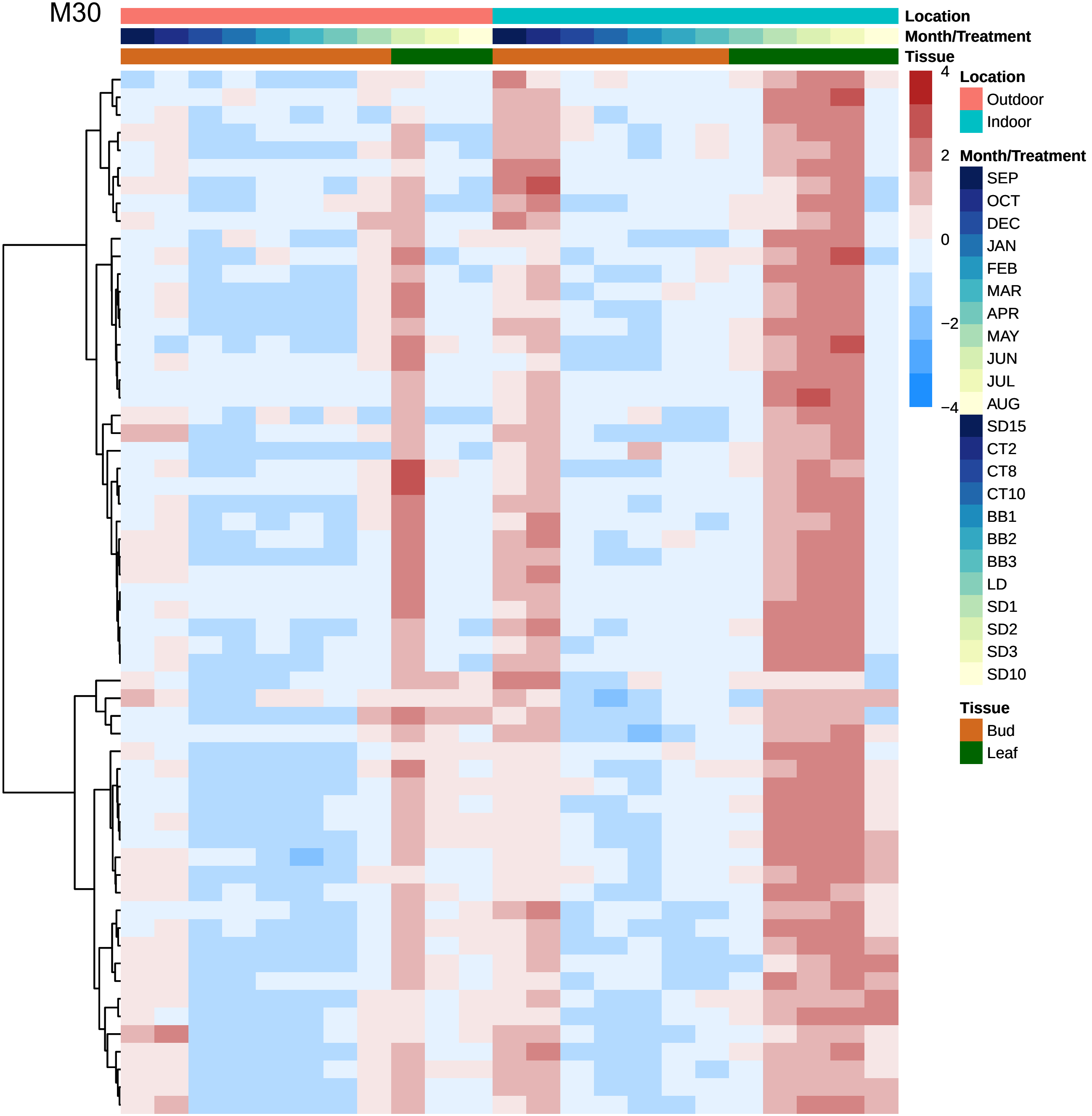
Location
Month/Treatment
Tissue
4
2
0
−2
−4
Location
Outdoor
Indoor
Month/Treatment
SEP
OCT
DEC
JAN
FEB
MAR
APR
MAY
JUN
JUL
AUG
SD15
CT2
CT8
CT10
BB1
BB2
BB3
LD
SD1
SD2
SD3
SD10
Tissue
Bud
Leaf

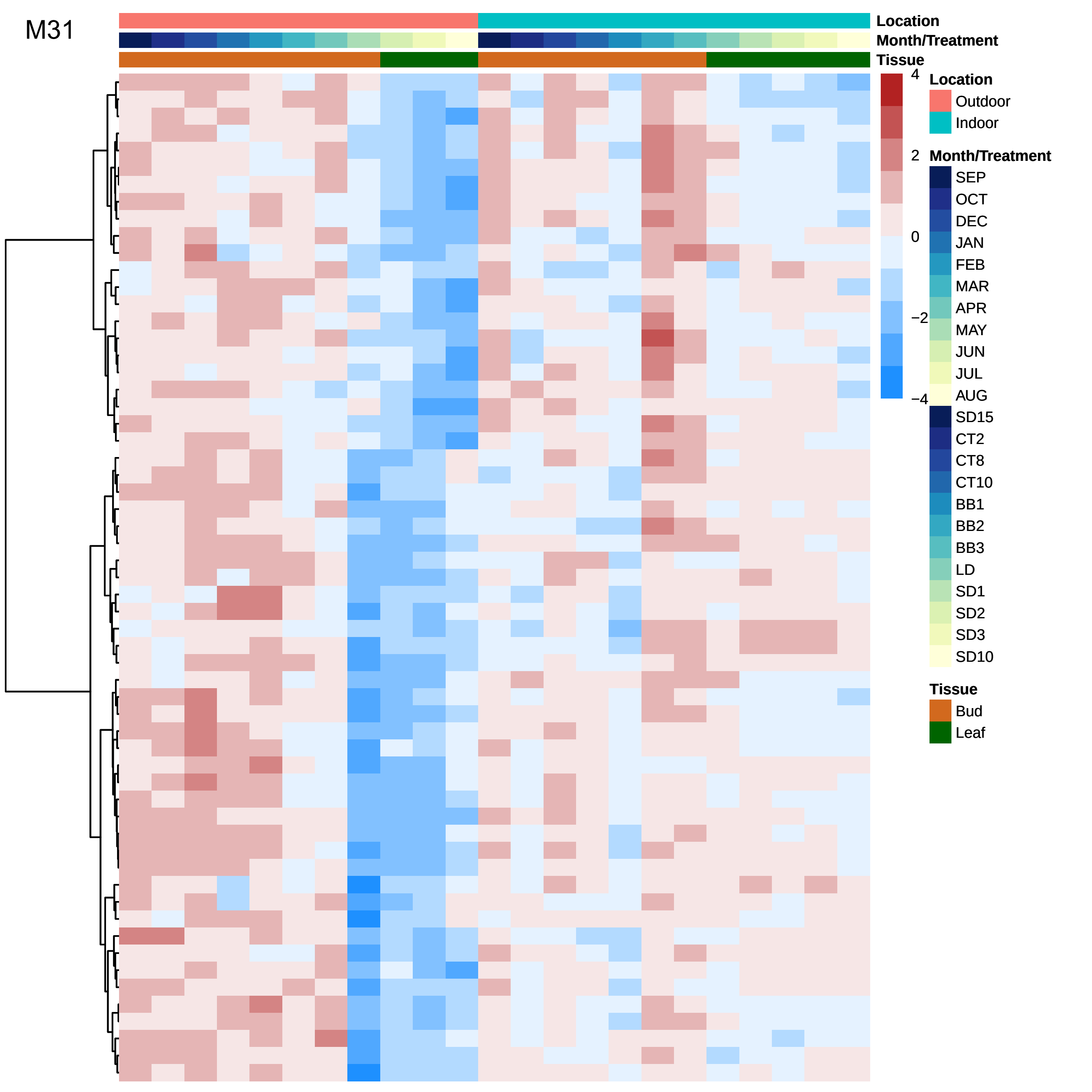
M31
Location
Month/Treatment
Tissue
4
2
0
−2
−4
Location
Outdoor
Indoor
Month/Treatment
SEP
OCT
DEC
JAN
FEB
MAR
APR
MAY
JUN
JUL
AUG
SD15
CT2
CT8
CT10
BB1
BB2
BB3
LD
SD1
SD2
SD3
SD10
Tissue
Bud
Leaf

M32

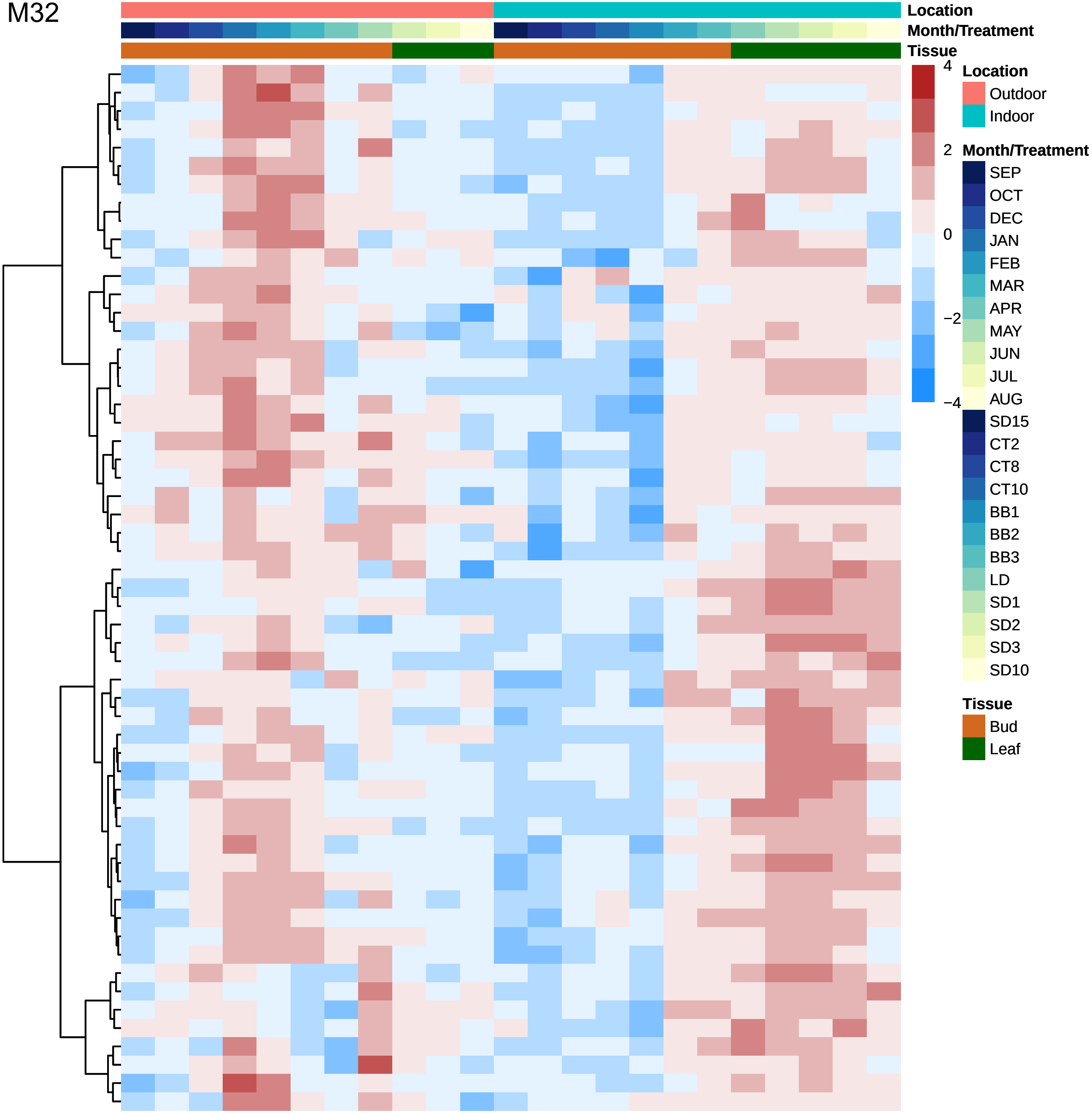
Location
Month/Treatment
Tissue
4
2
0
−2
−4
Location
Outdoor
Indoor
Month/Treatment
SEP
OCT
DEC
JAN
FEB
MAR
APR
MAY
JUN
JUL
AUG
SD15
CT2
CT8
CT10
BB1
BB2
BB3
LD
SD1
SD2
SD3
SD10
Tissue
Bud
Leaf

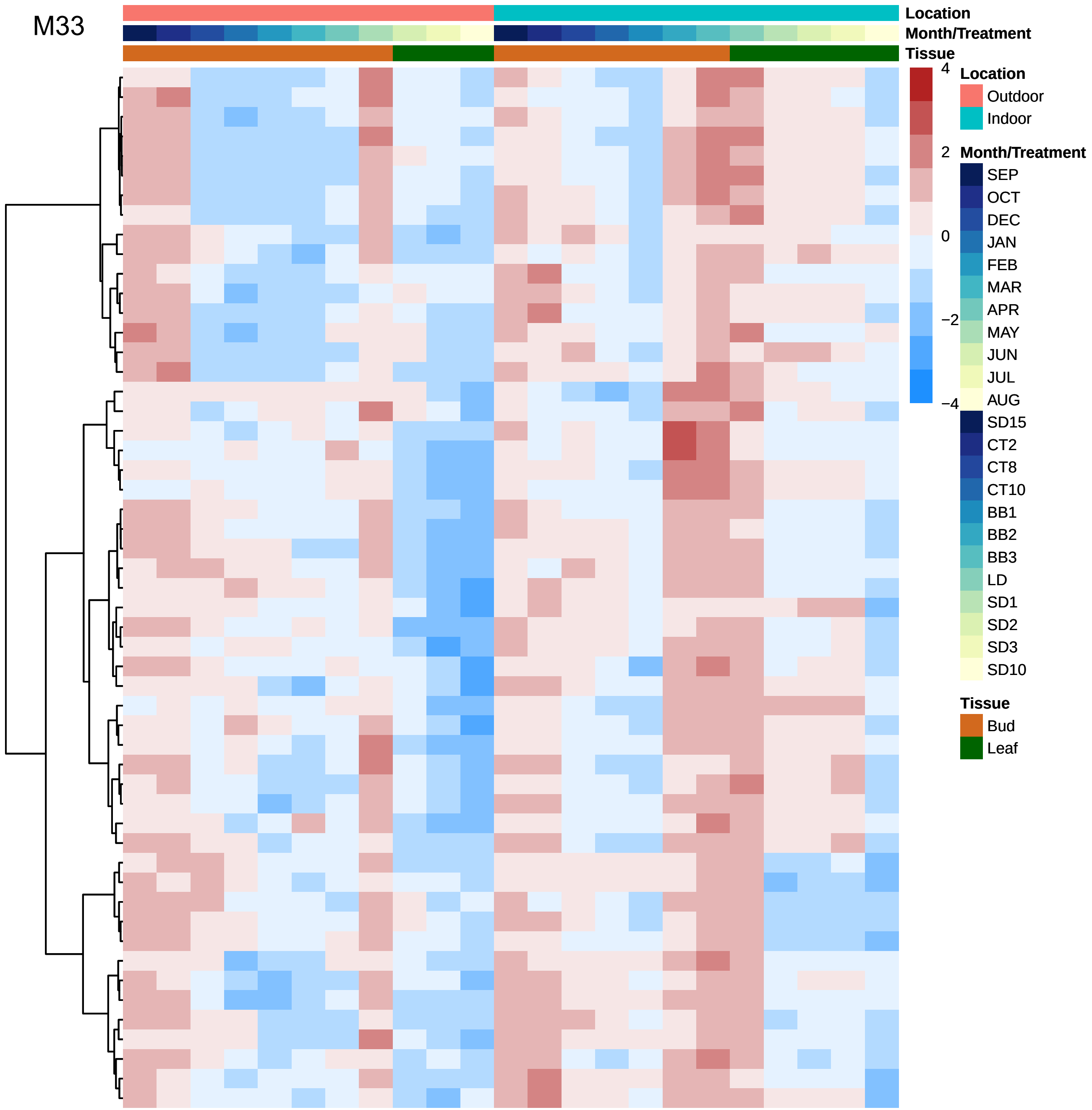
M33
Location
Month/Treatment
Tissue
4
2
0
−2
−4
Location
Outdoor
Indoor
Month/Treatment
SEP
OCT
DEC
JAN
FEB
MAR
APR
MAY
JUN
JUL
AUG
SD15
CT2
CT8
CT10
BB1
BB2
BB3
LD
SD1
SD2
SD3
SD10
Tissue
Bud
Leaf

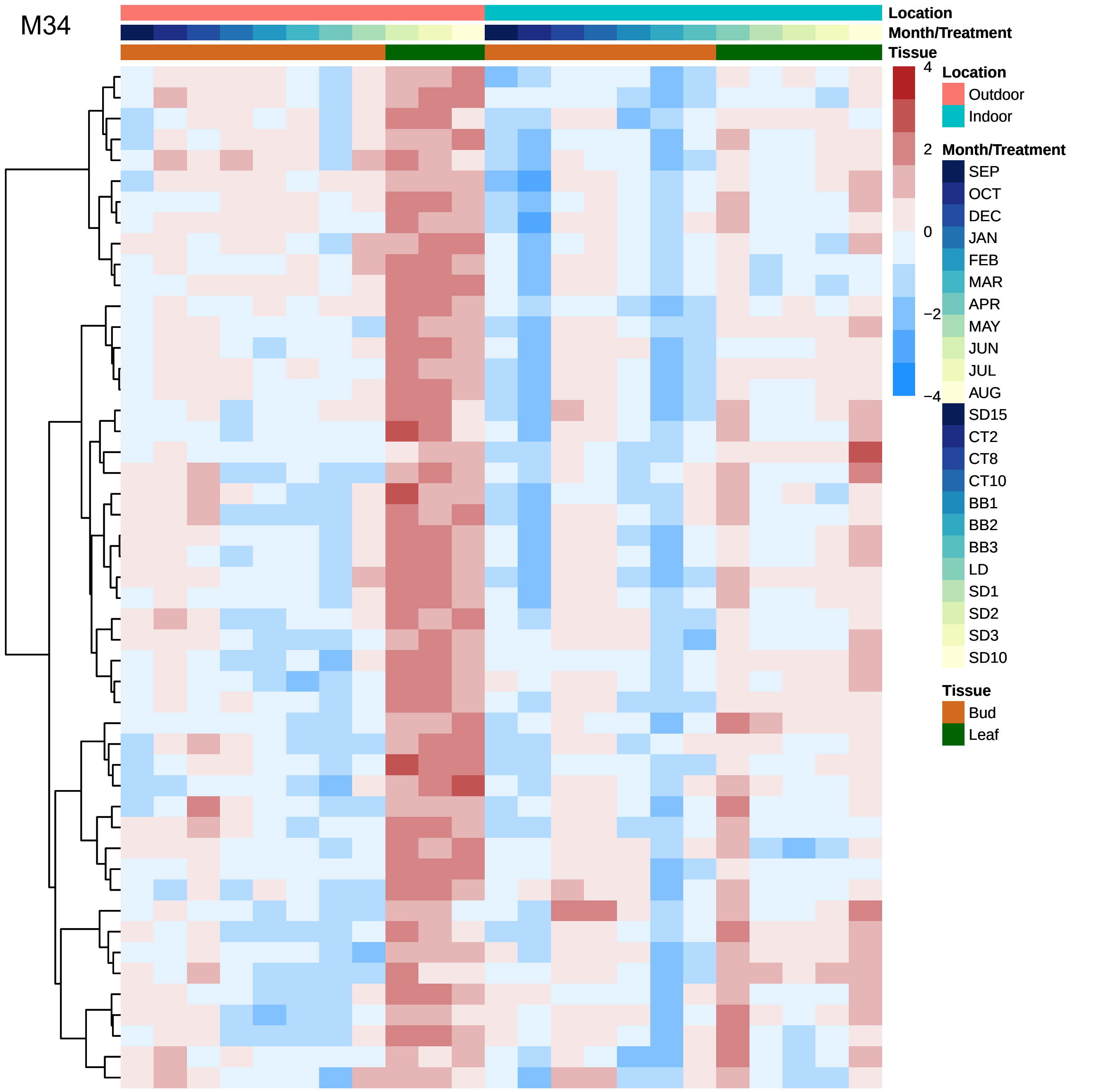

M34
Location
Month/Treatment
Tissue
4
2
0
−2
−4
Location
Outdoor
Indoor
Month/Treatment
SEP
OCT
DEC
JAN
FEB
MAR
APR
MAY
JUN
JUL
AUG
SD15
CT2
CT8
CT10
BB1
BB2
BB3
LD
SD1
SD2
SD3
SD10
Tissue
Bud
Leaf

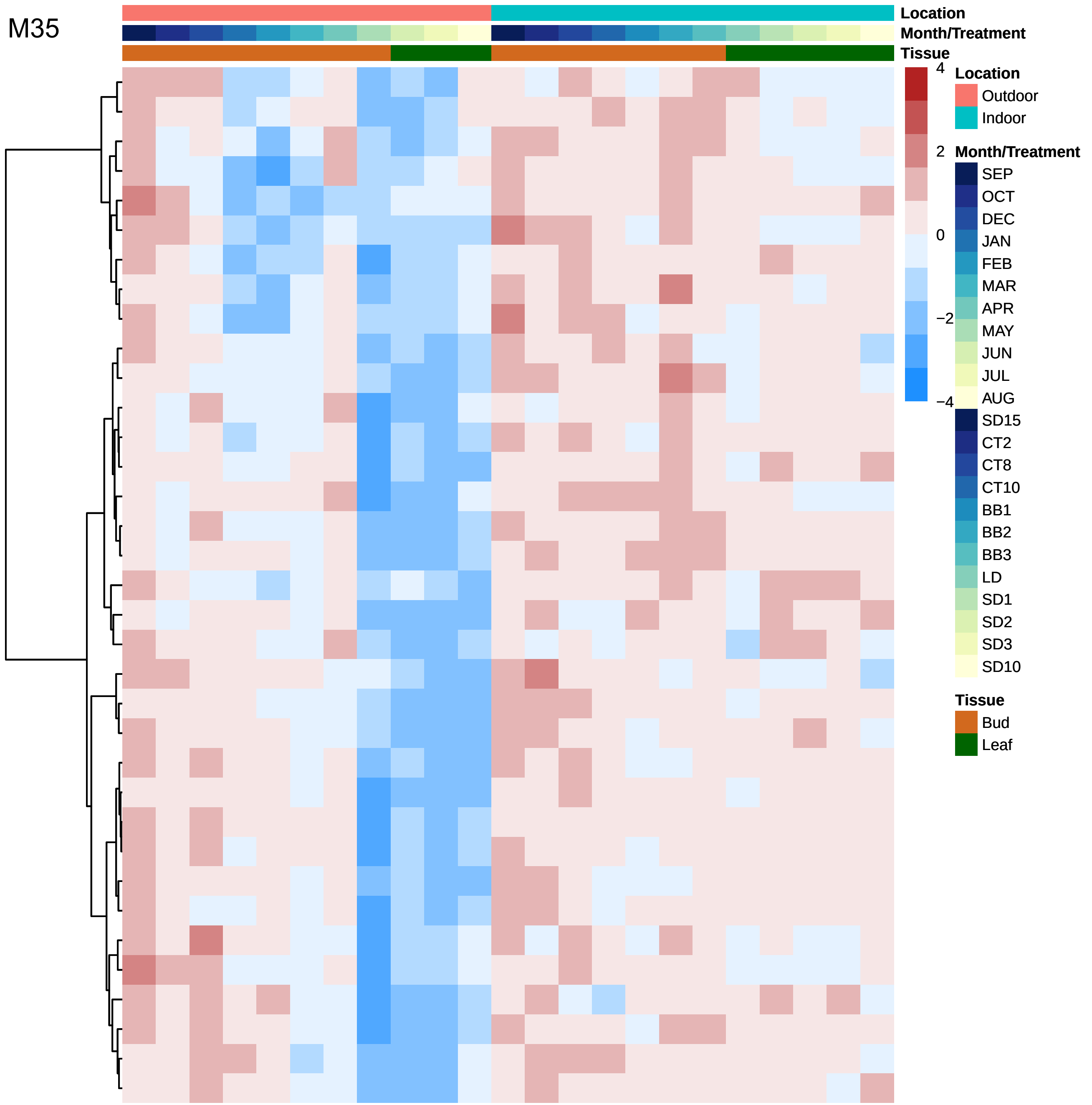

M35
Location
Month/Treatment
Tissue
4
2
0
−2
−4
Location
Outdoor
Indoor
Month/Treatment
SEP
OCT
DEC
JAN
FEB
MAR
APR
MAY
JUN
JUL
AUG
SD15
CT2
CT8
CT10
BB1
BB2
BB3
LD
SD1
SD2
SD3
SD10
Tissue
Bud
Leaf

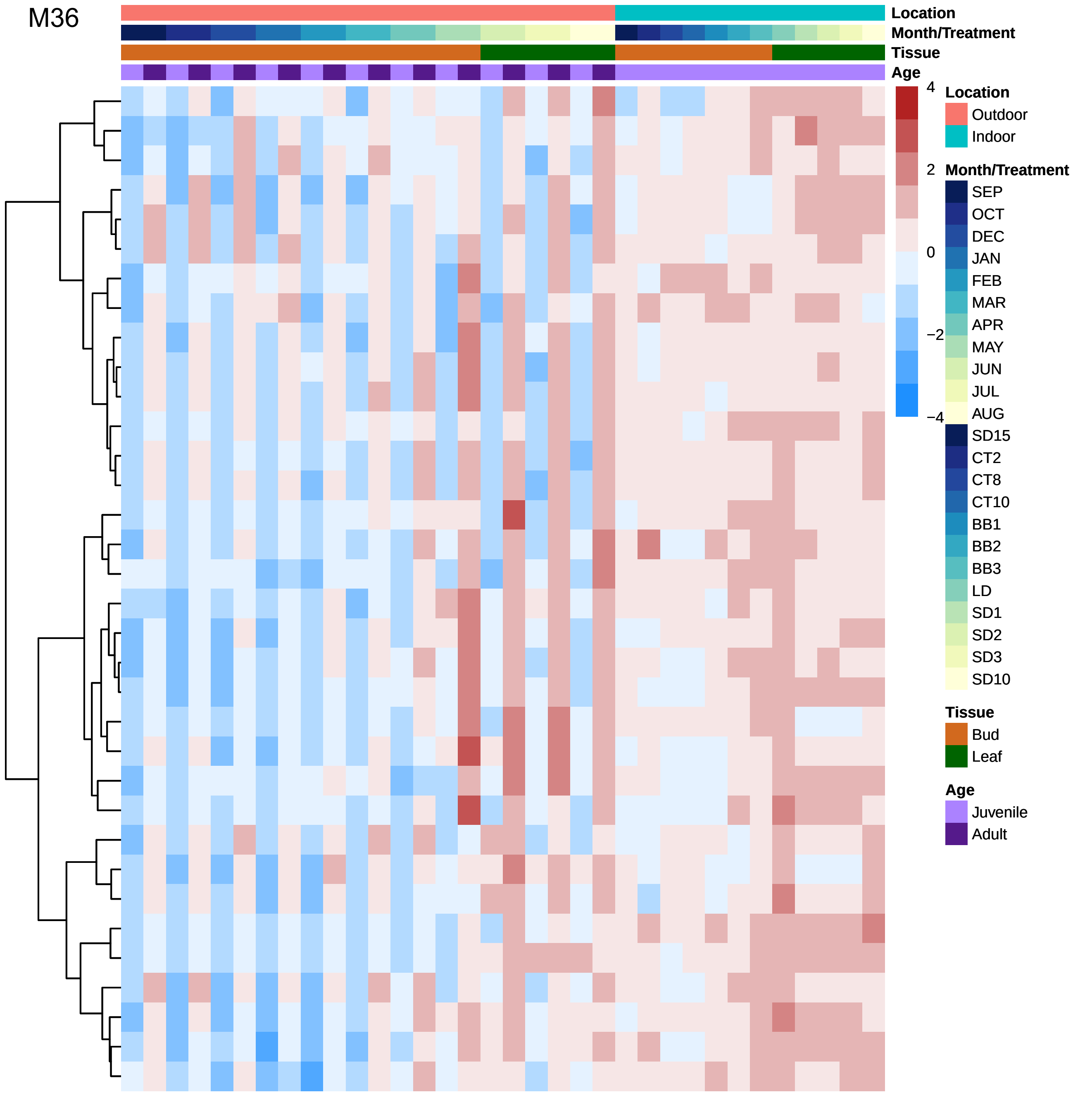
M36
Location
Month/Treatment
Tissue
Age
4
2
0
−2
−4
Location
Outdoor
Indoor
Month/Treatment
SEP
OCT
DEC
JAN
FEB
MAR
APR
MAY
JUN
JUL
AUG
SD15
CT2
CT8
CT10
BB1
BB2
BB3
LD
SD1
SD2
SD3
SD10
Tissue
Bud
Leaf
Age
Juvenile
Adult

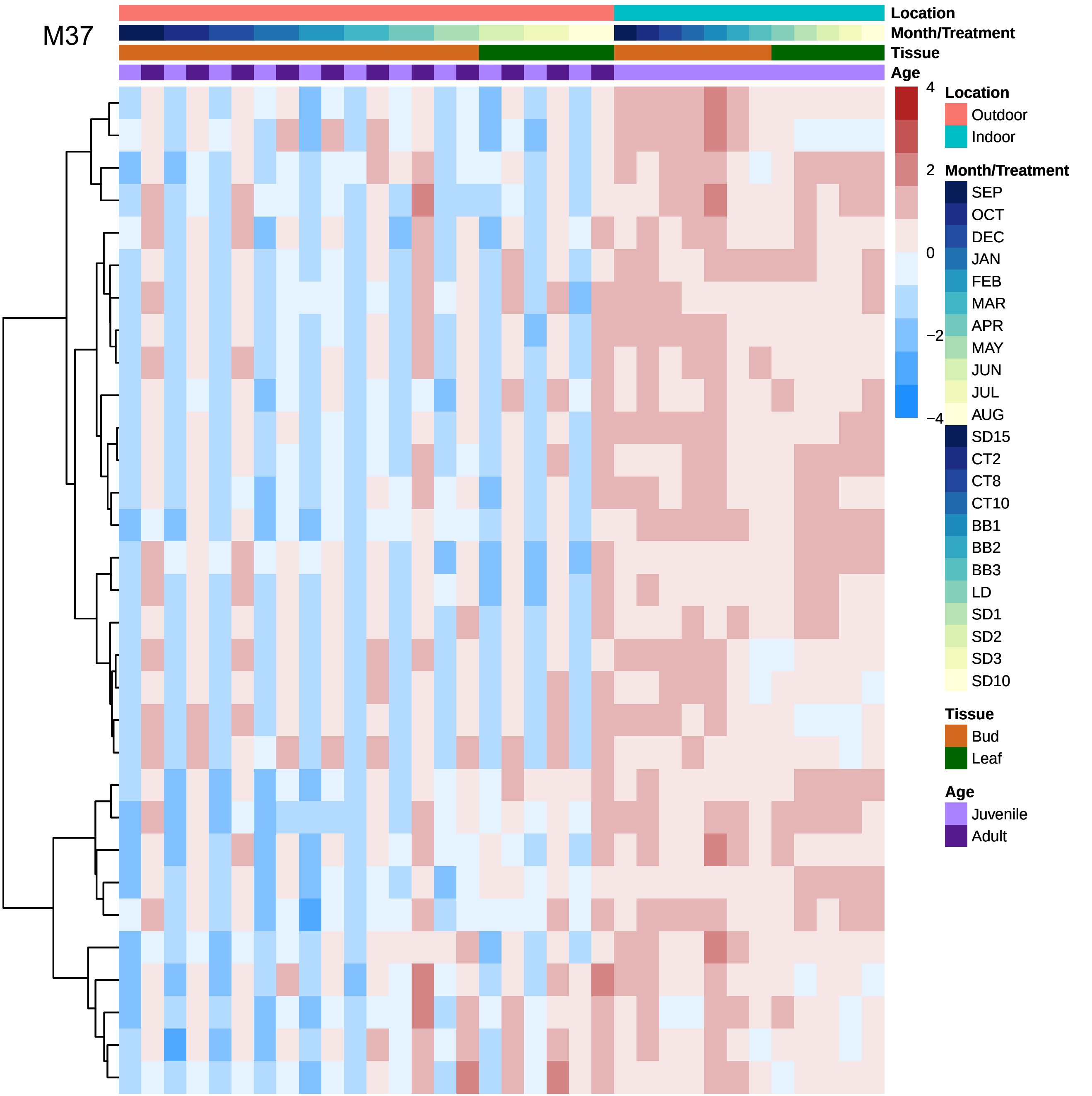
M37
Location
Month/Treatment
Tissue
Age
4
2
0
−2
−4
Location
Outdoor
Indoor
Month/Treatment
SEP
OCT
DEC
JAN
FEB
MAR
APR
MAY
JUN
JUL
AUG
SD15
CT2
CT8
CT10
BB1
BB2
BB3
LD
SD1
SD2
SD3
SD10
Tissue
Bud
Leaf
Age
Juvenile
Adult

M38

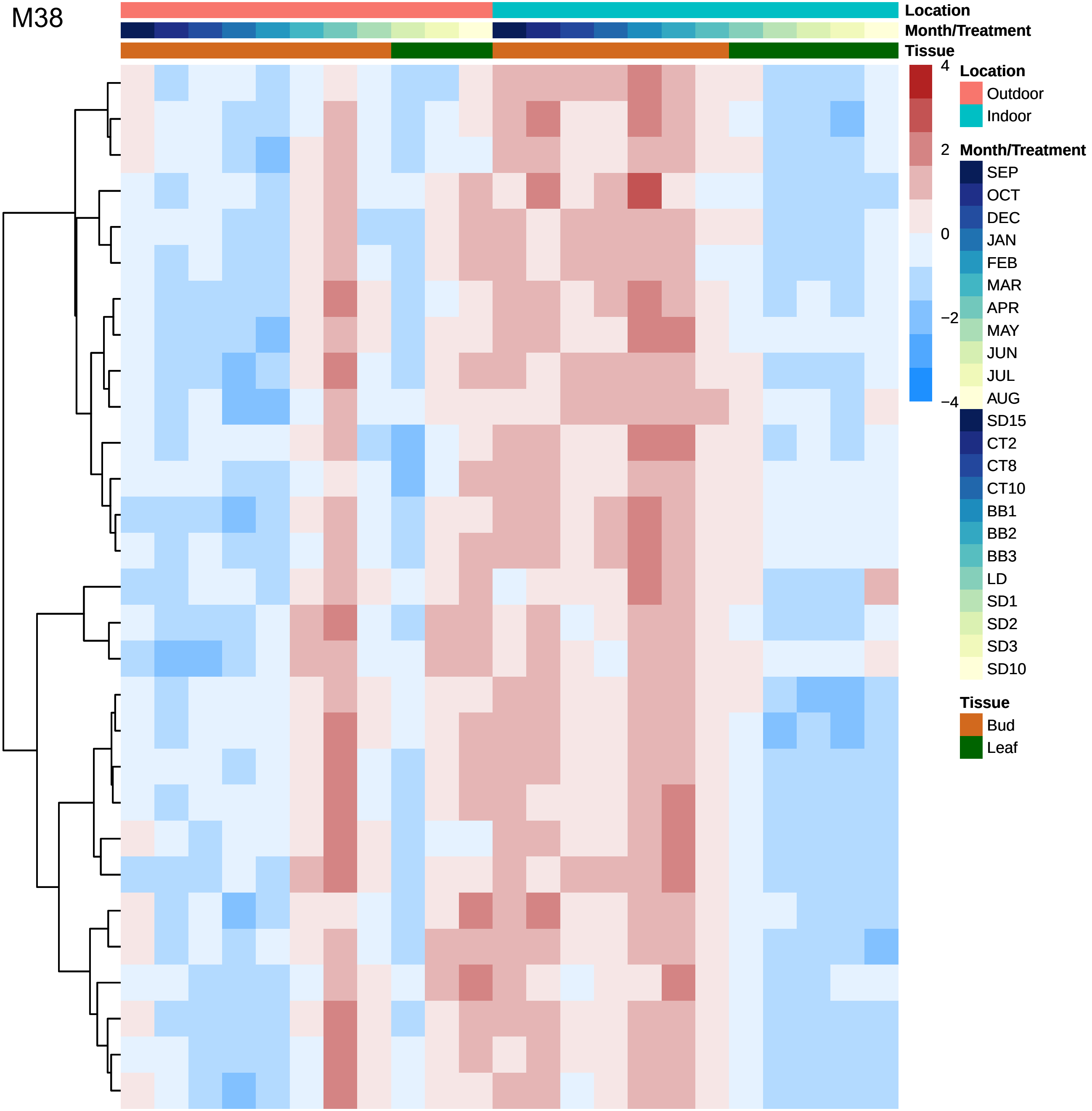
Location
Month/Treatment
Tissue
4
2
0
−2
−4
Location
Outdoor
Indoor
Month/Treatment
SEP
OCT
DEC
JAN
FEB
MAR
APR
MAY
JUN
JUL
AUG
SD15
CT2
CT8
CT10
BB1
BB2
BB3
LD
SD1
SD2
SD3
SD10
Tissue
Bud
Leaf

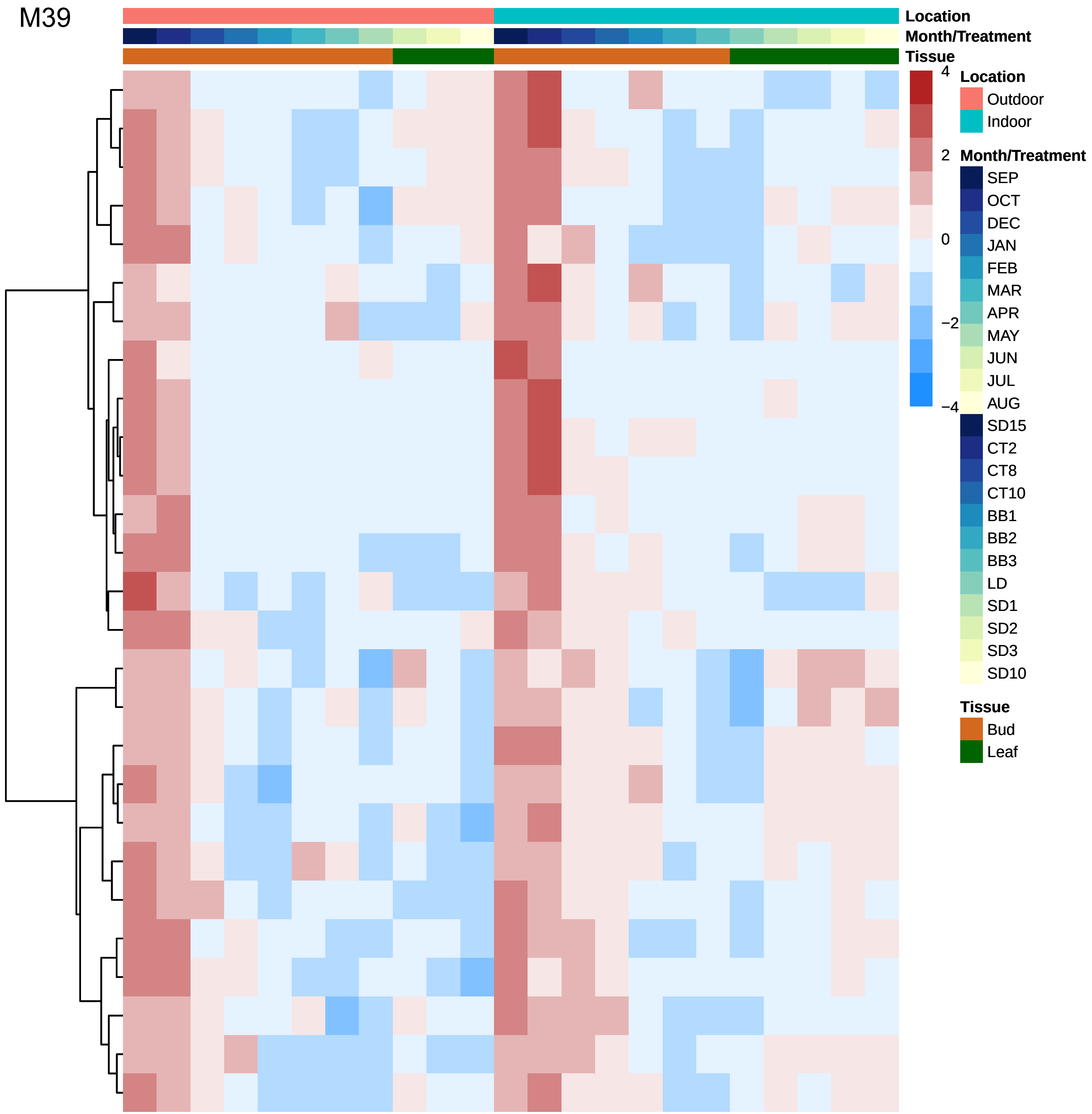
M39
Location
Month/Treatment
Tissue
Location
Outdoor
Indoor
Month/Treatment
SEP
OCT
DEC
JAN
FEB
MAR
APR
MAY
JUN
JUL
AUG
SD15
CT2
CT8
CT10
BB1
BB2
BB3
LD
SD1
SD2
SD3
SD10
Tissue
Bud
Leaf
4
2
0
−2
−4

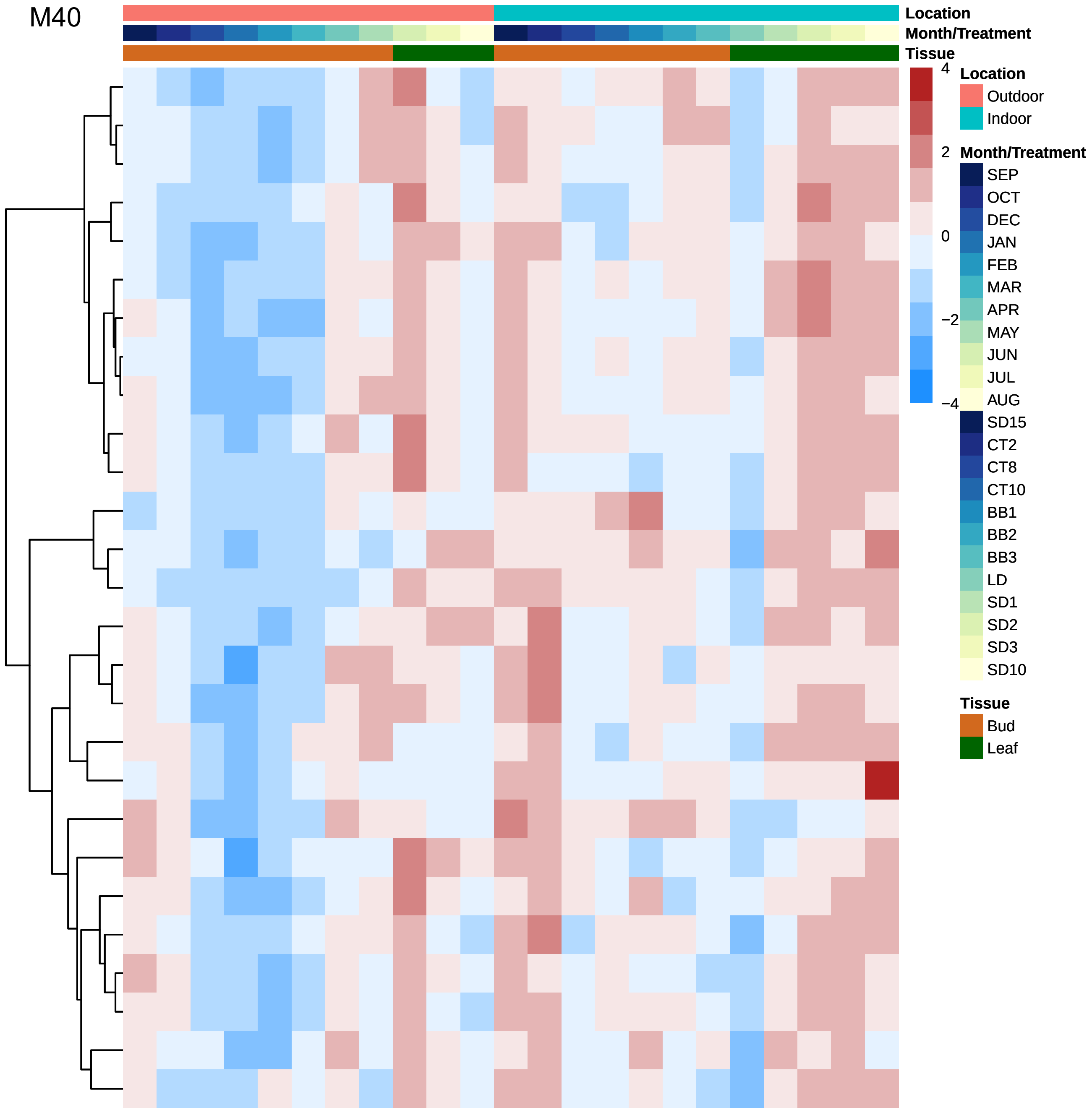

M40
Location
Month/Treatment
Tissue
4
2
0
−2
−4
Location
Outdoor
Indoor
Month/Treatment
SEP
OCT
DEC
JAN
FEB
MAR
APR
MAY
JUN
JUL
AUG
SD15
CT2
CT8
CT10
BB1
BB2
BB3
LD
SD1
SD2
SD3
SD10
Tissue
Bud
Leaf

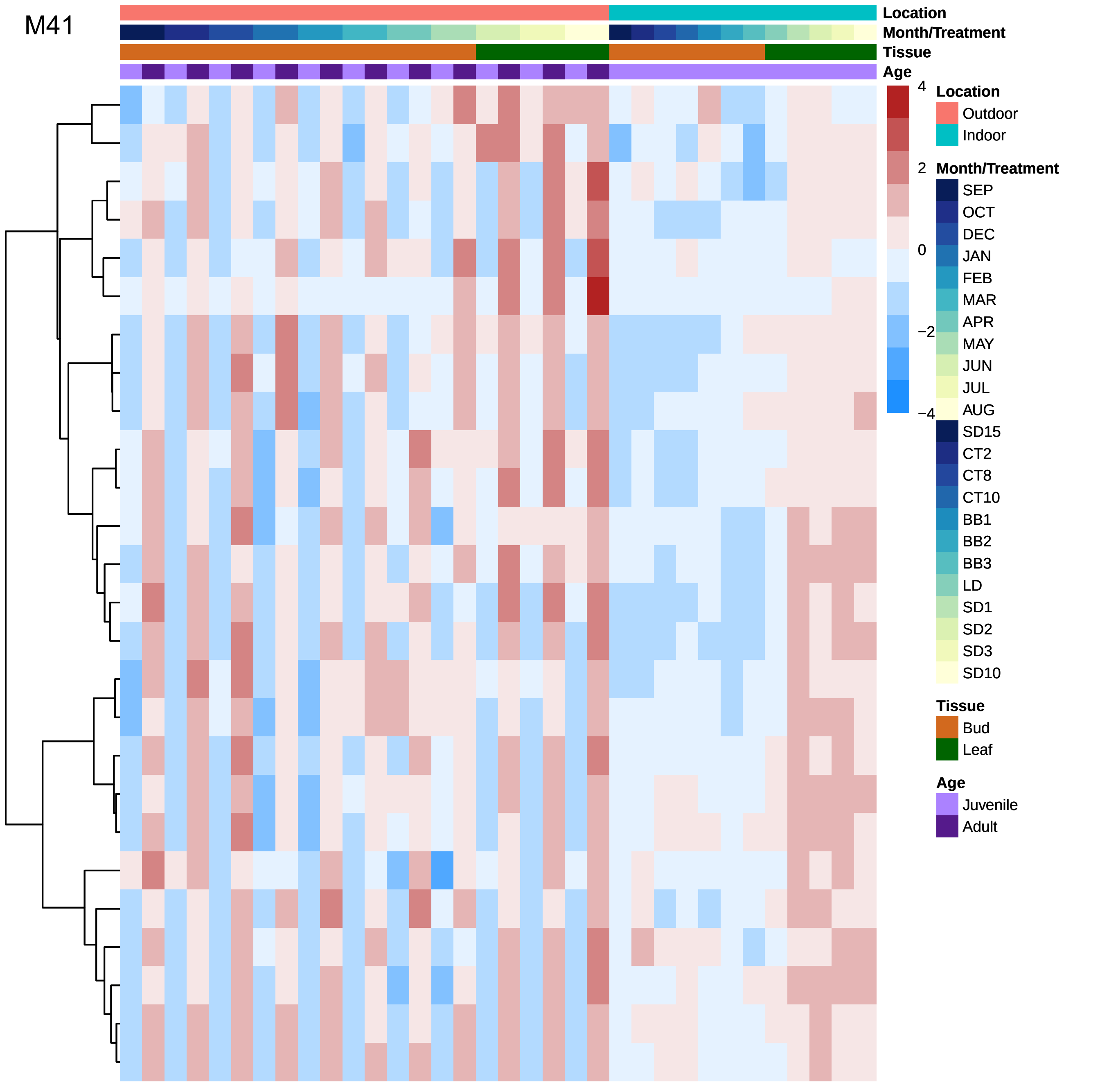
M41
Location
Month/Treatment
Tissue
Age
4
2
0
−2
−4
Location
Outdoor
Indoor
Month/Treatment
SEP
OCT
DEC
JAN
FEB
MAR
APR
MAY
JUN
JUL
AUG
SD15
CT2
CT8
CT10
BB1
BB2
BB3
LD
SD1
SD2
SD3
SD10
Tissue
Bud
Leaf
Age
Juvenile
Adult

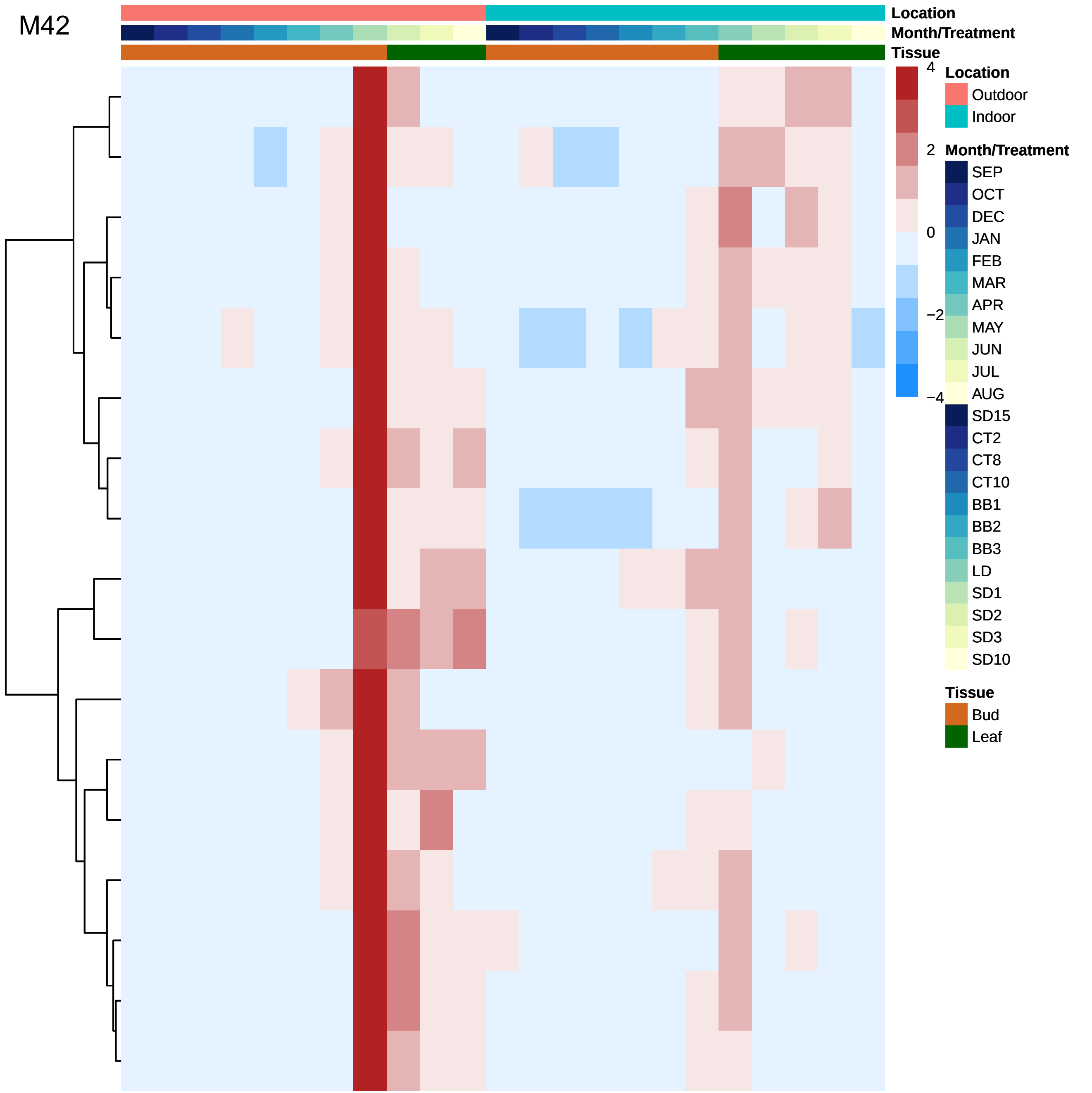

M42
Location
Month/Treatment
Tissue
4
2
0
−2
−4
Location
Outdoor
Indoor
Month/Treatment
SEP
OCT
DEC
JAN
FEB
MAR
APR
MAY
JUN
JUL
AUG
SD15
CT2
CT8
CT10
BB1
BB2
BB3
LD
SD1
SD2
SD3
SD10
Tissue
Bud
Leaf

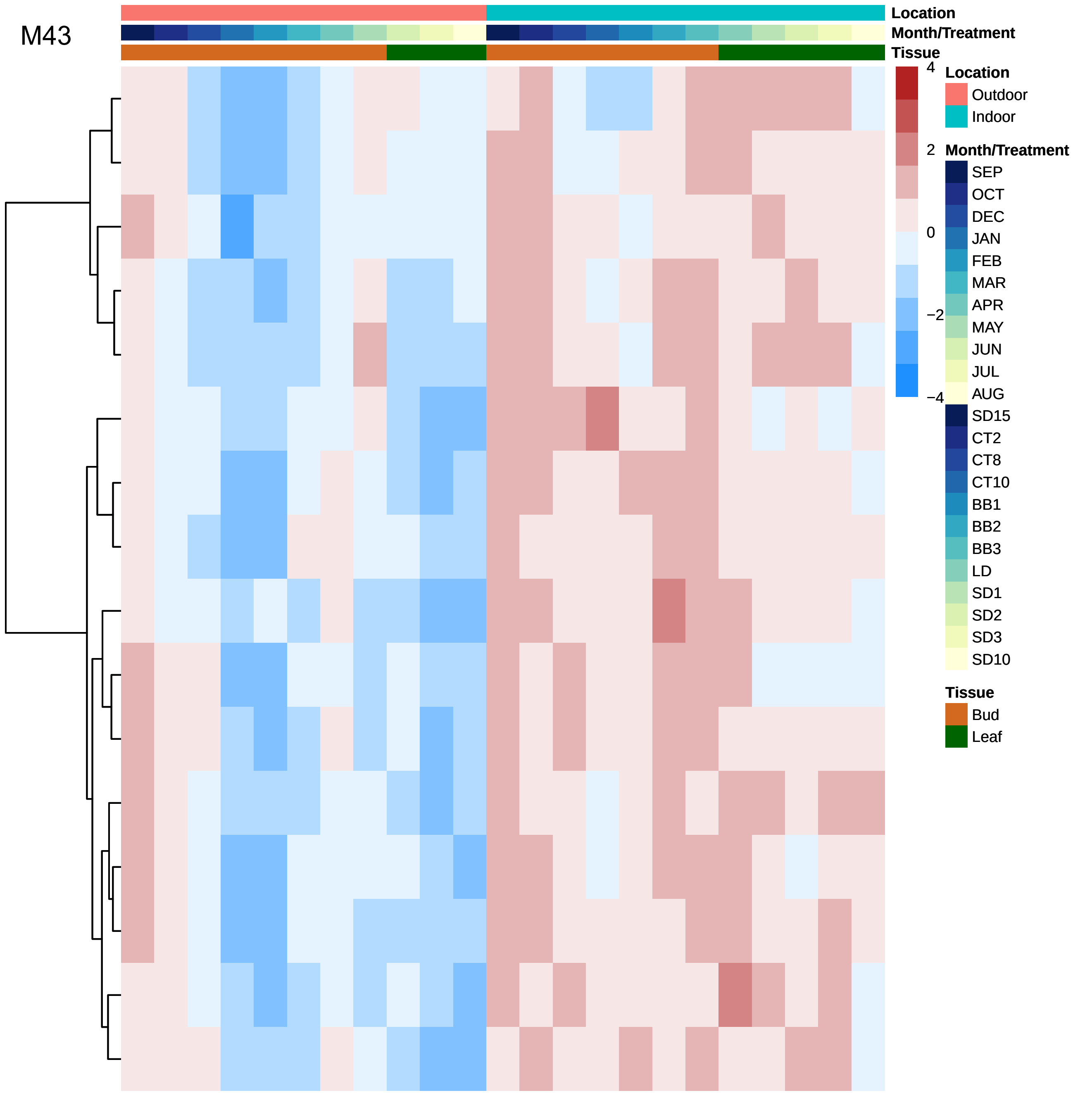

M43
Location
Month/Treatment
Tissue
4
2
0
−2
−4
Location
Outdoor
Indoor
Month/Treatment
SEP
OCT
DEC
JAN
FEB
MAR
APR
MAY
JUN
JUL
AUG
SD15
CT2
CT8
CT10
BB1
BB2
BB3
LD
SD1
SD2
SD3
SD10
Tissue
Bud
Leaf

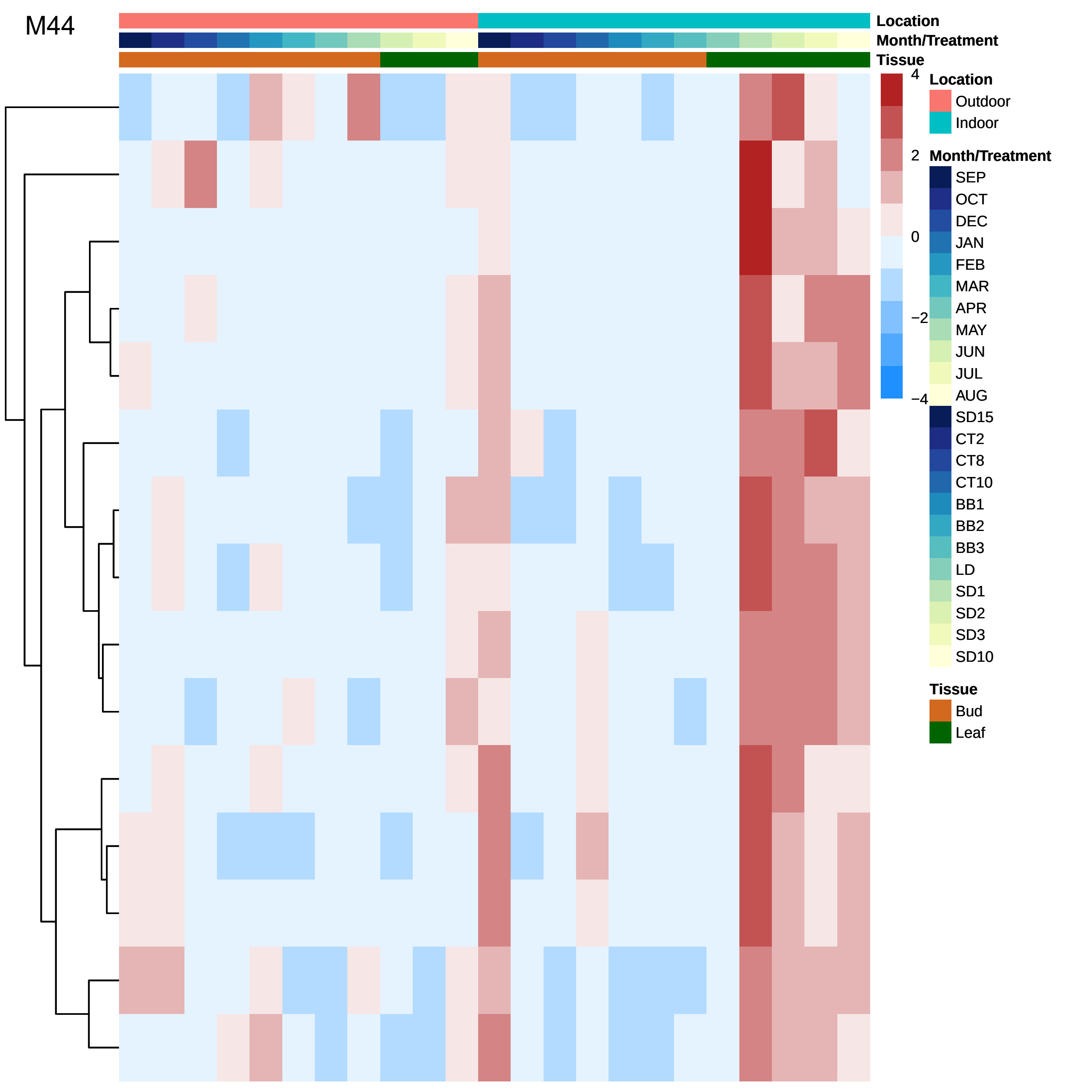
M44
Location
Month/Treatment
Tissue
4
2
0
−2
−4
Location
Outdoor
Indoor
Month/Treatment
SEP
OCT
DEC
JAN
FEB
MAR
APR
MAY
JUN
JUL
AUG
SD15
CT2
CT8
CT10
BB1
BB2
BB3
LD
SD1
SD2
SD3
SD10
Tissue
Bud
Leaf

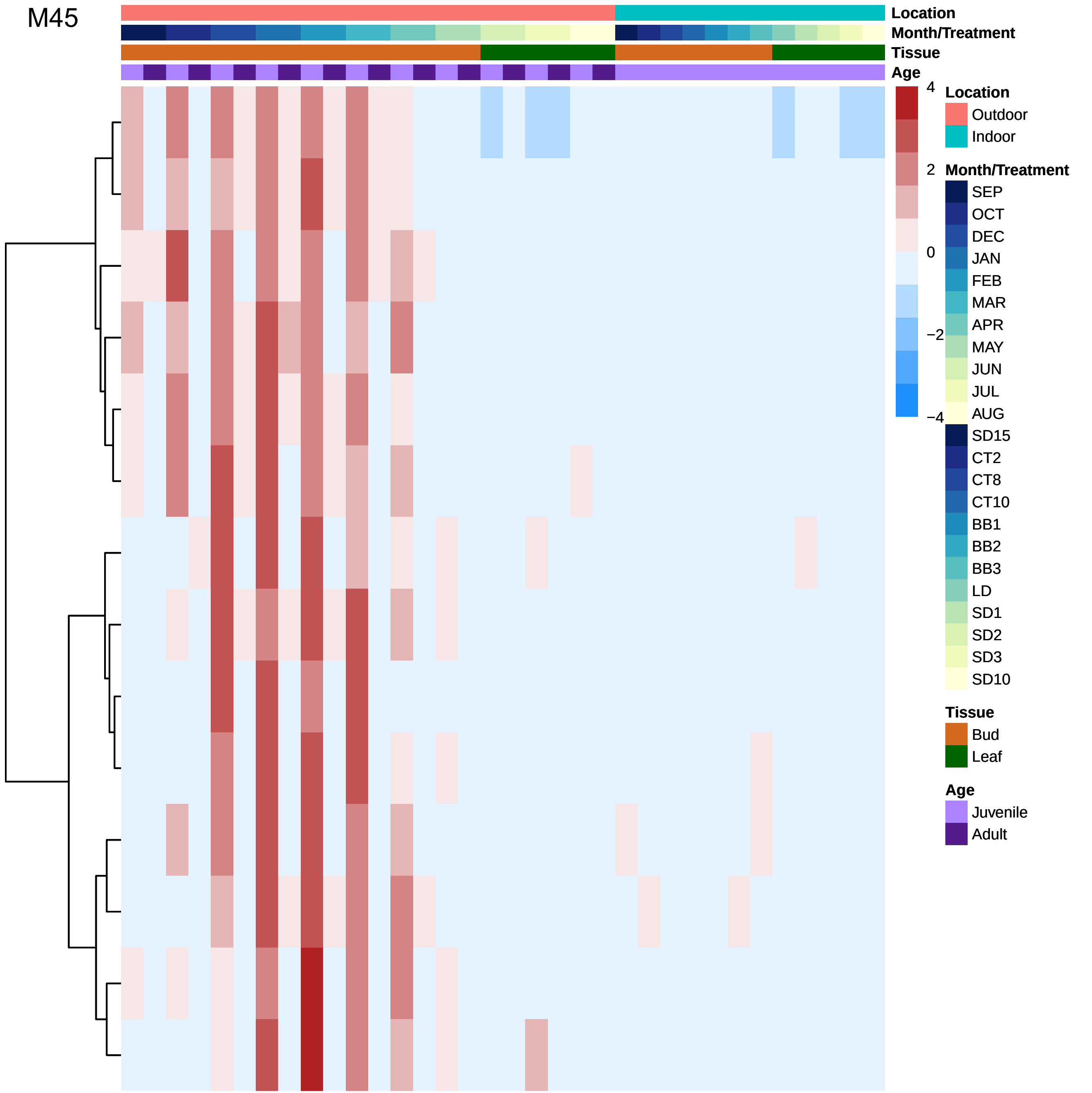

M45
Location
Month/Treatment
Tissue
Age
4
2
0
−2
−4
Location
Outdoor
Indoor
Month/Treatment
SEP
OCT
DEC
JAN
FEB
MAR
APR
MAY
JUN
JUL
AUG
SD15
CT2
CT8
CT10
BB1
BB2
BB3
LD
SD1
SD2
SD3
SD10
Tissue
Bud
Leaf
Age
Juvenile
Adult

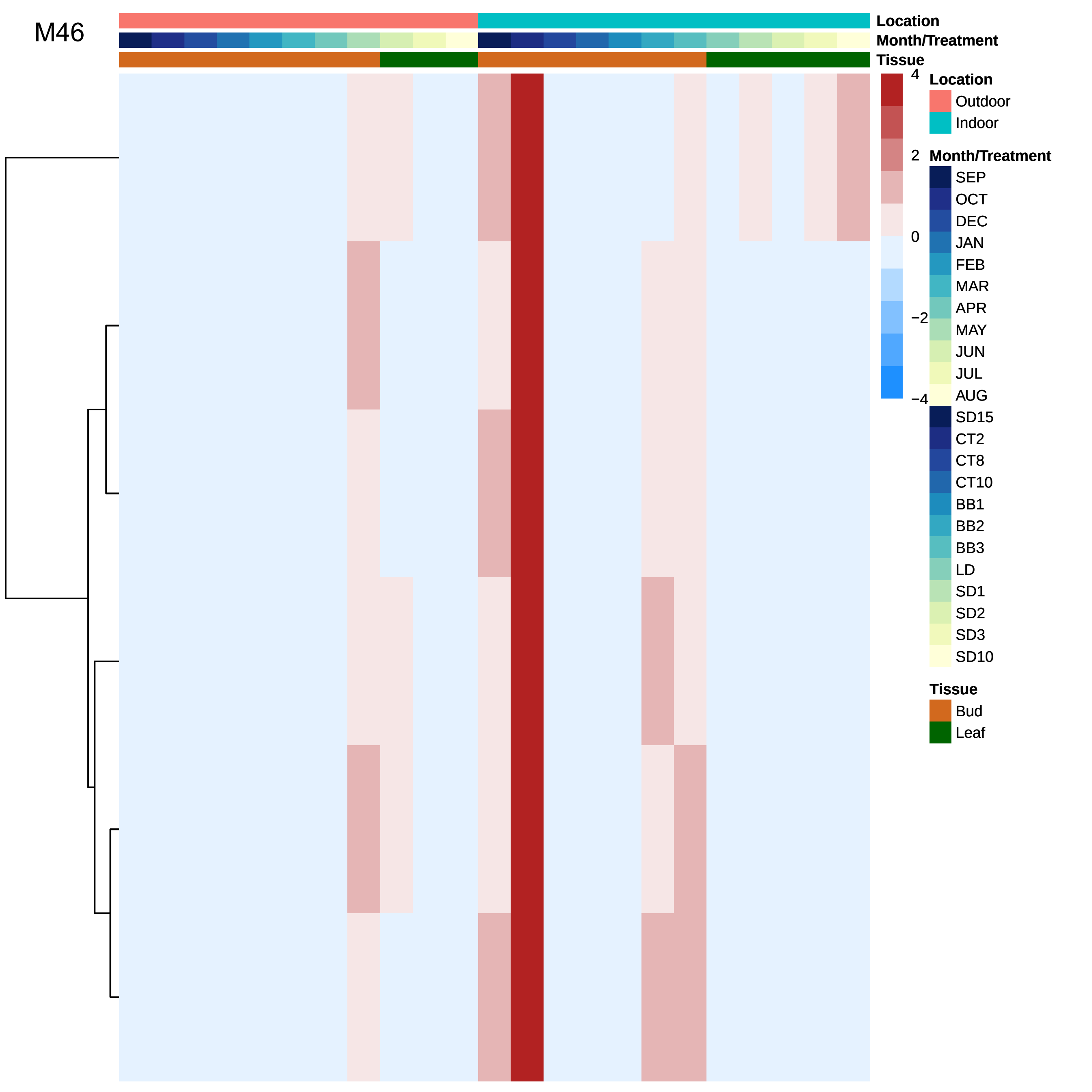
M46
Location
Month/Treatment
Tissue
4
2
0
−2
−4
Location
Outdoor
Indoor
Month/Treatment
SEP
OCT
DEC
JAN
FEB
MAR
APR
MAY
JUN
JUL
AUG
SD15
CT2
CT8
CT10
BB1
BB2
BB3
LD
SD1
SD2
SD3
SD10
Tissue
Bud
Leaf
