# Supplementary material for: A transcriptional roadmap of the yearly growth cycle in Populus trees
Source: Plant Cell. 2025 Aug 20;37(9):koaf208. doi: 10.1093/plcell/koaf208 (PMC12419529; doi:10.1093/plcell/koaf208)
Supplement: koaf208_Supplementary_Data [file Supplementary Data.pdf › Supplementary Data.pdf]

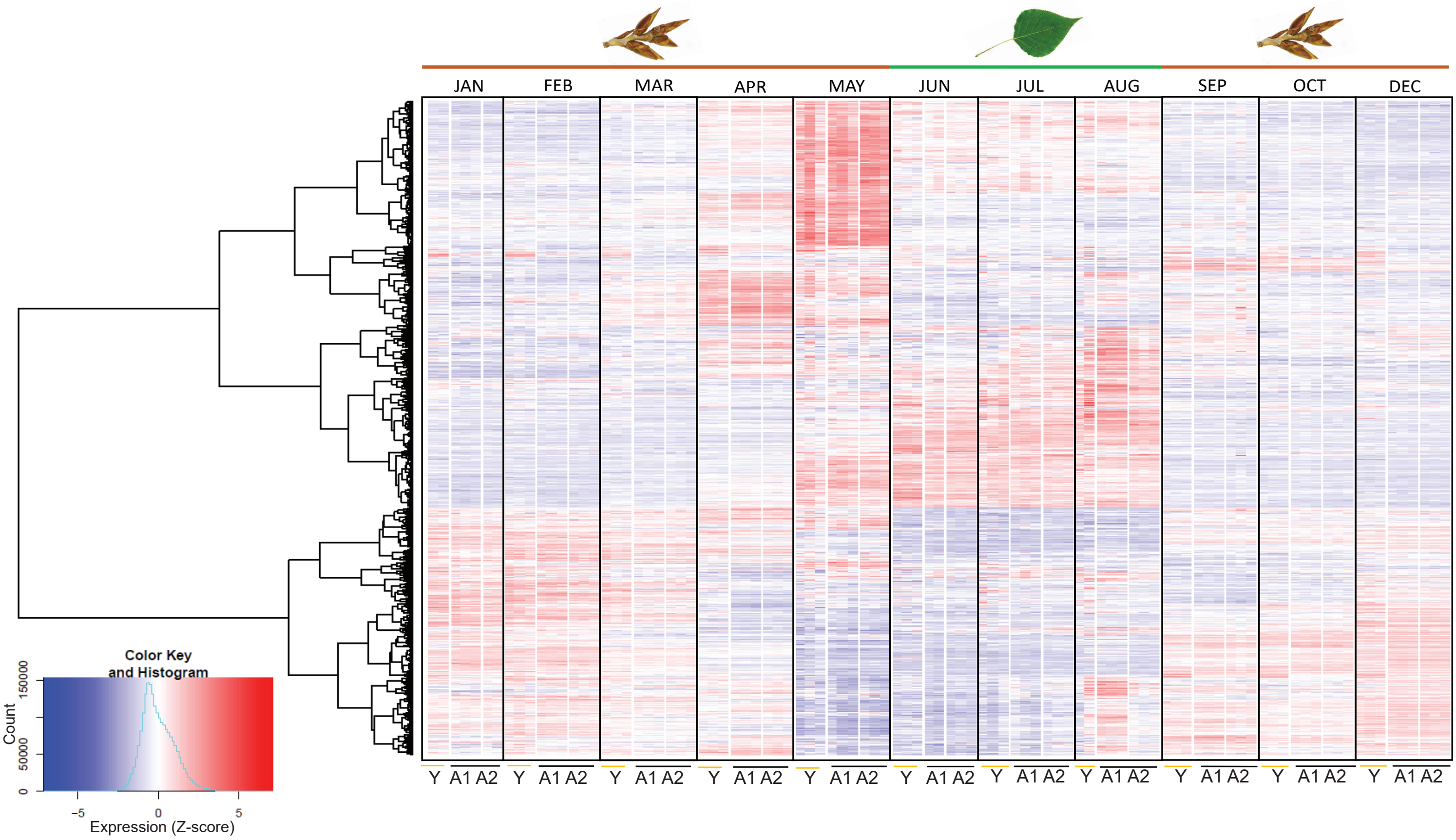

Supplementary Figure 1. Heatmap of all outdoor samples for each month.
A white space separates the biological replicas from the 1-year-old (Y) and 35-year-old trees (A1, A2) for each month. The colour scale bar represents scaled expression (Z-score) and the histogram (light blue line) represents the frequency distribution of Z-score values in the heatmap. Supports Figure 1.
